# Photocaged Oxytocin and Vasopressin Probes to Decipher Neuropeptide Signalling With High Spatiotemporal Resolution

**DOI:** 10.1002/anie.202513373

**Published:** 2026-04-13

**Authors:** Konstantin Raabe, Predrag Kalaba, Xuan Ling Hilary Yong, Greta Crudeli, Sarah Melzer, Erik Keimpema, Victor Anggono, Markus Muttenthaler

**Affiliations:** ^1^ Faculty of Chemistry Institute of Biological Chemistry University of Vienna Vienna Austria; ^2^ Vienna Doctoral School in Chemistry University of Vienna Vienna Austria; ^3^ Faculty of Health, Medicine and Behavioural Sciences, Queensland Brain Institute, Clem Jones Centre for Ageing Dementia Research The University of Queensland Brisbane Australia; ^4^ Department of Neuronal Cell Biology, Center for Brain Research Medical University of Vienna Vienna Austria; ^5^ Department of Molecular Neurosciences, Center for Brain Research Medical University of Vienna Vienna Austria; ^6^ NHMRC Centre for Research Excellence in Mechanisms in NeuroDegeneration – Alzheimer's Disease (MIND‐AD CRE) Brisbane Australia; ^7^ Institute for Molecular Bioscience The University of Queensland Brisbane Australia

**Keywords:** neuropeptides, oxytocin, photocages, photopharmacology, vasopressin

## Abstract

The oxytocin/vasopressin (OT/VP) neuropeptide signalling system is essential for regulating social behaviour, emotion, learning, and memory, and its dysregulation is associated with multiple neurological disorders. However, accurately studying OT/VP signalling in the brain remains difficult due to widespread receptor expression, long peptide half‐lives, and extensive diffusion. To address these challenges, we investigated three classes of photolabile protecting groups—coumarin, nitrophenylpropyl, and borondipyrromethene—to enable precise, light‐triggered OT/VP release. These photocages allow controlled activation with one‐photon (365–527 nm) or two‐photon (730–780 nm) irradiation and do not generate cytotoxic by‐products. Using these cages, we synthesised OT/VP photoprobes and characterised their photopharmacological properties at their neuronal receptors (OTR, V_1a_R, V_1b_R). The coumarin cage proved the most effective, suppressing OT/VP bioactivity until rapid photouncaging enabled on‐demand receptor activation. It excelled in cellular assays, primary rat hippocampal neurones, and ex vivo acute mouse brain slices, demonstrating broad applicability. It is biocompatible, readily incorporated into peptides, and compatible with various neuronal experimental setups. Photo‐uncaging can be performed with inexpensive light‐emitting diode (LED) setups, as well as with extreme precision via two‐photon excitation, enabling investigations of neuropeptide signalling with high spatiotemporal resolution, offering new opportunities to investigate neuropeptide function in health and disease.

## Graphical abstract

Novel and biocompatible photoactivatable oxytocin and vasopressin probes, utilising state‐of‐the‐art photocages, remain inactive in the dark. Light exposure facilitates rapid spatiotemporal receptor activation and phospholipase C (PLC)‐mediated downstream signalling. Figure created in https://BioRender.com.

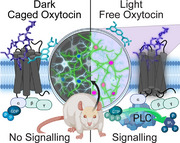

## 1 Introduction

**FIGURE 1:**
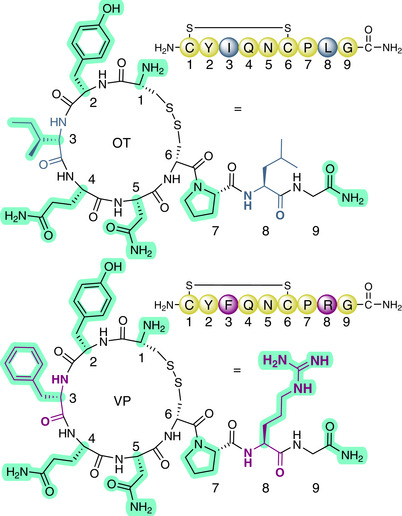
Chemical structure and sequence of neuropeptides oxytocin (OT) and vasopressin (VP). Amino acid differences between OT and VP are highlighted in blue and purple; amino acids crucial for receptor activity are highlighted in light green. *Note*: The chemical structures of OT/VP are shown with *cis* amide bonds throughout this manuscript for illustration purposes only and do not reflect the actual amide bond configuration, which is *trans*.

The cyclic nonapeptides oxytocin (OT: [CYIQNC]PLG*, *C‐terminal amide) and vasopressin (VP: [CYFQNC]PRG*, Figure 1) are highly conserved throughout evolution, originating from the same ancestral vasotocin precursor neuropeptide ∼600 million years ago [1, 2, 3, 4]. Homologues of these neuropeptides exist in both vertebrate and invertebrate species, highlighting the evolutionary importance of the OT/VP signalling system [1, 4]. In mammals, these neuropeptides are produced in hypothalamic neurones and released extrasynaptically from large, dense core vesicles in the dendrites, axons, or soma [5, 6, 7]. Due to a lack of reuptake mechanism, these neuropeptides are not recycled like classical small‐molecule neurotransmitters but remain in the extracellular space until degraded [8, 9]. As a result, they can diffuse into different brain areas and interact with receptors distant from their release site in a process often called diffusion or volume transmission [9, 10, 11, 12, 13].

OT and VP exert their functions by acting on four G protein‐coupled receptors (GPCRs), namely OTR, V_1a_R, V_1b_R, and V_2_R, which are widely expressed in mammalian tissues. These receptors are expressed in both the central and peripheral nervous systems, except that V_2_R is primarily expressed in the kidneys (not in the brain), where it regulates water homeostasis [14, 15, 16, 17]. The interaction of OT with OTR triggers many physiological effects, depending on concentration, location, and tissue type [3, 17]. For example, peripheral OT facilitates labour by activating OTR in the uterus and promotes lactation by acting on OTR in mammary glands, enabling milk delivery to the offspring, both through inducing muscle contractions [14, 18]. VP controls water balance, fluid osmolality, blood pressure, and body temperature in the periphery [14, 19]. In the brain, OT influences prosocial aspects such as pair bonding, mating, social recognition, and parental behaviour, but also emotional responses including trust and affection, memory formation and learning [7, 14, 15, 16, 17]. VP regulates social behaviours often described as opposing or complementary to those of OT [6, 12, 17, 20, 21]. OT can exhibit anxiety‐ and stress‐reducing effects, whereas VP is associated with defensive behaviour like alertness, stress, vigilance and aggression [7, 20, 21, 22, 23]. Dysregulation of the OT/VP signalling balance has been implicated in autism spectrum disorder, addiction, major depressive disorder, and post‐traumatic stress disorder, amongst others [3, 7, 14, 17, 23, 24, 25].

Despite the significance of OT/VP in physiological and (psycho)pathological conditions, their specific modulatory pathways remain not fully understood [12, 21, 26, 27]. Neuropeptide function on the molecular level is usually investigated via in vitro and ex vivo experiments, whereas the macroscopic effects are investigated through behavioural in vivo studies [28]. Given the diverse concentration‐ and location‐dependent functions of the OT/VP systems, studies involving targeted administration and precise receptor activation are crucial for deciphering their signalling pathways and functions [3, 11, 29]. Due to the relatively long half‐lives and diffusion‐driven distribution of neuropeptides through tissue, interpreting neuropeptide signalling in vivo is challenging [8, 11, 30, 31]. After intracerebral injection, similar to endogenous release, concentration‐dependent gradients can cause neuropeptides to diffuse away from the region of interest and activate receptors in adjacent brain areas, impeding the determination of the precise role of OT/VP function at the region of interest [11, 13, 32, 33, 34]. Similar considerations also apply to ex vivo studies. The application of neuropeptide solutions to organotypic or acute brain slices leads to simultaneous, nonspecific receptor activation, internalisation, and desensitisation across the entire slice, making it difficult to obtain precise information from targeted synapses, single neurones, or specific neuronal circuits [35, 36, 37].

To overcome these limitations, new molecular tools are needed [5, 28, 34]. A possible solution is offered by combining techniques such as electrophysiology or behavioural studies with photopharmacology, a compelling field with the specific aim of controlling biological functions via photochemistry, both for fundamental research and therapeutic applications [28, 38, 39, 40]. Light constitutes the ideal trigger for physiological responses, as it is bioorthogonal to cellular processes and can be controlled with high spatiotemporal precision [41]. By implementing photocontrol into a compound of interest, its activity or availability can be regulated within a well‐defined area at a specific time, allowing for the investigation of molecular pathways in healthy organisms and disease models in vitro, ex vivo, and in vivo [34, 42].

Control can be achieved either reversibly through photoswitchable compounds or irreversibly by introducing a suitable photocage, also known as a photolabile protecting group (PPG) [43]. Strategic placement of such photoswitches or photocages in a neuropeptide of interest masks its bioactivity in the dark (non‐irradiated). The photocaged neuropeptide can diffuse through tissue without undesired activation of its cognate receptors. Photo‐uncaging can be performed with high spatiotemporal precision using light at the photochrome's corresponding wavelength, releasing either the unmasked bioactive neuropeptide (irreversible uncaging) or switching the compound back to its bioactive conformation (reversible switching) [39, 41, 44]. Suitable light sources include light‐emitting diodes (LEDs), lasers or fibre optics [45]. By eliminating the need for genetic modification, this molecular approach is well‐suited for investigating the role of endogenous neuromodulators (such as OT/VP) in complex biological settings [34, 46, 47, 48, 49, 50].

Since the first applications of photocaged adenosine triphosphate in 1978 [51], photopharmacology has revolutionised the fields of biology and neuroscience [36, 41, 43, 50]. Over the last decades, various synthetic PPGs and photoswitches have been developed, primarily focused on classical small‐molecule neurotransmitters and ligands [38, 40, 42, 52, 53, 54, 55, 56, 57]. Larger biomolecules such as oligonucleotides, proteins, or peptides were modified only more recently [39, 40, 58, 59, 60, 61, 62], including the rise of the first photoswitchable neuropeptide derivatives of neuropeptide Y [63], OT/VP [64], and orexin B [65], as well as photocaged analogues of enkephalin and dynorphin [30, 66], OT [35, 67], substance P, gastrin‐releasing peptide, and cholecystokinin [35].

Despite recent advances, designing photoswitchable bioactive derivatives remains challenging, with notable shortcomings. The pharmacophore must tolerate the incorporated photochromic moiety in one configuration, while photoisomerisation must induce sufficient structural changes to switch to an inactive photoisomer. However, the light‐induced switch between the two photoisomers is often only minor (<100‐fold) and incomplete [64, 68, 69]. While these photoprobes are still suitable for in vitro investigations with precisely controlled and homogeneously distributed concentrations, they reach their limitations in heterogeneous ex vivo and more complex in vivo systems [70]. Photocaging, on the other hand, is an attractive strategy, as it can effectively mask the pharmacophore of neuropeptides, while photo‐uncaging releases the bioactive parent peptide, mimicking physiological release [43]. The concentrations can furthermore be adjusted by modulating light intensity and pulsing frequency [40].

A PPG can be incorporated into peptides either at the C‐ or N‐terminus as carbamates, esters or amides or into the backbone via alkylation of the amide bond N‐H [35, 59, 71]. If a heteroatom is present at their sidechain, amino acids can also be masked as esters, thiocarbonates, thioethers, ethers, carbamates, amides or amines [58, 71, 72]. Whether a PPG is compatible with the selected functional group mainly relies on the cleavage mechanism. Early PPGs used for applications in neuroscience often require highly energetic UV‐C (200–280 nm) and UV‐B (280–320 nm) light, which not only has low tissue penetration but is also often harmful to living organisms [52, 73, 74, 75, 76, 77, 78]. Exposure to UV light can be mutagenic by damaging DNA, producing oxidative stress through the generation of singlet oxygen, inhibiting protein synthesis via the induction of ribotoxic stress, or inducing cell death [79, 80, 81]. While there is a trend to move to PPGs with more benign cleavage wavelengths in the UV‐A region (320–400 nm) or visible light (400–700 nm), with improved quantum yields for minimised light exposure times, many of these compounds produce cytotoxic cleavage products such as nitroso‐benzaldehyde or nitroso‐aryl ketone derivatives, creating biocompatibility issues [35, 40, 44, 67, 73, 75, 82, 83, 84, 85]. Thanks to the tremendous efforts of several research groups, a variety of PPGs with different cleavage mechanisms and kinetics, as well as absorbance maxima, quantum yields, and less toxic cleavage products, are available today [39, 44, 86]. These distinguish themselves through advantages, limitations, and unique features that render them more or less suited for a given task or target. Despite these achievements, the application of these enhanced PPGs to neuropeptides has been limited [59].

In this work, we expanded the molecular toolbox of neuropeptide photoprobes by implementing and evaluating different state‐of‐the‐art PPGs. Using organic chemistry, we synthesised three distinct classes of PPGs (coumarins, nitrophenethyls, and boron‐dipyrromethenes [BODIPYs]) with different cleavage mechanisms and wavelengths ranging from UV‐A light to the visible part (365–527 nm) for one‐photon (1PE) or two‐photon (2PE) excitation cleavage in the near‐infrared range (NIR: 730–780 nm) of the electromagnetic spectrum. We used these PPGs to mask the bioactivity of OT and VP using a combination of solid‐ and solution‐phase synthetic strategies. This resulted in a series of photocaged OT/VP probes that we (photo)pharmacologically characterised at the brain receptor subtypes (OTR, V_1a_R, V_1b_R), followed by their application and evaluation in cellular and neuronal settings using advanced microscopy techniques.

## 2 Results

### 2.1 PPG Selection and Photoprobe Design

The nine amino acids of OT and VP provide various attachment points for PPGs; however, not all sites are equally suited or chemically and sterically accessible (Figure S1). All residues of OT and VP participate in receptor binding, with the macrocycle between positions 1–6 (OT: [CYIQNC]; VP: [CYFQNC]) buried deep in the binding pocket and the exocyclic three‐residue tail 7–9 (OT: PLG*; VP: PRG*) pointing outward [87, 88]. Although the C‐terminal Gly^9^ is important for agonism and has previously been targeted with a C‐terminal extension approach [35], the enzymatic cleavage site between positions 8 and 9 can produce degradation products that retain some activity [89]. A PPG attached to Gly^9^ could be cleaved enzymatically, resulting in an undesired bioactive metabolite [90]. Furthermore, the tail tolerates some modifications at position 8 without loss of activity [91, 92], leaving the macrocycle as the preferred part for photocaging. All six amino acids of the macrocycle are crucial for receptor binding, with only minor modifications being tolerated [87, 88, 93]. From a synthetic point of view, alkylation of the peptide backbone or the amides of Gln^4^ and Asn^5^ (and the guanidine moiety of Arg^8^ in VP) is theoretically possible, but uncaging might prove difficult and limit the PPGs that are eligible [59]. The sidechain of Tyr^2^ is chemically more accessible, and PPG introduction can be achieved either by ether formation via alkylation or by carbonate formation via an S_N_2 reaction [67]. However, the instability of the carbonate linkage against hydrolysis under physiological conditions and the slow cleavage of PPGs attached via alkylation make Tyr^2^ less attractive. By contrast, the nucleophilicity of the N‐terminal amine and ease of late‐stage modification of the peptides on resin or in solution made the N‐terminus the preferred choice for photocaging. Introducing a PPG at α‐amine of Cys^1^ is also expected to mask the bioactivity of OT/VP, while protecting against enzymatic degradation at the N‐terminus [32, 67, 94, 95, 96, 97, 98]. Hence, based on the pharmacological implications, chemical feasibility, and ability to attach different PPGs independent of the cleavage mechanism, this was the most promising approach and allowed us to directly compare the performance of the individual PPGs.

**SCHEME 1:**
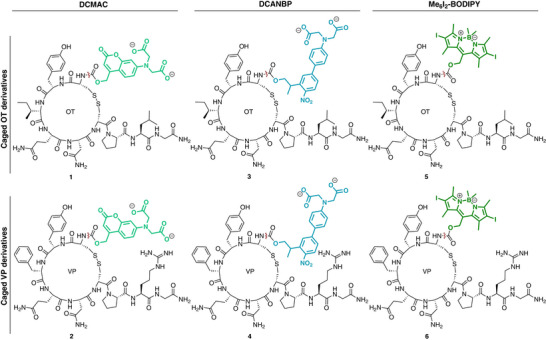
Overview of synthesised PPG‐neuropeptides. PPGs are highlighted in colour and bolded. Carboxylic acid moieties of DCMAC‐ and DCANBP‐derivatives are deprotonated at physiological pH. The red wiggly line indicates the respective photocleavage sites releasing the native ligands.

While some prominent photocages such as *o*‐nitrobenzyl (*o*‐NB) or 6‐nitroveratryloxycarbonyl (NVOC) are commercially available and have been used by others to cage OT at Tyr^2^ or Cys^1^, they also come with drawbacks, such as biocompatibility and photocleavage concerns due to potentially cytotoxic cleavage products and lower quantum yields in the UV‐A and Vis regions (*o*‐NB: *ϕ* = 0.001–0.03; NVOC: *ϕ* = 0.001–0.08), respectively [44, 67]. To create probes that could be more attractive for prospective in vivo applications, we selected three classes of PPGs that we deemed suitable for biological systems for investigation (Scheme 1), covering different uncaging wavelengths and mechanisms (Figure S2). Although red‐light activation is ideal due to reduced phototoxicity and improved tissue penetration, such PPGs typically require large, conjugated π‐systems [40, 46, 86, 99]. This can result in solubility issues and increased injection volumes for delivery, limiting in vivo applicability. Hence, we prioritised PPGs with rapid 1PE cleavage in the blue‐to‐green region of the UV/Vis spectrum (∼400–550 nm) that did not produce cytotoxic cleavage products. The dicarboxymethyl amino coumarin (DCMAC) group exhibits high quantum yields (*ϕ* = 0.29), facilitating rapid uncaging with UV‐A and blue light (365–405 nm), and photocleavage regenerates the original, non‐cytotoxic PPG [44, 100]. Additionally, DCMAC has been developed for 2PE cleavage at ∼740 nm [100]. As the second PPG, we selected the dicarboxy amino nitrobiphenylpropyl (DCANBP) group, as it offers superior quantum yields (*ϕ* = 0.15) compared to conventional nitrobenzyl derivatives and fast 1PE cleavage at 405 nm [57]. Attachment of the compound in the δ‐position with respect to the nitro‐group results in the formation of non‐cytotoxic nitrostyrene derivatives upon cleavage [44]. By contrast, PPGs, such as classic *o*‐NB or NVOC, are attached in the γ‐position, and cleavage can generate cytotoxic nitrosoaldehydes or ketones [40, 57, 58, 73, 85]. The DCANBP PPG has been specifically designed and tested for 2PE cleavage with NIR light (∼800 nm) [57, 100]. As the third PPG class, we selected hexamethyldiiodo BODIPY (Me_6_I_2_‐BODIPY). It combines high quantum yields (*ϕ* = 0.15‐0.32) and 1PE activation with green light (527 nm), allowing chromoorthogonality with the two other PPG classes, while also regenerating the original, non‐cytotoxic PPG without a significant increase in molecular size [101].

Coumarin and BODIPY derivatives are deprotected via a hetero photo‐solvolysis process triggered by photon‐induced weakening of a σ‐bond and subsequent S_N_1 reaction (Figure S2A_1/2_) [85, 86, 102, 103]. Hence, their cleavage is fast if a suitable nucleophile (e.g. H_2_O) is provided in excess by the solvent, making them applicable in biological (aqueous) systems in vitro and in vivo. Although these PPGs can be attached to most heteroatoms, their cleavage rates are strongly influenced by the stability of the intermediates (the carbocation of the photocage after cleavage) and the leaving group [71, 86, 101, 103, 104]. The cleavage velocity of PPGs following this photo S_N_1 reaction is inversely proportional to the pK_a_‐value of the protected functional group, and therefore, direct alkylation is less suited, and the PPGs are predominantly attached as esters, carbamates, or thiocarbonates [49, 86].

The *o*‐nitrobiphenylpropyl cage classes follow a Norrish‐type II cleavage mechanism (Figure S2B) [44, 73]. Irradiation leads to a biradical transition state, followed by γ‐proton abstraction and formation of an aci‐nitro intermediate and loss of aromaticity. Electron pair rearrangement reinstates aromaticity while releasing the bioactive compound [40, 44, 46, 59].

### 2.2 Synthesis

**SCHEME 2:**
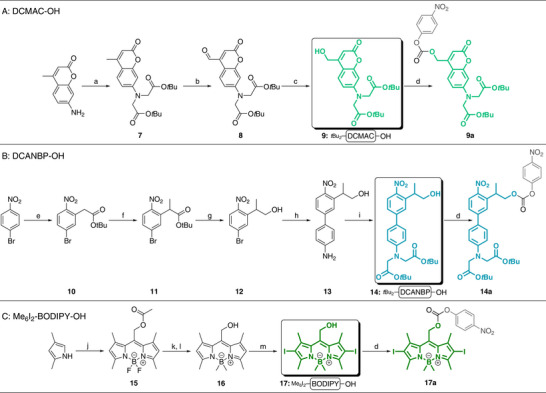
Synthesis of PPGs. Reaction conditions: (A) DCMAC synthesis: (a) DIPEA (neat), *tert‐*butyl bromoacetate, hydroquinone (cat.), 80°C, 5 days, 37%; (b) xylene, SeO_2_, reflux, 24 h, 77%; (c) (1) MeOH, NaBH_4_, RT, 1 h; (2) 0.5 N HCl, 0°C, 63%; (d) DCM, NPC, pyridine, 0°C, 1.5 h, 81%. (B) DCANBP synthesis: (e) (1) DMF, *tert‐*butyl chloroacetate, *tert‐*BuOK, RT, 2 h; (2) 2 N HCl, 0°C, 84%; (f) THF, MeI, NaH, −35°C, 1 h, RT, 24 h, 72%; (g) THF, DIBAL‐H, 0°C, 3 h, 59%; (h) EtOH/ddH_2_O (2:1, degassed), 4‐aminophenylboronic acid•HCl, Bu_4_NBr, Pd(OAc) (cat.), K_2_CO_3_, microwave, 150°C, 10 min, 94%; (i) DMF, *tert‐*butyl bromoacetate, DIPEA, 90°C, 16 h, 43%; (d) DCM, NPC, pyridine, 0°C, 1 h, 92%. (C) Me_6_I_2_‐BODIPY synthesis: (j) (1) DCM, 0°C, acetoxyacetyl chloride, 40°C, 2 h; (2) 0°C, DIPEA, RT, 15 min, (3) 0°C, BF_3_•OEt_2_, RT, 3 h, 27%; (k) (1) ether (absolutised over Na and benzophenone), MeMgI (3 M solution in ether), RT, 16 h; (2) 0°C, sat. aq. NH_4_Cl; (l) THF/ddH_2_O (1:1), LiOH•5H_2_O, RT, 2 h, 61%; (m) THF, NIS, RT, 5 h, 96%; (d) DCM, NPC, pyridine, 0°C, 30 min, 68%. Final PPGs highlighted in bold colour in squares, leaving the group after activation as shown in grey. NPC: 4‐nitrophenyl chloroformate, RT = 25°C.

We combined multiple literature procedures to prepare the coumarin PPG (Scheme 2, **DCMAC‐OH**) [105, 106, 107]. In the first step, the commercial starting material 7‐amino‐4‐methyl coumarin was reacted with *tert‐*butyl bromoacetate in neat DIPEA to obtain the N‐alkylated product **7** in a yield of 37% over 5 days. Prolonging the reaction time or adding NaI did not increase the yield and still resulted in a mixture of the monoalkylated derivative and the dialkylated compound **7**. To introduce the handle for the attachment of peptides, a Riley oxidation with SeO_2_ in xylene was performed, resulting in the aldehyde **8** in 77% yield. When the aldehyde was subsequently reduced to the primary alcohol using NaBH_4_ in MeOH, TLC analysis showed incomplete consumption of the starting aldehyde. Attempts to push the reaction to completion with prolonged reaction times or higher temperatures (30–40°C) resulted in side products with masses corresponding to the loss of *tert‐*butyl esters or lactone opening products. In contrast, using milder reducing reagents such as NaBH(OAc)_3_ or NaBH_3_CN did not yield the desired primary alcohol. A screen of reaction conditions revealed that the best results were obtained with a slight excess of NaBH_4_ (1.75 equivalents) and 3 h at 0°C, resulting in the *tert‐*butyl‐protected PPG *t*Bu_2_‐DCMAC‐OH **9** in 63% yield. Compound **9** was dissolved in DCM and treated with 4‐nitrophenyl chloroformate (NPC) and pyridine to obtain the activated carbonate **9a**.

For the nitrobiphenylpropyl PPG (Scheme 2, **DCANBP‐OH**), we modified a published procedure [57]. An S_N_Ar reaction of 4‐bromonitrobenzene with *tert‐*butyl chloroacetate and *t*BuOK in DMF yielded the precursor of the later attachment point in the *ortho* position to the nitro‐group, resulting in compound **10**. Temperature control and the rate of addition were crucial for the success of the S_N_Ar, as faster addition via cannula transfer or at room temperature (RT: 25°C) resulted in a mixture of side products rather than the desired compound. Only the dropwise addition of 4‐bromonitrobenzene at 0°C resulted in compound **10** with satisfactory yields of 84%. Methylation of the α‐carbon of **10** was carried out with NaH and MeI in THF, resulting in compound **11** with 71% yield, and DIBAL‐H reduction in THF resulted in 59% PPG **12**. To achieve a bathochromic shift of the cleavage wavelength (365 → 405 nm) [108], the π‐system of **12** was extended via a microwave‐assisted Suzuki coupling with 4‐aminophenylboronic acid•HCl, Bu_4_NBr, and Pd(OAc)_2_ in degassed EtOH/ddH_2_O, resulting in 94% compound **13**. Subsequent N‐alkylation with *tert‐*butyl bromoacetate and DIPEA in DMF yielded 43% *t*Bu_2_‐DCANBP‐OH **14**. Similar to the N‐alkylation reaction to obtain the coumarin compound **7**, prolonging the reaction time did not increase the yield. Subsequent activation with NPC and pyridine gave the activated carbonate **14a**.

The BODIPY PPG (Scheme 2, **Me_6_I_2_‐BODIPY‐OH**) was obtained by combining three literature procedures [101, 109, 110]. We carried out a condensation reaction of 2,4‐dimethylpyrrole and acetoxyacetyl chloride in DCM, followed by cyclisation with BF_3_•OEt_2_ and DIPEA to obtain the tetramethyl BODIPY core structure **15**. Despite screening several reaction conditions, including reaction times, temperatures, and equivalents, and taking the utmost care to maintain a dry, inert atmosphere during the reaction, the compound could not be obtained in the reported yields (65–75%) [109, 110] but instead reached ∼5%. Only after changing the provider of 2,4‐dimethylpyrrole to Tokyo Chemical Industry (TCI), which delivered the starting material in sealed ampules, did the yields of BODIPY **15** increase to 27%. While we used dry reagents from Sure/Seal bottles, distilling acetoxyacetyl chloride and DIPEA might further increase yields by removing residual impurities, such as trace amounts of acids or secondary amines, potentially still present from the production process. The boron core was methylated in a Grignard reaction in absolute ether with MeMgI in Et_2_O. The reaction yielded a mixture of the acetylated and deacetylated hexamethyl BODIPY compounds. Through treatment with LiOH•5H_2_O in THF/ddH_2_O, the fully deprotected compound **16** was obtained in 61% yield. Iodination of positions 2 and 6 with N‐iodosuccinimide (NIS) in THF gave the final PPG Me_6_I_2_‐BODIPY‐OH **17** in 96% yield, and after NPC activation, the activated carbonate **17a** was obtained in 68% yield.

**SCHEME 3:**
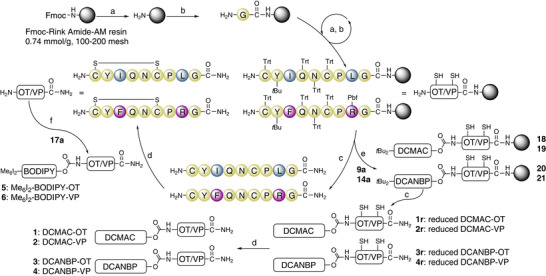
SPPS and caging of OT and VP. Reaction conditions: (a) (1) 1 min DMF flow‐wash; (2) 2 × 1 min piperidine/DMF (1:1); (3) 1 min DMF flow‐wash. (b) HATU (0.5 M in DMF)/amino acid/DIPEA (3.95/4/4 with respect to resin loading), RT, 15 min. (c) (1) 1 min DCM flow‐wash; (2) TFA/ddH_2_O/TIPS (90/5/5), RT, 2 h; (3) N_2_‐stream, Et_2_O, 0°C; (d) (1) 0.1 M NH_4_HCO_3_ buffer (pH = 8.2), RT, 24 h, 2) TFA; Yields after folding and RP‐HPLC: 18% **1**, 17% **2**, 28% **3**, 12% **4**. (e) DMF, pyridine, **9a** or **14a**. (f) THF, DMF, pyridine, **17a**. Yields after photocaging and RP‐HPLC: 56% **5**, 24% **6**. HATU: 1‐[bis(dimethylamino)methylene]‐1*H*‐1,2,3‐triazolo[4,5‐b]pyridinium 3‐oxide hexafluorophosphate, TIPS: triisopropylsilane.

The individual OT and VP neuropeptides were prepared by manual Fmoc‐SPPS (Scheme 3) using standard sidechain‐protected amino acids and a polystyrene‐based Fmoc‐Rink Amide‐AM resin to generate the C‐terminal amides and were ready for subsequent photocaging [111].

The activated PPGs **9a** and **14a** were redissolved in 20% pyridine in DMF and individually added to OT and VP to attach the PPGs on‐resin as N‐terminal carbamates. Once LC‐MS analysis indicated full conversion, the photocaged peptides were cleaved from the resin while simultaneously removing the sidechain protecting groups and the *tert‐*butyl group from the PPGs. The linear (reduced) peptides **1r**, **2r**, **3r**, and **4r** were folded in aqueous 0.1 M NH_4_HCO_3_ buffer (pH = 8.2) and purified via RP‐HPLC, yielding the final compounds DCMAC‐OT/VP **1**/**2** and DCANBP‐OT/VP **3**/**4**.

Due to the acid‐sensitive nature of BODIPY, photocaging on‐resin was not possible since TFA cleavage would dismantle Me_6_I_2_‐BODIPYs. Therefore, conjugation of the BODIPY PPG to the neuropeptides was performed in solution with previously cleaved, folded, and purified OT and VP. After screening combinations of different solvent compositions (ACN, THF, and DMF) and bases (DIPEA, triethylamine, and pyridine), the activated BODIPY carbonate **17a** was redissolved in 10% pyridine in THF and added to a solution of either OT or VP in 15% DMF in THF and stirred at 0°C. The reaction was monitored via LC‐MS, and upon complete consumption of the peptide, the solvent was removed under vacuum. After purification via RP‐HPLC using ddH_2_O/ACN without acid additives to prevent acid exposure, the final compounds Me_6_I_2_‐BODIPY‐OT/VP **5**/**6** were isolated.

### 2.3 Photochemical Characterisation

**FIGURE 2:**
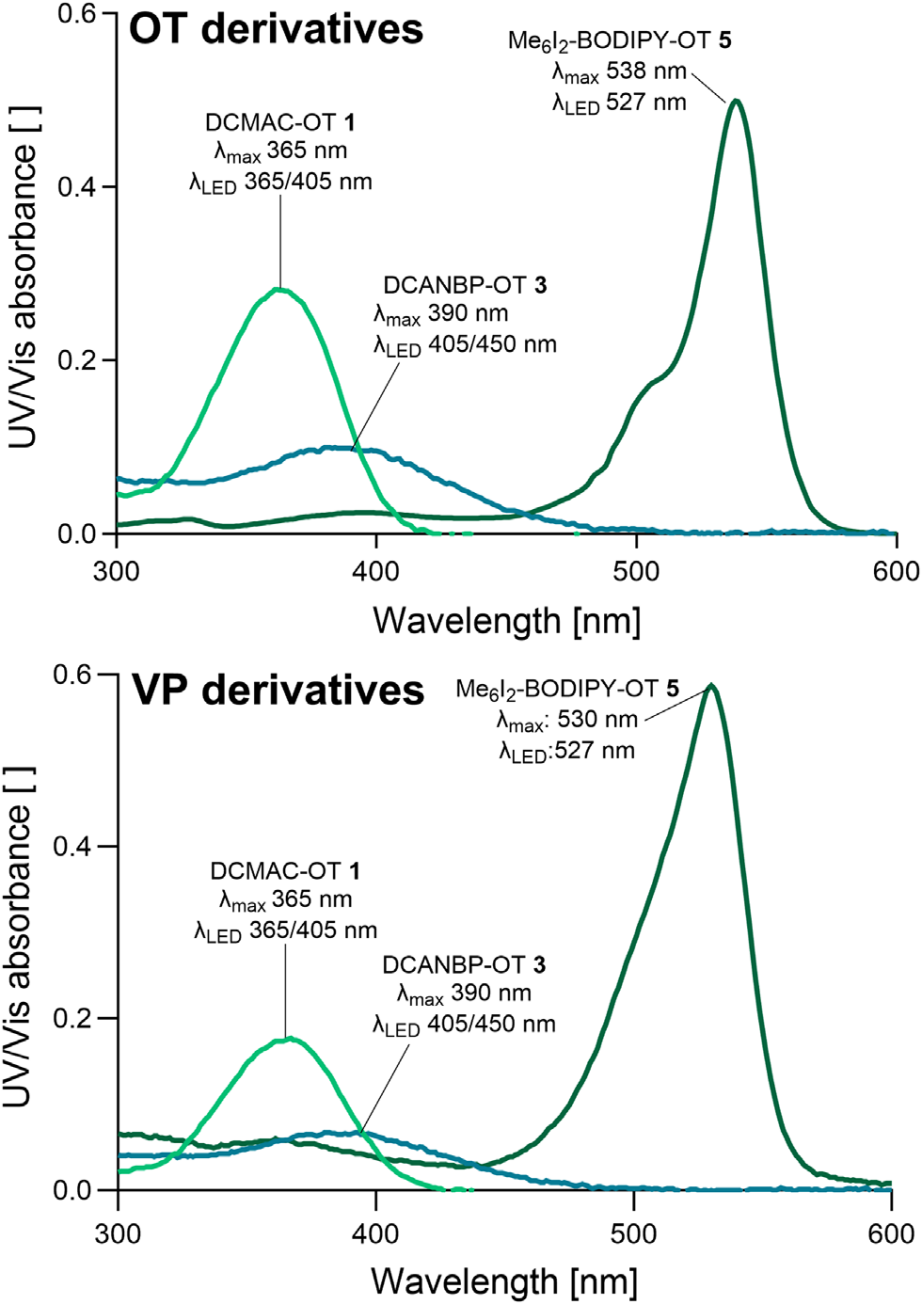
UV/Vis absorbance spectra of the photocaged neuropeptides **1–6**. Measured by NanoDrop 2000 (50 µM in 0.3% ACN/PBS; pH = 7.4). *λ*_max_, absorbance maxima; *λ*_LED_, emission maxima of LEDs selected for uncaging experiments.

To determine the optimal cleavage wavelengths for the photocaged neuropeptides, we prepared 50 µM solutions of **1–6** in 0.3% ACN in phosphate‐buffered saline (PBS) and recorded the absorbance spectra between 300 and 600 nm (Figure 2) with a NanoDrop 2000 and purchased LEDs with peak emission wavelengths close to the absorbance maxima of the compounds.

The DCMAC conjugates **1** and **2** displayed absorbance maxima at 365 nm, with a shoulder extending to 405 nm, corresponding to cleavage wavelengths spanning UV‐A to purple light. The DCANBP derivatives **3** and **4** exhibited absorbance maxima at 390 nm, accompanied by a broad shoulder extending to 450 nm, enabling cleavage under purple‐to‐blue light. The absorbance maxima of around 525 nm of the Me_6_I_2_‐BODIPY compounds indicated possible cleavage with green light.

**FIGURE 3:**
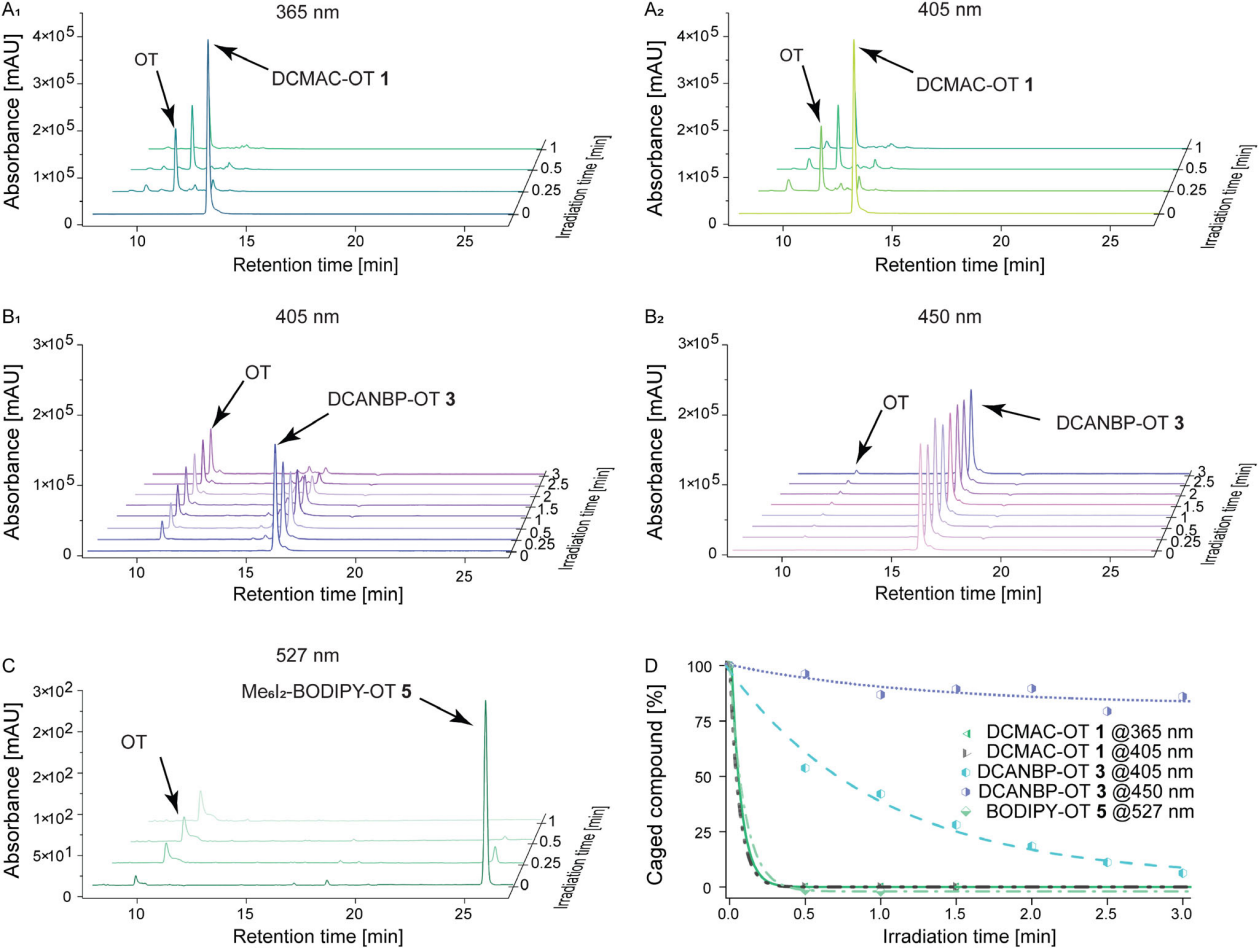
Uncaging of photocaged OT derivatives. (A_1_) Uncaging of DCMAC‐OT **1** with 365 nm, (A_2_) uncaging of DCMAC‐OT **1** with 405 nm; (B_1_) uncaging of DCANBP‐OT **3** with 405 nm, (B_2_) uncaging of DCANBP‐OT **3** with 450 nm; (C) uncaging of Me_6_I_2_‐BODIPY‐OT **5** with 527 nm; and (D) uncaging overview: peaks of photocaged compounds integrated after irradiation times and normalised to the highest area (*t* = 0). Absorbance measured at 214 nm.

Since the uncaging mechanism only depends on the type of chemical conjugation and point of attachment, the cleavage rate of the PPGs will be the same for caged OT or VP; hence, we only evaluated the OT derivatives **1**, **3,** and **5** at 50 µM in PBS by irradiating the samples individually first for 15 s, followed by 30 s increments up to 3 min. Uncaging was investigated with 365 and 405 nm for DCMAC‐OT **1**, 405 and 450 nm for DCANBP‐OT **3**, and 527 nm for Me_6_I_2_‐BODIPY‐OT **5**. The uncaging process was monitored by analytical C_18_‐RP‐HPLC at 214 nm (peptide bond wavelength) and quantified by normalising the maximum absorbance of the caged derivatives at *t* = 0 min and comparing it over time (Figure 3).

While uncaging of DCMAC‐OT **1** was quantitative after 15 s at 365 and 405 nm, DCANBP‐OT **3** could only be uncaged at 405 nm, which was not quantitative even after prolonged irradiation (58% after 60 s and 94% after 3 min). Me_6_I_2_‐BODIPY‐OT **5** performed similarly well to DCMAC‐OT **1** and was quantitatively uncaged at 527 nm after 30 s. While physiological conditions favour the photo‐S_N_1 mechanism for the coumarin and BODIPY derivatives, uncaging of DCANBP derivatives was slow in PBS. As reported in the literature, an inverse behaviour is observed when uncaging is performed in 30% ACN/ddH_2_O (Figure S3) [73]. Furthermore, we investigated the dark stability of these probes under physiological conditions by incubating the peptide solutions in PBS at pH 7.4 and 37°C in brown‐light‐blocking LC‐MS vials. RP‐HPLC analysis revealed full stability for DCMAC‐OT **1** and DCANBP‐OT **3**, while 18% of Me_6_I_2_‐BODIPY‐OT **5** hydrolysed over the timespan of 12 h (Figure S4).

### 2.4 Cytotoxicity

**FIGURE 4:**
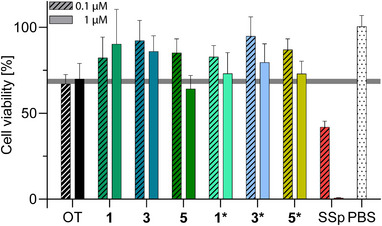
Cytotoxicity evaluation via MTT assay. MTT assays were performed on HEK‐293 wild‐type cells with OT, caged (**1**, **3**, **5**) and in situ uncaged OT derivatives (1*, 3*, 5*), staurosporine (SSp) and vehicle (PBS). In situ uncaging was performed for 60 s with LEDs. Each bar represents three independent measurements with technical triplicates. Cell viability results were normalised to medium with cells (100%) and medium without cells (0%). Compounds and SSp were assessed for 48 h at 0.1 and 1 µM, indicated by patterned and filled bars, respectively; asterisks indicate in situ uncaged derivatives.

While we based our PPG selection in part on their lack of cytotoxic cleavage products, we nonetheless performed cytotoxicity assays to validate the biocompatibility of our probes. Utilising the enzymatic reduction of the yellow 3‐(4,5‐dimethylthiazol‐2‐yl)‐2,5‐diphenyltetrazolium bromide (MTT) to its purple formazan derivative, which only happens in metabolically active cells, we assessed the viability of HEK‐293 cells after treatment in a colourimetric assay (Figure 4) [112]. To mimic biological conditions, we performed the experiments using both the caged compounds (**1**, **3**, **5**) and the in situ uncaged mixture (1*, 3*, 5*), which included the released peptides plus any cleaved PPGs (60 s light irradiation with LEDs, 1* and 3* at 405 nm, 5* at 527 nm). This approach accounts for the possible formation of side products during photolysis, in addition to the released OT and uncaged PPGs. A direct comparison with unmodified OT allowed the assessment of any cytotoxic effects arising from the PPGs. Staurosporine (SSp), an apoptosis‐inducing bacterial alkaloid, was used as a positive cytotoxic control [113]. While in vitro and ex vivo assays usually require only a few hours, the compounds may remain present for prolonged periods during behavioural studies. A 48 h incubation period was chosen because of the slow cell death induced by the SSp cytotoxic control. Most photocaged OT probes (except for 1 µM **5**) exhibited higher cell viability than unmodified OT (Figure 4, grey horizontal bar), which is likely related to their inactivity (OT itself reduced cell viability over 48 h). The uncaged compound mixtures exhibited values comparable to those of OT. These results confirmed the biocompatibility of our probes.

### 2.5 Functional FRET Assays—IP_1_

To validate our design strategy for PPG attachment, we investigated the dark activity of the caged compounds (**1**‐**6**) in cellular functional assays using HEK‐293 cell lines stably overexpressing the human OTR and VPR subtypes found in neuronal tissues (hOTR, hV_1a_R, hV_1b_R), tagged with green fluorescent protein (GFP). Upon ligand binding to such G_q_‐type GPCRs, conformational changes on the cytosolic side of the receptor facilitate the activation of phospholipase C [114]. Phospholipase C, in turn, generates the secondary messenger d‐myo‐inositol 1,4,5‐trisphosphate (IP_3_) that mobilises Ca^2+^ stores and other intracellular actions such as phosphorylation of the cyclic adenosine monophosphate (cAMP) response element binding protein (CREB; pCREB) and signal transduction. IP_3_ is rapidly degraded enzymatically to d‐myo‐inositol 4,5‐bisphosphate (IP_2_), IP_1_, and finally d‐myo‐inositol [115, 116]. This cascade is used in IP_1_ bioassay kits to determine the capability of a compound to activate a G_q_‐GPCR.

**FIGURE 5:**
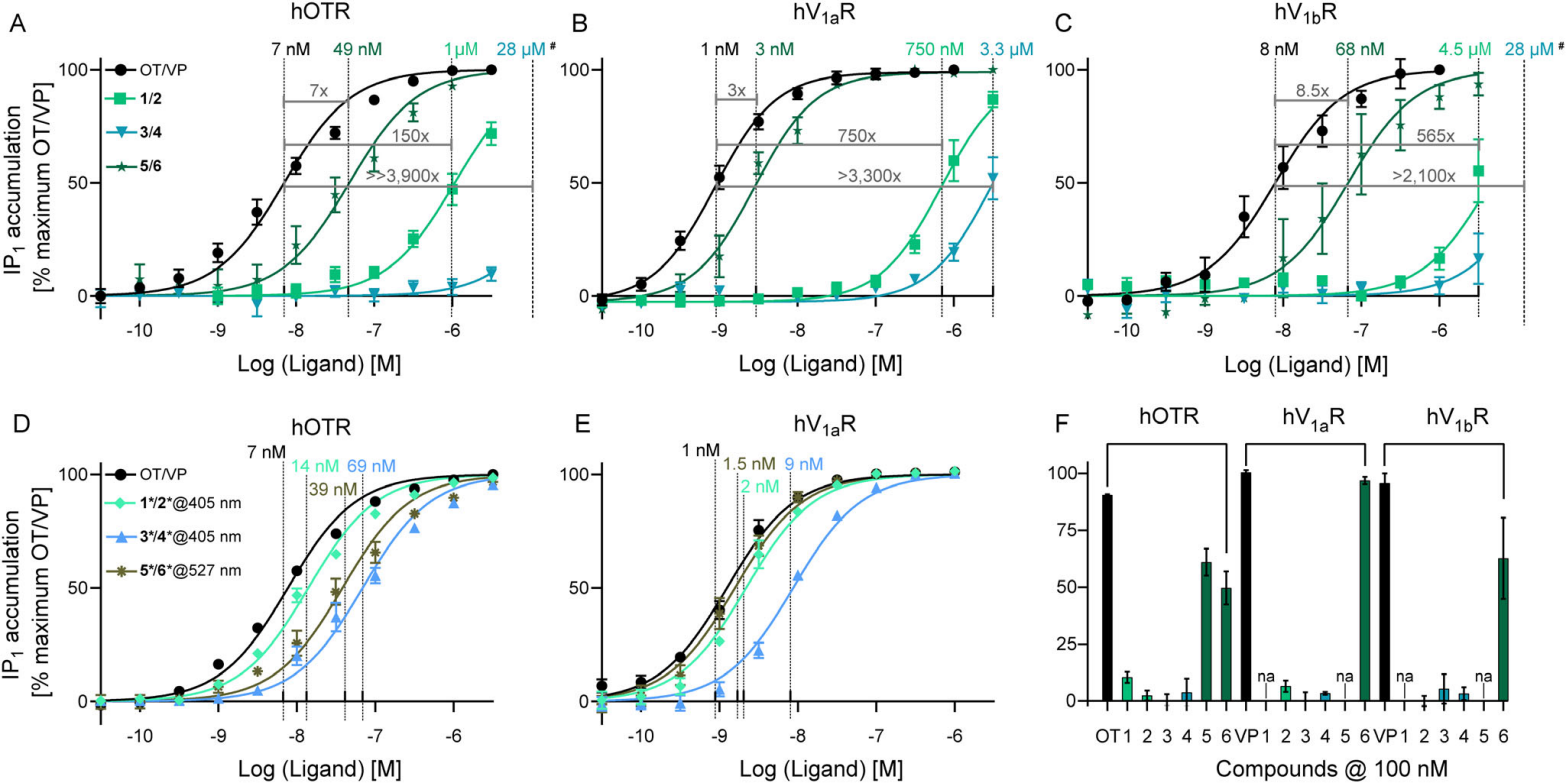
Pharmacological evaluation of photocaged neuropeptides at human OTR, V_1a_R and V_1b_R. Cellular functional IP_1_ assays were performed on HEK‐293 cells stably overexpressing hOTR, hV_1a_R, or hV_1b_R. Each point represents at least three independent measurements with technical triplicates. Results were normalised to OT/VP (100%) and negative control (vehicle; 0%) activity. Error bars indicate the standard error of the mean (SEM). (A) hOTR, (B) hV_1_aR, (C) hV_1b_R, (D) OT derivatives at hOTR after 60 s of in situ uncaging, (E) VP derivatives at hV_1a_R after 60 s of in situ uncaging, and (F) 100 nM at each receptor. EC_50_ values are indicated by dashed vertical lines and presented above with the same colour coding for the corresponding compounds; ^#^ indicate extrapolated values, and numbers on horizontal lines indicate an x‐fold increase of EC_50_ values compared to the parent peptide; asterisks indicate in situ uncaged derivatives; na, no activity at 100 nM.

We tested each of our synthesised compounds (**1**‐**6**) in a dose‐response manner (from 30 pM to 3 µM) on their respective receptors, i.e., **1**, **3**, and **5** on hOTR, and **2**, **4**, and **6** on hV_1a_R and hV_1b_R (Figure 5A–C, Table S2), using OT (OTR) and VP (V_1a_R, V_1b_R) as positive controls. To ensure that uncaging releases the bioactive peptides, we subsequently irradiated aliquots of **1–6** (1*‐6*) in situ for 60 s and repeated the assays using hOTR and hV_1a_R (Figure 5D,E, Table S3). In addition, we performed single‐point measurements of the caged compounds on the complementary receptors to exclude any cross‐activity at higher concentrations (100 nM) (Figure 5F). The photocaged DCMAC‐OT/VP **1**/**2** and DCANBP‐OT/VP **3**/**4** displayed substantially higher EC_50_ values (150 → >3900‐fold at hOTR; 565 → 3300‐fold at hV_1x_R) than their parent peptides, confirming that our photoprobe design effectively masked OT and VP bioactivity. By contrast, the Me_6_I_2_‐BODIPY‐OT/VP designs **5**/**6** exhibited only a 3‐ to 9‐fold rightward shift in EC_50_ compared to OT and VP, revealing a smaller PPG masking/(in)activity window. All peptides presented increased activity after uncaging, with DCMAC‐OT/VP 1*/2* coming closest to the EC_50_ of the parent peptides and a 75/340‐fold increase in potency for hOTR and hV_1a_R, respectively (Figure S6A,D). In situ uncaging of DCANBP‐OT/VP 3*/4* led to a 400/385‐fold increase in activity, representing ∼10‐fold higher EC_50_ values than those of the parent peptides (Figure S6B,E). This residual rightward shift for 3*/4* reflects incomplete photocleavage after 60 s irradiation, whereas 1*/2* achieved full cleavage within the same time, yielding nearly identical EC_50_ values. (Figure S6B,E). The irradiated Me_6_I_2_‐BODIPY‐OT/VP derivatives 5*/6* displayed only a minor increase in activity (∼2‐fold) since they already exhibited undesired activity in the dark (Figure S6C,F). In the single‐point experiments to measure cross‐reactivity, most caged photoprobes exhibited no activity at 100 nM, except for Me_6_I_2_‐BODIPY‐VP **6**, with ∼50% activation at hOTR.

### 2.6 LEDs Facilitate Temporal Neuropeptide Release From Caged Compounds

**FIGURE 6:**
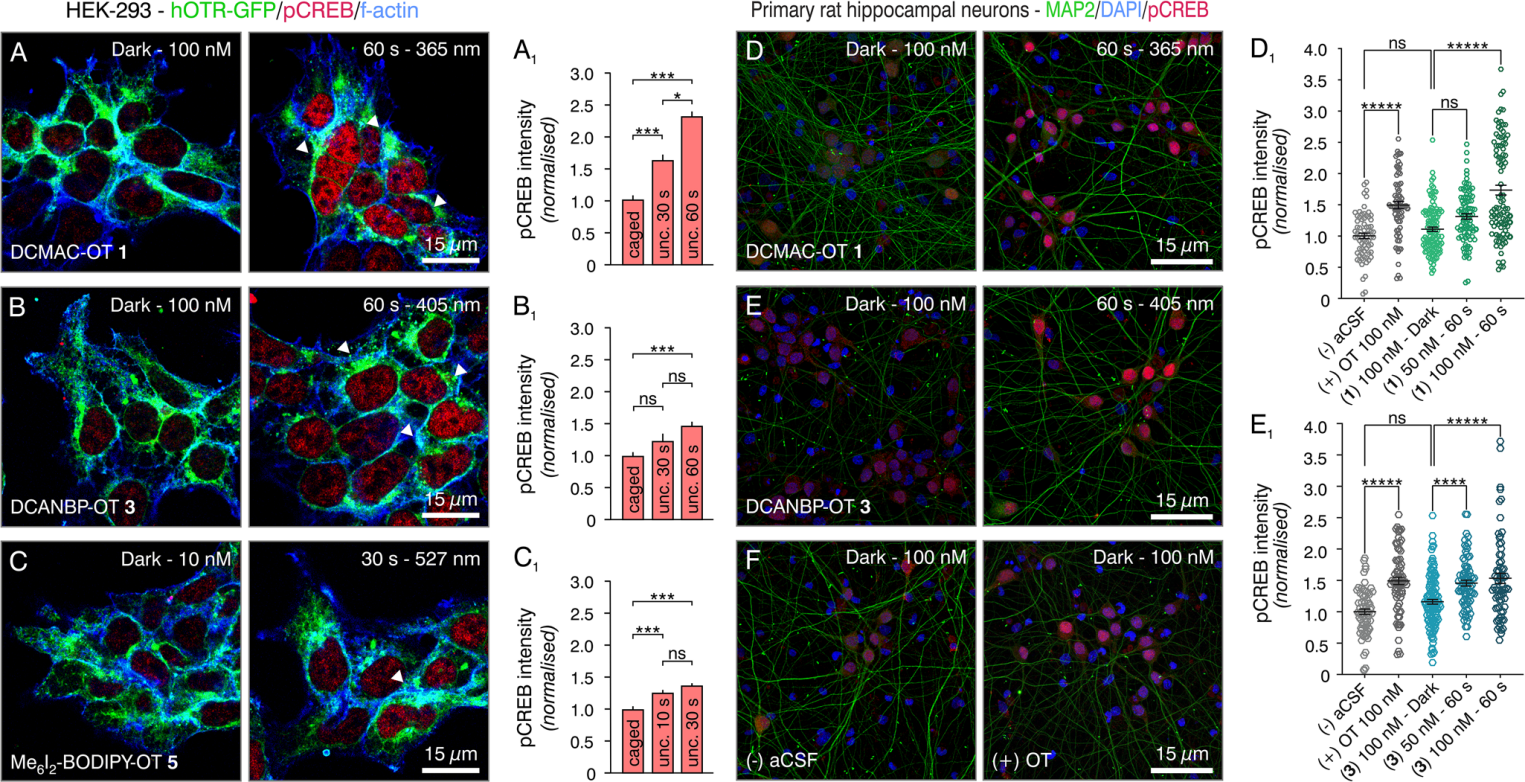
In vitro evaluation of the bioactivity of OT photoprobes, caged and uncaged. Left on HEK‐293 cells overexpressing hOTR‐GFP. Phosphorylation of CREB (red) was quantified after immunocytochemistry of *n* = ∼200 cells over two coverslips and normalised to caged treatment. (A, A_1_) DCMAC‐OT **1**, 100 nM, irradiation time 30 s and 60 s at 365 nm. (B, B_1_) DCANBP‐OT **3**, 100 nM, irradiation time 30 s, 60 s at 405 nm. (C, C_1_) Me_6_I_2_‐BODIPY‐OT **5**, 10 nM, irradiation time 10 s, 30 s at 527 nm. Note the transfer of hOTR‐GFP signals (green) from the membrane (blue) into the cytosol (arrowheads indicate green puncta), indicating receptor internalisation after uncaging. Right on primary rat hippocampal neurones. Nuclei were stained with DAPI (blue), and dendrites with antibodies for MAP2 (green). Phosphorylation of CREB (red) was quantified after immunocytochemistry of *n* = ∼100 cells over two coverslips and normalised to the negative control (aCSF). (D) DCMAC‐OT **1**, 100 nM, dark and irradiated at 365 nm for 60 s. (D_1_) Quantification and statistical evaluation of pCREB signal intensity of DCMAC‐OT **1** 100 nM in the dark and 50/100 nM irradiated at 365 nm for 60 s. (E) DCANBP‐OT **3**, 100 nM, dark and irradiated at 405 nm for 60 s. (E_1_) Quantification and statistical evaluation of pCREB signal intensity of DCANBP‐OT **3** 100 nM in the dark and 50/100 nM irradiated at 405 nm for 60 s. (F) Negative control (aCSF) and positive control (OT, 100 nM) dark; ns, non‐significant; * = *p* < 0.05; *** = *p* < 0.001; **** = *p* < 0.0005; ***** = *p* < 0.0001.

To consolidate the results from the IP_1_ assays and validate uncaging in a biologically relevant setting, we performed in vitro experiments using the OT photoprobes **1**, **3**, and **5** on the same HEK‐293 cells overexpressing hOTR‐GFP. Solutions of the caged peptides (**1** and **3**: 100 nM; **5**: 10 nM) were added to the cells in black 24‐well plates. Subsequently, the wells containing the compounds—except for the dark control (no irradiation; dark)—were individually irradiated (**1** and **3**: 30 and 60 s; **5**: 10 and 30 s) with LEDs at the corresponding wavelengths without further delay. The concentrations and irradiation times were selected based on the IP_1_ assay results and uncaging experiments. The cells were placed into an incubator for 1 h at 37°C in the dark and subsequently fixed and stained with antibodies against GFP (hOTR‐GFP internalisation upon binding of OT), f‐actin (cell borders), pCREB, and the nuclei, and visualised via confocal laser scanning microscopy (Figure 6A–C; Figure S7–S9).

Compared to the irradiated photoprobes, the photocaged OT derivatives did not activate OTR; OTR remained localised on the cell surface, and only residual pCREB signals (red) were observed. By contrast, upon light irradiation, hOTR‐GFP (green) started migrating into the cytosol (Figure S9) due to receptor internalisation in response to released OT activating hOTR. For DCMAC‐OT **1** and DCANBP‐OT **3**, this effect was pronounced, while Me_6_I_2_‐BODIPY **5** only resulted in minor internalisation. In terms of pCREB activity, an irradiation time‐dependent increase in signal intensity for all derivatives compared to their dark controls was observed (Figure 6A_1_,B_1_,C_1_).

To further investigate the application scope of our photoprobes for ex vivo and in vivo applications, we applied DCMAC‐OT **1** and DCANBP‐OT **3** to primary hippocampal neurones isolated from rat embryos known to express OTR [117] (Me_6_I_2_‐BODIPY‐OT **5** was not used due to its limited (in)activity window). These neuronal cells are less resilient than HEK‐293 cells and express OTR at endogenous levels, which are significantly lower than those achieved with the previously used overexpression cell system. We were particularly interested in whether we could generate a quantifiable readout at native receptor expression levels and if neuronal cellular functions were affected by the irradiation process.

We used 100 nM solutions of the caged peptides **1** and **3** as dark controls (not irradiated) and OT in artificial cerebrospinal fluid (aCSF) as the active, positive control, as well as 50 and 100 nM solutions of **1** and **3** for the irradiation experiments, while aCSF without compounds was used as the negative control. The compound solutions were added to the neurones in black 24‐well plates and irradiated for 60 s with LEDs of the corresponding wavelengths (**1** at 365 nm and **3** at 405 nm). The cells were placed in the incubator for 1 h at 37°C, washed, fixed, and stained with primary and secondary antibodies for microtubule‐associated protein 2 (MAP2; somatodendritic marker) [118], 4′,6‐diamidino‐2‐phenylindole (DAPI; nuclei) and pCREB (Figure 6D–F, Figure S10–S12). After confocal microscopy analysis, the intensity of pCREB signals was normalised to the negative control and quantified (Figure 6D_1_,E_1_).

The blue signals visualise the nuclei of all cells in the primary co‐culture, including neurones, glial cells, and astrocytes, stained by DAPI. The green MAP2 signal visualises neuronal dendrites, while the red signal represents pCREB activity. The dark controls of DCAMC‐OT **1** and DCANBP‐OT **3** showed pCREB activity similar to that of the negative controls, confirming their biological inactivity at concentrations up to 100 nM. Upon irradiation, a significant increase in pCREB signals and translocation to neuronal nuclei, comparable to that observed with OT, was noted, indicating successful uncaging and receptor activation, and mirroring the results from the experiments with HEK‐293 cells. Notably, both the dendrites and the nuclei appeared to remain intact and functional, suggesting that the light exposure did not cause any neuronal damage.

### 2.7 2PE Uncaging Facilitates hOTR Activation With High Spatiotemporal Precision

**FIGURE 7:**
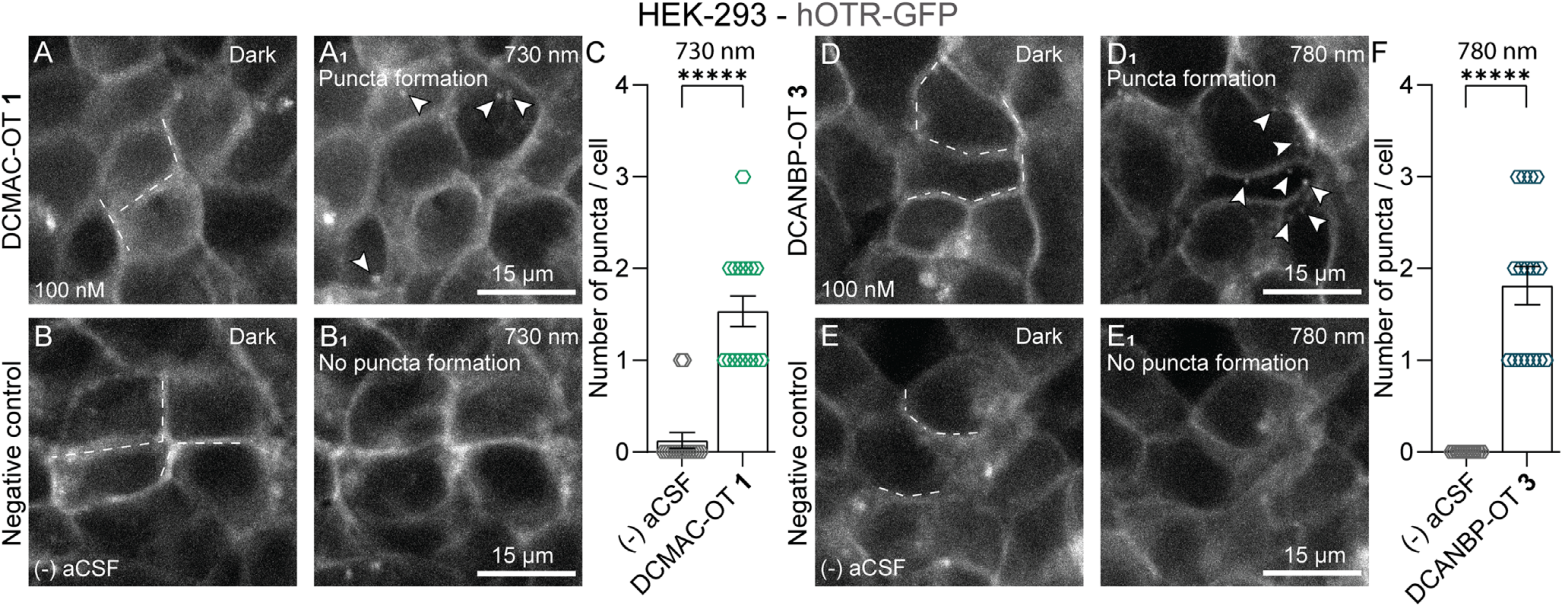
2PE OT photoprobe uncaging during live‐cell imaging using HEK‐293 cells stably overexpressing hOTR‐GFP and a Ti:Sapphire laser tuned to 730/780 nm. (A, A_1_) DCMAC‐OT **1** 100 nM in the dark and 10 min after local irradiation (dashed lines) at 730 nm. (B, B_1_) Negative control (aCSF) in the dark and 10 min after local irradiation (dashed lines) at 730 nm. (C) Quantification of hOTR‐GFP internalisation (white puncta) 10 min after irradiation of aCSF or DCMAC‐OT **1** at 730 nm. (D, D_1_) DCANBP‐OT **3** 100 nM in the dark and 10 min after local irradiation (dashed lines) at 780 nm. (E, E_1_) aCSF in the dark and 10 min after local irradiation (dashed lines) at 780 nm. (F) Quantification of hOTR‐GFP internalisation (white grains; puncta formation) 10 min after irradiation of aCSF or DCANBP‐OT **3** at 780 nm. Arrowheads indicate puncta; puncta formation was quantified for *n* = 15 cells; ***** = *p* < 0.0001.

After demonstrating compatibility with neuronal systems and temporal control of receptor activation via on‐demand OT uncaging, we wanted to investigate the degree of spatial control achievable with our probes. Therefore, we performed 2PE uncaging experiments using the 2PE‐capable OT probes DCMAC‐OT **1** and DCANBP‐OT **3**. To track the extent of OT release, we returned to the hOTR‐GFP HEK‐293 system, which allowed us to quantify receptor internalisation after activation by monitoring hOTR‐GFP translocation as a readout of OT release from the caged derivatives. The HEK‐293 hOTR‐GFP cells were treated with aCSF as a negative control or 100 nM of **1** or **3** in aCSF (Figure 7). Images were acquired before and 10 min after irradiation with a Ti:Sapphire laser tuned to the 2PE wavelengths corresponding to twice the absorbance maxima of each compound (**1**: 2 × 365 nm → 730 nm, 60 pulses, 30 ms, 0.3 Hz; **3**: 2 × 390 nm → 780 nm, 60 pulses, 45 ms, 0.25 Hz). In irradiated samples, we observed pronounced puncta formation (Figure 7A_1_,D_1_; arrowheads), reflecting receptor activation and subsequent internalisation after OT release via 2PE uncaging. This effect was slightly more pronounced with compound **3** than with **1** (inverse to 1PE uncaging) but notably remained confined to cells immediately adjacent to the irradiation site for both compounds (Figure 7, dashed lines). These findings underscore the exceptional spatiotemporal precision of our probes, enabling highly localised neuropeptide release and receptor activation on the level of individual cells.

### 2.8 LED Uncaging Activates and Internalises OTR in Acute Mouse Cortical Slices

**FIGURE 8:**
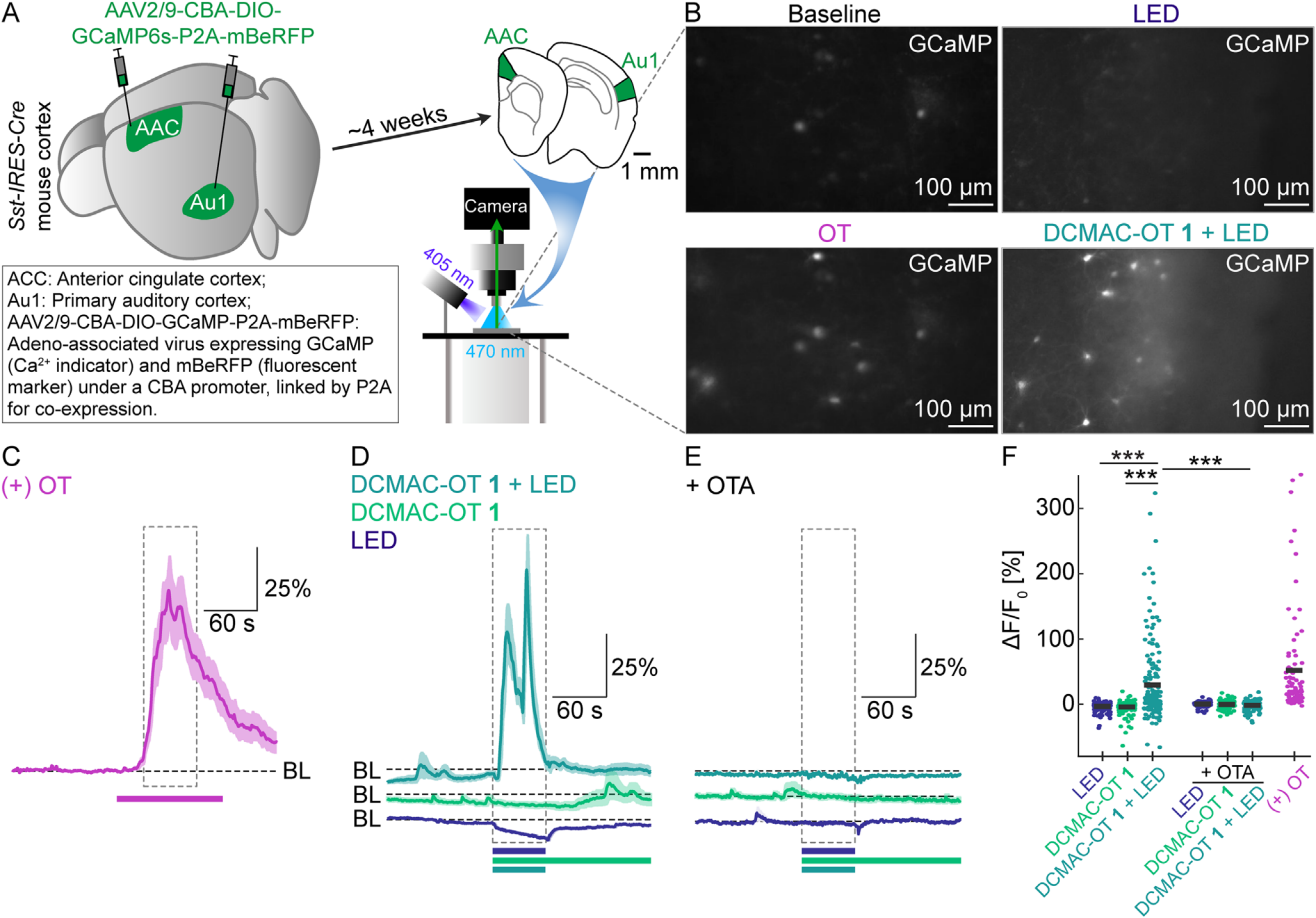
Ex vivo evaluation of DCMAC‐OT **1** via epifluorescence GCaMP imaging. (A) Schematic of cortical injection sites and epifluorescence GCaMP imaging on acute mouse cortical slices. (B) Example images of ex vivo epifluorescence GCaMP imaging. Upper left: baseline; lower left: 1 µM OT positive control; upper right: LED control, no GCaMP signal recorded during 60 s 405 nm LED without DCMAC‐OT **1**; bottom right: GCaMP signals during 60 s uncaging of 1 µM DCMAC‐OT **1** with 405 nm LED. (C–E) Normalised GCaMP fluorescence changes (Δ*F*/*F*_0_) are shown as solid lines with ± SEM as translucent areas: (C) Pink: 1 µM OT, *N* = 3 mice, *n* = 4 slices; (D) Blue: 405 nm LED control; green: 1–5 µM DCMAC‐OT **1** in the dark; turquoise: 1–5 µM DCMAC‐OT **1** and 60 s uncaging with 405 nm LED, *N* = 3 mice, *n* = 5 slices; (E) In the presence of 10 µM OTR antagonist (OTA: d(CH_2_)_5_[yITNCP(Orn)]‐COOH). Blue: 405 nm LED control; green: 1 µM DCMAC‐OT **1** in the dark; turquoise: 1 µM DCMAC‐OT **1** and 60 s uncaging with 405 nm LED, *N* = 2 mice, *n* = 4 slices. (F) Quantification of normalised GCaMP fluorescence changes (Δ*F*/*F*_0_) for all imaged cells during DCMAC‐OT and LED tests, as well as uncaging without or with OTA. Neither the LED nor the dark controls altered GCaMP signalling, whereas uncaging of DCMAC‐OT **1** induced changes in fluorescence comparable to those of the 1 µM OT positive control. The effects were significantly suppressed by the application of 10 µM OTA. Coloured bars indicate application times of the compounds or LED irradiation times; dashed lines indicate baseline fluorescence (BL); dashed boxes indicate time windows used for GCaMP Δ*F*/*F*_0_ quantification; ***p < 0.001.

To showcase the versatility and application scope of our probes and address the issue of non‐homogeneous concentrations across heterogeneous tissue, we investigated the most promising candidate—DCMAC‐OT **1**—in ex vivo acute brain slices from the mouse cortex. Validation of DCMAC‐OT uncaging efficiency in mammalian tissue is complicated by the diverse, region‐ and cell‐type‐specific endogenous expression of OTR. Therefore, we developed a cell‐type‐specific validation assay informed by recent literature demonstrating the functional expression of OTR in a subpopulation of GABAergic (γ‐aminobutyric acid) interneurones in the mouse cortex, which is partly marked by co‐expression of somatostatin (SST) [119, 120, 121, 122]. OTR primarily couples to G_q_ proteins, which typically drive elevations in intracellular Ca^2+^ [123]. Based on this predicted downstream signalling of OTR activation, we assessed the efficacy of DCMAC‐OT **1** uncaging in the mouse cortex by using SST‐specific expression of the genetically encoded green fluorescent calcium indicator GCaMP6s [124]. To this end, we injected an adeno‐associated viral vector (AAV2/9‐CBA‐DIO‐GcaMP‐P2A‐mBeRFP) into two cortical areas (primary auditory cortex: Au1; anterior cingulate cortex: ACC) of *Sst‐IRES‐Cre* (SST‐internal ribosome entry site‐Cre recombinase) mice (Figure 8A) for Cre‐dependent expression of GCaMP and mBeRFP (monomeric blue‐shifted red fluorescent protein). The CBA (cytomegalovirus enhancer/chicken β‐actin) promoter drives strong expression, and P2A (porcine teschovirus‐1 2A peptide) enables co‐expression of both proteins. Four weeks after injections, epifluorescence imaging was performed. Virus‐mediated expression of GCaMP6s was clearly visible in a sparse population of neurones in acute cortical slices, as expected for SST‐driven viral expression. Bath application of OT (1 µM) increased calcium‐dependent fluorescence in 95% of imaged neurones, validating the functionality of our assay (Δ*F*/*F*_0_: 51.7% ± 9.3%, *n* = 96 cells in 4 slices from 3 mice, Figure 8B,C). To investigate the performance of DCMAC derivatives on endogenous receptors, we bath‐applied 1–5 µM DCMAC‐OT and then illuminated it with a 405 nm LED (60 s, ∼10 mW). Uncaging induced responses in 67% of recorded neurones (*n* = 172 cells in 5 acute slices from 3 mice; ΔF/F_0_: 32% ± 2%; Figure 8B,D,F; Video S1). By contrast, illumination with a 405 nm LED alone, or bath application of 1–5 µM DCMAC‐OT **1** in the dark, elicited responses in only 0% and 8% of the recorded cells, respectively (Δ*F*/*F*_0_: −4.2% ± 0.3% and −1.0% ± 0.2%, respectively; Figure 8B,D,F; Video S1). When repeating the same uncaging experiment in the presence of 10 µM OTR antagonist (OTA: d(CH_2_)_5_[yITNCP(Orn)]‐COOH) [125], GCaMP signalling in response to light irradiation was significantly suppressed (Δ*F*/*F*_0_: −0.3% ± 0.3%, 4 slices from 2 mice; *p* < 0.001, Mann–Whitney *U* test; Figure 8E,F; Video S2), confirming that observed receptor activation was caused by release of OT through uncaging and not through the photocage or other side products. These findings demonstrated that uncaging of DCMAC‐OT **1** specifically and efficiently recruited endogenous OTRs in mouse cortical neurones, confirming the photoprobe's functionality ex vivo.

While all in vitro and ex vivo experiments were performed using caged OT compounds as a model system, the findings are expected to translate not only to the VP system but also to other similar neuropeptide systems, as the uncaging process solely depends on the type of chemical conjugation and the point of attachment. After irradiation, endogenous neuropeptides are released and trigger downstream intracellular signalling by interacting with their respective receptor(s). Although the RP‐HPLC uncaging experiments with DCANBP‐OT **3** revealed slower, non‐quantitative photocleavage under physiological conditions, the irradiation times used in the in vitro experiments were sufficient to generate a robust, reproducible pCREB signal in both the overexpression system and primary tissue cultures without causing adverse effects on the cells. During the 2PE experiments, DCANBP‐OT **3** was uncaged slightly faster than DCMAC‐OT **1**. Regardless, the rapid 1PE uncaging of the DCMAC derivatives (**1**/**2**), the re‐establishment of activity (Figure 5D–E), and the 2PE compatibility (Figure 7) distinguished the DCMAC PPGs. The versatility of the DCMAC PPG class was further highlighted by experiments utilising DCMAC‐OT **1** in more complex ex vivo systems (Figure 8). This supports the use of DCMAC derivatives as powerful photoprobes for studying neuropeptide signalling across various experimental settings, with high spatiotemporal resolution and no observed side effects.

## 3 Discussion

Neuropeptides are ancient, highly conserved, and critical signalling molecules that regulate a plethora of functions, yet their exact signalling pathways and related (patho)physiological implications remain elusive. While recent advances have been made in producing genetically engineered receptor‐based sensors to study endogenous neuropeptides in vivo [126, 127], it is equally important to advance molecular probe development that enables the precise activation of neuropeptide receptors with high spatiotemporal resolution without the need for prior genetic modifications [128]. Particularly for ex vivo and in vivo studies, where neuropeptide function is often highly dependent on concentration and tissue type, diffusion from the administration site can lead to receptor activation outside the area of interest, complicating the analysis of observed effects [28, 34]. The field of photopharmacology addresses this by using precise light activation to enable excellent spatiotemporal control [129, 130].

In this study, we investigated the synthesis, implementation, and performance of three state‐of‐the‐art PPGs based on coumarin, nitrobiphenylpropyl, and BODIPY cores. Our selection was based on rapid photocleavage and suitability for prospective ex vivo and in vivo experiments. Since the nitrobiphenylpropyl PPGs do not produce nitrosoaldehydes or ketones upon cleavage and the original, non‐cytotoxic, coumarin and BODIPY PPGs are regenerated after irradiation, biocompatibility concerns often associated with older PPGs are alleviated (Figure S2) [70, 85, 86, 99, 101]. Incorporation of the PPGs into model neuropeptides OT and VP allowed us to assess and directly compare their photocaging effects, revealing the outstanding features of the DCMAC and DCANBP PPGs **9** and **14**. Both photocages tolerated TFA cleavage and oxidative folding conditions, making them fully compatible with an on‐resin photocaging approach. Additionally, they exhibited favourable photochemical properties, dark stability and large (in)activity windows. By contrast, the BODIPY PPG **17** required a more careful in‐solution photocaging approach to circumvent decomposition of the BODIPY core under acidic conditions during TFA cleavage after SPPS (Scheme 3). Additionally, partial hydrolysis in the dark was observed with the BODIPY PPG (18% hydrolysis over 12 h). Removal of the PPGs from the peptides was achieved using either inexpensive LEDs or more sophisticated 2PE laser systems. During 1PE uncaging, the fastest rates were observed for DCMAC‐OT **1** (15 s) and Me_6_I_2_‐BODIPY‐OT **5** (30 s) under physiological conditions, while DCANBP‐OT **3** required longer irradiation times (∼94% uncaged after 3 min). The biocompatibility of the PPGs and photoprobes (**1**, **3**, **5**) was confirmed in cell viability assays, with no cytotoxic effects observed related to the PPGs, both before and after irradiation (Figure 4).

The DCANBP group (DCANBP‐OT/VP **3**/**4**) efficiently masked activity of OT and VP, with >2,100‐3,900‐fold higher EC_50_ values compared to the parent peptides (Figure 5A–C). 60 s uncaging resulted in good peptide release and receptor activity (Figure 5D,E; Figure S6B,E). The DCMAC group (DCMAC‐OT/VP **1**/**2**) also masked the activity well (150‐750‐fold higher EC_50_ values) and displayed rapid and near complete peptide release after 60 s irradiation (Figure 5D,E; Figure S6A,D). By contrast, the Me_6_I_2_‐BODIPY group (Me_6_I_2_‐BODIPY‐OT/VP **5**/**6**) masked bioactivity not as efficiently (3‐9‐fold higher EC_50_ values; Figure 5A–C) and had low peptide release and receptor activation upon uncaging (Figure 5D,E; Figure S6C,F). Considering these findings, the Me_6_I_2_‐BODIPY compounds (**5**/**6**) are less attractive photoprobes compared to the DCMAC (**1**/**2**) and DCANBP (**3**/**4**) derivatives, which excelled in terms of activity masking and peptide release kinetics.

The observed differences in (in)activity windows between the three similarly sized PPGs are likely due to the negatively charged carboxylic acid moieties of DCMAC and DCANBP interfering with receptor binding. By contrast, the more lipophilic Me_6_I_2_‐BODIPY derivatives could still activate the respective receptors, albeit at slightly higher concentrations. That the binding pocket of OTR and VPRs can accommodate such bulkier groups was recently demonstrated through OT and VP homo‐ and heterodimers, as well as other OT derivatives that have non‐charged PPGs at the N‐terminal α‐amine and still show residual activity, supporting our hypothesis of the importance of the negative charges in this position [67, 131]. If the BODIPY PPG is desired due to its higher wavelength 1PE uncaging (>500 nm), then we would recommend introducing negative charges to the core of similar sized BODIPYs (∼500 Da). While 2PE‐active BODIPYs are available, these PPGs still face biocompatibility issues, mainly due to their large extended π‐systems and resulting low solubility [132].

The application of our photoprobes (**1**, **3**, **5**) in OTR‐GFP stably overexpressing HEK‐293 cells worked well, with no receptor activation and internalisation observed in the dark (Figure 6A–C, Figure S9), but pronounced receptor activation and internalisation and increased pCREB signals upon light irradiation of DCMAC‐OT **1** and DCANBP‐OT **3** (**1**: 30/60 s at 365 nm; **3**: 30/60 s at 405 nm; **5**: 10/30 s at 365 nm). Me_6_I_2_‐BODIPY **5** did not perform as well, with no receptor internalisation observed. Hence, we did not further pursue the Me_6_I_2_‐BODIPY compounds for the more complex experiments.

DCMAC‐OT **1** and DCANBP‐OT **3** were inactive in the dark on primary rat hippocampal neurones natively expressing OTR, with pCREB levels comparable to the negative control aCSF (Figure 6D–F, Figures S10–S12). Irradiation for 60 s with LEDs (**1**: 365 nm; **3**: 405 nm) restored OT signalling to levels equal to those of the positive control (OT: 100 nM), confirming excellent temporal control over OT release and reproducibly generating quantifiable signals in primary tissue cultures. The LED application did not compromise any dendritic structural integrity, not even after 60 s at 365 nm and subsequent 1 h exposure to the uncaging mixture, confirming good compatibility of our DCMAC‐ and DCANBP photoprobes for neuronal settings with native receptor expression.

To explore the spatiotemporal resolution of our photoprobes, we conducted 2PE experiments using DCAMC‐OT **1** and DCANBP‐OT **3** in the same HEK‐293 hOTR‐GFP system. Since the likelihood of two photons intersecting and simultaneously transferring their energy is drastically reduced, this process frees only very little of the caged compound [39, 133]. As a result, 2PE uncaging proceeds less efficiently compared to 1PE uncaging but also allows the release of compounds and activation of receptors on a single cell level [47]. This aligned with our findings. Uncaging of both compounds, **1** (2 × 365 nm → 730 nm, 60 pulses, 30 ms, 0.3 Hz) and **3** (2 × 390 nm → 780 nm, 60 pulses, 45 ms, 0.25 Hz), resulted in receptor activation with extreme precision (Figure 7), making them attractive research tools for studying neuropeptide activity at the single dendrite level. For applications that require the release of larger quantities of peptides, particularly for in vivo studies, 1PE excitation might be the preferred strategy. Given the slower 1PE uncaging of DCANBP‐OT **3** under physiological conditions, we selected DCMAC‐OT **1** for the subsequent experiments. Its robust 2PE uncaging performance, combined with rapid, nearly quantitative 1PE cleavage, made DCMAC our overall preferred PPG class.

While we have demonstrated that the on‐demand release of OT from our caged compounds can activate OTR at native expression levels in primary neurones, ex vivo and in vivo investigations pose additional challenges. In particular, the brain's heterogeneous environment can lead to an uneven distribution of applied compounds, making it difficult to achieve precise local concentrations. Keeping in mind that neuropeptide function is highly dependent on the tissue type and the concentrations of available peptides, and that conventional ex vivo applications usually require large amounts, unspecific receptor activation and off‐target effects can impede the interpretation of these experiments. Our photocaged neuropeptides overcome these limitations by allowing high concentrations to be introduced in an inert form, saturating the tissue without triggering biological activity. Upon targeted light exposure, these compounds can be rapidly and precisely activated at defined times, ensuring controlled signalling while minimising diffusion‐related artefacts and off‐target interactions. This strategy provides both spatial and temporal resolution, enabling more accurate studies of neuropeptide function in complex tissue environments. Our most promising photoprobe, DCMAC‐OT **1**, performed extremely well in such a setting. Despite using higher concentrations than in cell culture experiments (1‐5 µM), significant receptor activation was not observed without 405 nm LED illumination. In contrast, irradiation with our 405 nm LED produced almost instantaneous effects, demonstrating our ability to manipulate peptide release temporally (Figure 8D; Video S1). These effects were time‐limited, indicating that sufficient OT was released to activate a majority of imaged neurones and subsequently trigger the internalisation of activated OTR in the investigated area (∼0.3 mm^2^). Therefore, the uncaging process of **1** is fast enough to provide sufficient amounts of OT to allow investigations on all biologically relevant time scales (receptor activation: ms–s; receptor internalisation: s–min, long‐term signalling: min–h) [127]. Since the released OT is unmodified, subsequent signalling and processing of the neuropeptide closely mimic endogenous neuropeptide release in real biological systems. These effects were blocked by co‐administration of a selective OTR antagonist, confirming that GPCR activation was indeed mediated through uncaged OT (Figure 8E; Video S2). The 60 s, 405 nm LED light irradiation, furthermore, did not elicit any significant effects on neuronal calcium signalling. Although using our low‐cost configuration released OT across the entire slice, the integration of higher‐powered LEDs with fibre‐optic systems or laser technology is anticipated to enable even more spatially restricted neuropeptide release, as demonstrated earlier in our 2PE uncaging experiments.

## 4 Conclusions

We synthesised and compared three diverse types of photocages (DCMAC, DCANBP, Me_6_I_2_‐BODIPY) and evaluated their application scope as biocompatible neuropeptide photoprobes. DCMAC and DCANBP were the best‐performing photocages that can conveniently be incorporated into peptides via on‐resin chemistry, resulting in excellent masking/photocaging of bioactivity, high stability, and compatibility with oxidative peptide folding and one‐ and two‐photon activation protocols (DCMAC: 1PE: 365–405 nm, 2PE: 730 nm; DCANBP: 1PE: 405 nm, 2PE: 780 nm). By contrast, the Me_6_I_2_‐BODIPY photocage (1PE: 527 nm) performed poorly, with synthetic limitations, poor bioactivity masking, and partial degradation and activity in the dark. The use of the DCMAC‐OT **1** and DCANBP‐OT **3** photoprobes in cellular imaging assays and on primary neurones highlighted their application scope in state‐of‐the‐art neuroscience experiments, enabling precise receptor activation with our optimised rapid uncaging protocols and easy‐to‐implement LED setups, without damaging cells or neurones. In live‐cell two‐photon microscopy experiments, highly localised receptor internalisation was demonstrated through 2PE irradiation of both DCMAC‐OT **1** and DCANBP‐OT **3**, highlighting the powerful spatiotemporal control of receptor activation with light. Ultimately, the considerable inactivity window; the sufficiently fast 2PE cleavage, and, most importantly, the extremely fast 1PE cleavage of DCMAC‐OT **1**, both in vitro and ex vivo, made the DCMAC PPG our favourite photocage in this study. In summary, this work compared three state‐of‐the‐art PPGs and introduced new synthetic strategies for creating biocompatible neuropeptide photoprobes, thereby expanding our photopharmacological toolbox for studying neuropeptide signalling. Controlled neuropeptide release with high spatiotemporal precision resulted in downstream signalling even at native receptor expression levels, while preserving neuronal integrity. The photocages and developed protocols also hold broad applicability for other peptides, thereby enabling more sophisticated, precise, and meaningful experiments in neuroscience.

## 5 Experimental

Detailed synthetic procedures, as well as spectroscopic and spectrometric characterisations, are enclosed in the Supporting Information (SI).

## Conflicts of Interest

The authors declare no conflicts of interest.

## Supporting information




**Supporting File 1**: anie71997‐sup‐0001‐SuppMat.pdf.

**Supporting File 2**: anie71997‐sup‐0002‐SupMat.avi.

**Supporting File 3**: anie71997‐sup‐0003‐SupMat.avi.
The authors have cited additional references within the Supporting Information [57, 101, 105, 107, 109, 110, 111, 112, 115, 134, 135, 136].

## Data Availability

The data that support the findings of this study are available in the Supporting Information of this article.
