# Supplementary material for: Photocaged Oxytocin and Vasopressin Probes to Decipher Neuropeptide Signalling With High Spatiotemporal Resolution
Source: Angew Chem Int Ed Engl. 2026 Apr 13;65(22):e13373. doi: 10.1002/anie.202513373 (PMC13206411; doi:10.1002/anie.202513373)
Supplement: Supplementary file 1 — Supporting File 1: anie71997‐sup‐0001‐SuppMat.pdf. [file ANIE-65-e13373-s002.pdf]

Supporting Information

Photocaged Oxytocin and Vasopressin Probes to Decipher Neuropeptide Signalling with High Spatiotemporal Resolution

Konstantin Raabe,[1],[2] Predrag Kalaba,[1] Xuan Ling Hilary Yong,[3] Greta Crudeli,[4] Sarah Melzer,[4] Erik Keimpema,[5] Victor Anggono,[3],[6] Markus Muttenthaler*[1],[7]

[1] K. Raabe, P. Kalaba, M. Muttenthaler
Faculty of Chemistry, Institute of Biological Chemistry, University of Vienna, Vienna, Austria
E-mail: markus.muttenthaler@univie.ac.at

[2] K. Raabe
Vienna Doctoral School in Chemistry, University of Vienna, Vienna, Austria

[3] X. L. H. Yong, V. Anggono
Faculty of Health, Medicine and Behavioural Sciences, Queensland Brain Institute, Clem Jones Centre for Ageing Dementia Research, The University of Queensland, Brisbane, Australia.

[4] G. Crudeli, S. Melzer
Department of Neuronal Cell Biology, Center for Brain Research, Medical University of Vienna, Vienna, Austria

[5] V. Anggono
NHMRC Centre for Research Excellence in Mechanisms in NeuroDegeneration – Alzheimer's Disease (MIND-AD CRE), Brisbane, Australia

[6] E. Keimpema
Department of Molecular Neurosciences, Center for Brain Research Medical University of Vienna, Vienna, Austria

[7] M. Muttenthaler
Institute for Molecular Bioscience, The University of Queensland, Brisbane, Australia
E-mail: m.muttenthaler@uq.edu.au

Table of Contents

Abbreviations 2

Experimental 3

Caging rational 19

Uncaging mechanism 19

Uncaging in 30%ACN/ddH_2O 20

Dark stability 20

BODIPY-amine 21

IP1 assays 21

Confocal Microscopy 23

NMR spectra of compounds 7-17a 29

Analytical RP-HPLC 1-6 43

High-resolution mass spectra of compounds 1-17 46

SI References 54

Abbreviations

1PE	one-photon excitation
2PE	two-photon excitation
ACC	anterior cingulate cortex
ACN	acetonitrile
AcOH	acetic acid
aCSF	artificial cerebrospinal fluid
ANBP	amino nitrobiphenylpropyl
aq.	Aqueous
Au1	primary auditory cortex
BL	base line
Boc	*tert*-butyloxycarbonyl
BODIPY	boron-dipyrromethene
BSA	bovine serum albumin
cAMP	cyclic adenosine monophosphate
CBA	(cytomegalovirus enhancer / chicken β-actin
CREB	cAMP response element binding protein
$CDCl_3$	deuterated chloroform
d	doublet (NMR)
DAPI	4′,6-diamidino-2-phenylindole
DCANBP	dicarboxy amino nitrobiphenylpropyl
DCM	dichloromethane
DCMAC	dicarboxymethyl aminomethyl coumarin
dd	doublet of doublet (NMR)
ddH_2O	ultrapure water (Milli-Q)
DIBAL-H	diisobutylaluminiumhydride
DIPEA	N, N-ethyldiisopropylamine
DMEM	Dulbecco's Modified Eagle Medium
DMF	N, N-dimethylformamide
DMSO	dimethyl sulfoxide
EDTA	ethylenediaminetetraacetic acid
eq	equivalents
ESI^+	positive electrospray ionisation
EtOAc	ethyl acetate
EtOH	ethanol
Et_2O	diethyl ether
FBS	fetal bovine serum
Fmoc	fluorenylmethyloxycarbonyl
FRET	Förster resonance energy transfer
GABA	γ-aminobutyric acid
GCaMP	genetically encoded green fluorescent calcium indicator
GFP	green fluorescent protein
GPCR	G protein-coupled receptor
HATU	1-[bis(dimethylamino)methylene]-1*H*-1,2,3-triazolo[4,5-*b*]pyridinium 3-oxide hexafluorophosphate
HEK-293	human embryonic kidney (cells)
hOTR	human oxytocin receptor
HPLC	high-performance liquid chromatography
HRMS	high-resolution mass spectrometry
IP_1	D-myo-inositol 1-monophosphate
IP_2	D-myo-inositol 4,5-bisphosphate
IP_3	D-myo-inositol 1,4,5-trisphosphate
IRES	internal ribosome entry site
J	coupling constant (NMR)
LC	liquid chromatography
LED	light-emitting diode
m	multiplet (NMR)
mBeRFP	monomeric blue-shifted red fluorescent protein
MAP2	microtubule-associated protein 2
MeI	iodomethane
MeOH	methanol
MPLC	medium-pressure liquid chromatography
MS	mass spectrometry
MTT	3-(4,5-di methyl thiazol-2-yl)-2,5-diphenyltetrazolium bromide
MW	molecular weight
NMR	nuclear magnetic resonance
NIR	near-infrared
NIS	N-iodosuccinimide
NPC	4-nitrophenyl chloroformate
NPY	neuropeptide Y
NVOC	6-nitroveratryloxycarbonyl
o-NB	*ortho*-nitro benzyl
on	overnight
OT	oxytocin
OTA	oxytocin antagonist
P2A	Porcine teschovirus-1 2A peptide
PBS	phosphate-buffered saline
PFA	paraformaldehyde
PPG	photolabile protecting group
ppm	parts per million
R_f	retention factor
ROI	region of interest
RP	reversed phase
RT	room temperature (25 °C)
s	singlet (NMR)
sat.	saturated
SEM	standard error of the mean
SPPS	solid-phase peptide synthesis
SSp	Staurosporine
SST	somatostatin
TFA	trifluoroacetic acid
TIPS	triisopropylsilane
THF	tetrahydrofuran
TLC	thin-layer chromatography
UV	ultraviolet
Vis	visible
VP	vasopressin
hV_xR	human vasopressin receptor x

Experimental

General Conditions

All commercial chemicals were obtained from reputable suppliers, including Sigma-Aldrich (Merck), Alfa Aesar, Tokyo Chemical Industry, Thermo Fisher Scientific, and Fluorochem, and used without further purification. Deuterated solvents for NMR spectroscopy were purchased from Eurisotop and Sigma-Aldrich. Solvents for column chromatography were purchased from Thermo Fisher Scientific in analytical grade (purity >99.8%). If not specifically stated otherwise, reagents for cell culture were purchased from Sigma-Aldrich and Thermo Fisher Scientific. For all aqueous solutions and buffer preparations, ultrapure water (ddH_2O; Milli-Q) was used. To minimise photobleaching and premature photocleavage, all reactions were carried out in the dark in amber glassware. All non-aqueous reactions were performed under inert conditions with commercially available dry solvents and 3 Å molecular sieves. Glassware and 3 Å molecular sieves were dried in an oven for 16 h at 135 °C and subsequently dried with a heat gun, using the Schlenk technique. Gravity column chromatography was performed using a 100-200 mesh silica gel. Medium-pressure liquid chromatography (MPLC) purification (flash chromatography) was performed with a CombiFlash NextGen 300+ System from Teledyne, with a 200-800 nm UV/Vis detector and prepacked RediSep columns. Yields of caged peptides were calculated from the theoretical yield of 0.05 mmol scale. Solvents for synthesis purposes were used at guaranteed pure reagent (GPR) grade. Analytical thin-layer chromatography (TLC) was performed using Merck Kieselgel 60 F254 silica gel plates or Polygram Alox N/UV254 aluminium oxide plates. Visualisation was by UV light (254/365 nm). NMR spectra were recorded on Bruker AV III spectrometers, operating at 400, 600, and 700 MHz for ^{1}H-NMR and 101, 151, and 176 MHz for ^{13}C-NMR, and Bruker AV NEO spectrometers operating at 500 MHz for ^{1}H-NMR and 125 MHz for ^{13}C-NMR. Shifts were referenced to the internal solvent signals. NMR data were processed using MestReNova 14. The peptide purity of caged OT and VP derivatives (final analysis) was determined by UV absorbance at 214 nm and peak integration using a Dionex Ultimate 3000 HPLC system. 50 µM stock solutions of the final compounds **1**-**6** in 30% ACN were prepared for this analysis. 50 µL of the solution was injected onto a Kromasil 300-5-C_{18} HPLC column (4.6 x 150 mm, 5 µm, 300 Å; 5-95% solvent B (A: 0.1% TFA in ddH_2O; B: 0.08% TFA in ACN) in 70 min, 1 mL/min, 30 °C). Data were collected, and the peak areas were manually integrated using Chromeleon 7. HR-ESI-MS spectra were acquired in positive ion mode using an ESI-Qq-TOF mass spectrometer - maXis UHRTOF (Bruker Daltonik GmbH, Bremen, Germany) under the following conditions: Capillary voltage: 4500 V, dry gas flow: 4.0 L/min (nitrogen), dry temperature: 180 °C, mass range: 50-1,900 m/z, sample introduction *via* direct infusion in ACN/MeOH +1% H_2O. Flow rate: 3 µL/min. Software: Bruker Compass 1.5 for OTOF series - otofControl Version 3.2, DataAnalysis Version 4.1. Solvent A: 0.1% formic acid in ddH_2O; Solvent B: 0.08% formic acid in ACN. All final compounds (**1**-**6**) were >95% pure, determined by analytical HPLC at 214 nm.

Synthesis

Photolabile Protecting Groups (PPGs)

Coumarin Derivatives

Di-*tert*-butyl 2,2'-((4-methyl-2-oxo-2H-chromen-7-yl)azanediyl)diacetate (*tBu₂-DCMAC*) 7

*t*Bu$_2$-DCMAC **7** was synthesised as reported previously.[1] 7-Amino-4-methyl coumarin (1 eq., 2.5 g, 14.27 mmol) and hydroquinone (0.15 eq., 250 mg, 2.28 mmol) were placed in a Schlenk flask. DIPEA (3 eq., 7.3 mL, 42.81 mmol) and *tert*-butyl bromoacetate (3.5 eq., 7.9 mL, 49.95 mmol) were added *via* a syringe, and the reaction mixture was stirred at 80 °C in the dark for 5 days. Once TLC analysis indicated the complete consumption of 7-amino-4-methyl coumarin, the reaction was allowed to cool to room temperature (RT, 25 °C), and the brown slurry was dissolved in DCM and transferred into a separating funnel. The organic layer was washed three times with water and brine, dried over anhydrous $MgSO_4$, and filtered. The solvent was removed *in vacuo,* and the crude product was purified *via* silica column chromatography (dry loading, 180 g SiO_2, PE/EtOAc=15→50%), yielding 2.1 g (5.26 mmol) of *t*Bu$_2$-DCMAC **7** as a yellow solid.

Yield: 37%. ^{1}H-NMR (400 MHz, $CDCl_3$): *δ*=7.42 (d, *J*=8.9 Hz, 1H, Ar-H), 6.52 (dd, 1H, Ar-H), 6.44 (d, *J*=2.6 Hz, 1H, Ar-H), 6.02 (d, *J*=1.0 Hz, 1H, Ar-H), 4.05 (s, 4H, -CH_2-), 2.34 (d, *J*=0.9 Hz, 3H, -CH_3), 1.48 (s, 18H, *tert*-butyl -CH_3) ppm. ^{13}C-NMR (101 MHz, $CDCl_3$): *δ*=169.16, 161.96, 155.62, 152.79, 151.24, 125.72, 111.36, 110.55, 109.07, 99.23, 82.57, 54.51, 28.22, 18.60 ppm. HRMS (ESI$^+$) *m/z:* exact mass: 403.1995 Da, observed: 404.2066 Da [M+H]$^+$, calculated: 404.2068 Da [M+H]$^+$, error: 0.2 mDa.

Di-*tert*-butyl 2,2'-((4-formyl-2-oxo-2H-chromen-7-yl)azanediyl)diacetate (*tBu₂-DCMAC-CHO*) 8

*t*Bu$_2$-DCMAC-CHO **8** was synthesised as reported previously.[1] *t*Bu$_2$-DCMAC **7** (1 eq., 2 g, 4.96 mmol) was placed into a Schlenk flask and dissolved in 40 mL xylene. SeO_2 (2 eq., 1.1 g, 9.91 mmol) was added, and the resulting yellow solution was heated to 140 °C and stirred under TLC control for 27 h. Once TLC analysis (PE/EtOAc=1/1) indicated full consumption of the starting material, black insoluble tars were removed from the dark yellow/brownish suspension *via* hot filtration, and xylene was removed *in vacuo.* The crude material was purified *via* column chromatography (100 g silica, dry loading, PE/EtOAc=33→50%), yielding 1.6 g (3.82 mmol) of the desired aldehyde compound *t*Bu$_2$-DCMAC-CHO **8** as a yellow solid.

Yield: 77%. ^{1}H-NMR (400 MHz, $CDCl_3$): *δ*=10.03 (s, 1H, -CHO), 8.37 (d, *J*=9.1 Hz, 1H, Ar-H), 6.58 – 6.53 (m, 2H, Ar-H), 6.48 (d, *J*=2.6 Hz, 1H, Ar-H), 4.06 (s, 4H, -CH_2-), 1.48 (s, 18H, *tert*-butyl -CH_3) ppm. ^{13}C-NMR (101 MHz, $CDCl_3$): *δ*=192.29, 168.81, 161.52, 156.85, 151.82, 143.88, 127.38, 119.93, 110.11, 105.85, 99.13, 82.78, 54.40, 28.20 ppm. HRMS (ESI$^+$) *m/z:* exact mass: 417.1788 Da, observed: 450.2093 Da [M+H+MeOH]$^+$, calculated: 450.2122 Da [M+H+MeOH]$^+$, error: 2.9 mDa.

Di-*tert*-butyl 2,2'-((4-(hydroxymethyl)-2-oxo-2H-chromen-7-yl)azanediyl)diacetate (*tBu₂-DCMAC-OH*) 9

*t*Bu$_2$-DCMAC-OH **9** was synthesised according to a literature procedure.[2] *t*Bu$_2$-DCMAC-CHO **8** (1 eq., 639.9 mg, 1.53 mmol) was placed into a round-bottom flask and dissolved in 30 mL MeOH. The dark orange solution was stirred at 0 °C, and $NaBH_4$ (96% pure; 1.75 eq., 100.9 mg, 2.67 mmol) was added slowly in small portions. After 3 h, the reaction was quenched by dropwise addition of 0.5 M HCl until the pH reached 2 and then neutralised with a saturated aqueous $NaHCO_3$ solution. The reaction mixture was transferred into a separating funnel and extracted with EtOAc (TLC control). The combined organic layers were washed twice with brine, dried over anhydrous Na_2SO_4, filtered,

and the solvent was removed *in vacuo*. The crude product was purified *via* column chromatography (55 g AlOx, wet loading, DCM/MeOH=5%), and 0.40 g (0.96 mmol) of the desired target compound *t*Bu_2-DCMAC-OH **9** was obtained as a yellow solid.

Yield: 63%. ^{1}H-NMR (400 MHz, $CDCl_3$): *δ*=7.32 (d, *J*=8.8 Hz, 1H, Ar-H), 6.49 (dd, *J*=8.8, 2.6 Hz, 1H, Ar-H), 6.46 (d, *J*=2.6 Hz, 1H, Ar-H), 6.34 (s, 1H, Ar-H), 4.81 (s, 2H, -CH_2-OH), 4.05 (s, 4H, -CH_2-), 1.48 (s, 18H, *tert*-butyl -CH_3) ppm. ^{13}C-NMR (101 MHz, $CDCl_3$): *δ*=169.14, 162.06, 155.75, 154.19, 151.24, 124.51, 109.19, 107.40, 99.38, 82.69, 77.48, 77.16, 76.84, 61.06, 54.47, 29.85, 28.23 ppm. HRMS (ESI$^+$) *m/z:* exact mass: 419.1944 Da, observed: 420.2915 Da [M+H]$^+$, calculated: 420.2017 Da, error: 0.4 mDa.

Di-*tert*-butyl 2,2'-((4-((((4-nitrophenoxy)carbonyl)oxy)methyl)-2-oxo-2H-chromen-7-yl)azanediyl)diacetate 9a

NPC (2.5 eq., 300 mg, 1.49 mmol) was dissolved in 2 mL DCM and pyridine 4.65 equivalents, 223 µL, 2.77 mmol) was added at 0 °C. The resulting suspension was stirred, and *t*Bu_2-DCMAC-OH **9** (1 eq., 250 mg, 0.596 mmol) in 2 mL DCM was added dropwise. The reaction was kept at 0 °C and stirred under TLC monitoring for 1.5 h. As TLC analysis indicated full consumption of the starting material, the reaction was purified *via* column chromatography (silica, wet loading, PE/EtOAc=20%) and 283 mg (0.484 mmol) of compound **9a** was obtained as a yellow solid.

Yield: 81%. ^{1}H-NMR (600 MHz, $CDCl_3$): *δ*=8.30 (dd, *J*=9.2, 1.0 Hz, 2H, Ar-H), 7.42 (d, *J*=9.1 Hz, 2H, Ar-H), 7.36 (d, *J*=8.9 Hz, 1H, Ar-H), 6.55 (dd, *J*=8.9, 2.6 Hz, 1H, Ar-H), 6.49 (d, *J*=2.5 Hz, 1H, Ar-H), 6.31 (t, *J*=1.2 Hz, 1H, Ar-H), 5.40 (d, *J*=1.3 Hz, 2H, -CH_2-O-), 4.06 (s, 4H, -CH_2-), 1.48 (s, 18H, tert-butyl -CH_3) ppm. ^{13}C-NMR (151 MHz, $CDCl_3$): *δ*=168.91, 161.35, 155.92, 155.31, 152.23, 151.66, 147.74, 145.79, 126.23, 126.21, 125.56, 124.56, 121.86, 115.76, 109.51, 108.95, 108.94, 107.86, 99.52, 82.81, 65.74, 54.46, 28.21 ppm. HRMS (ESI$^+$) *m/z:* exact mass: 584.2006 Da, observed: 607.1901 Da [M+Na]$^+$, calculated: 607.1898 Da, error: 0.3 mDa.

***o*-Nitrophenethyl Derivatives**

***tert*-Butyl 2-(5-bromo-2-nitrophenyl) acetate 10**

tert-Butyl-2-(5-bromo-2-nitrophenyl) acetate **10** was synthesised using a modified literature procedure.[3] *t*BuOK (6.2 eq., 17.22 g, 153.46 mmol) was placed into a Schlenk flask, dissolved in 50 mL DMF and stirred at 0 °C. A solution of 4-bromonitrobenzene (1 eq., 5 g, 24.75 mmol) and *tert*-butyl chloroacetate (1.5 eq., 5.31 mL, 37.13 mmol) in 50 mL DMF was added dropwise to the cooled *t*BuOK solutions *via* a syringe, resulting in an instant colour change to dark blue. The ice bath was removed, and the reaction was stirred at RT under TLC (PE/EtOAc=19/1) monitoring. After 2 h, the reaction mixture was again cooled to 0 °C and quenched by the addition of 1 N HCl (pH=2-3), leading to the formation of an off-white precipitate. The reaction mixture was transferred into a separating funnel and extracted with EtOAc. The combined organic layers were washed with brine and dried over anhydrous Na_2SO_4, filtered, and concentrated *in vacuo*. The dark brown crude product was purified *via* MPLC (silica 300 g, wet loading, PE/EtOAc=0→50%), yielding 6.6 g (20.8 mmol) of compound **10** as yellow crystals.

Yield: 84%. ^{1}H-NMR (600 MHz, $CDCl_3$): *δ*=7.98 (d, *J*=8.7 Hz, 1H, Ar-H), 7.59 (dd, *J*=8.7, 2.1 Hz, 1H, Ar-H), 7.50 (d, *J*=2.1 Hz, 1H, Ar-H), 3.91 (s, 2H, -CH_2-), 1.44 (s, 9H, *tert*-butyl -CH_3) ppm. ^{13}C-NMR (151 MHz, $CDCl_3$): *δ*=168.63, 147.87, 136.31, 132.52, 131.67, 128.35, 126.81, 82.38, 40.95, 28.10 ppm. HRMS (ESI$^+$) *m/z:* exact mass: 315.0106 Da, observed: 337.9998 Da [M+Na]$^+$, calculated: 337.9998 Da [M+Na]$^+$, error: 0.0 mDa.

***tert*-Butyl 2-(5-bromo-2-nitrophenyl) propanoate 11**

tert-Butyl 2-(5-bromo-2-nitrophenyl)propanoate **11** was synthesised using a literature procedure.[3] NaH (60% pure in paraffin oil; 1.01 eq., 357.1 mg, 8.93 mmol) was placed into a Schlenk flask and washed with 5 mL n-heptane to

remove the paraffin oil. After removing the n-heptane, the NaH was suspended in 15 mL THF and cooled to -35 °C *via* an isopropanol/liquid N_2 bath. The starting material, *tert*-butyl-2-(5-bromo-2-nitrophenyl) acetate **10**, was placed into a Schlenk flask, dissolved in 10 mL of THF, and added to the suspension dropwise *via* a syringe. Afterwards, MeI (2 eq., 1.10 mL, 17.15 mmol) was added dropwise *via* a syringe. The reaction was stirred at -35 °C for 1 h before the cooling bath was removed, and stirring was continued for 24 h at RT. The next morning, the reaction was cooled to 0 °C and quenched with ddH_2O. The reaction mixture was extracted with EtOAc, and the combined organic phases were dried over anhydrous Na_2SO_4, filtered, and dried *in vacuo*. After purification *via* column chromatography (100 g silica, wet loaded, PE/EtOAc=5→10%), 2.1 g (6.32 mmol) of the desired compound **11** was obtained as a yellow solid.

Yield: 72%. ^{1}H-NMR (400 MHz, $CDCl_3$): δ=7.82 (d, *J*=8.7 Hz, 1H, Ar-H), 7.62 (d, *J*=2.1 Hz, 1H, Ar-H), 7.54 (dd, *J*=8.7, 2.1 Hz, 1H, Ar-H), 4.21 (q, *J*=7.2 Hz, 1H, -CH-), 1.57 (d, *J*=7.2 Hz, 3H, -CH_3), 1.40 (s, 9H, *tert*-butyl -CH_3) ppm. ^{13}C-NMR (101 MHz, $CDCl_3$): *δ*=171.81, 148.05, 137.83, 132.97, 131.13, 128.13, 126.45, 81.99, 42.38, 27.97, 17.52 ppm. HRMS (ESI$^+$) *m/z:* exact mass: 329.0263 Da, observed: 352.0153 Da [M+Na]$^+$, calculated: 352.0153 Da [M+Na]$^+$, error: 0.2 mDa.

2-(5-Bromo-2-nitrophenyl)propan-1-ol 12

2-(5-Bromo-2-nitrophenyl)propan-1-ol **12** was synthesised according to a literature procedure.[3] *tert*-Butyl 2-(5-bromo-2-nitrophenyl) propanoate **11** (1 eq., 2.0811 g, 6.3 mmol) was placed into a Schlenk flask and dissolved in 80 mL THF. The solution was cooled with an ice bath, and DIBAL-H (1 M in THF, 19 mL, 19 mmol) was added. TLC analysis (PE/EtOAc=4/1) after 3 h indicated full consumption of the starting material, and the reaction was quenched with 100 mL of 8 N HCl. The reaction mixture was transferred into a separating funnel and extracted with EtOAc (TLC control). The combined organic layers were washed with brine, dried over anhydrous Na_2SO_4, and concentrated *in vacuo*. The crude product was purified *via* column chromatography (100 g silica, wet loaded, PE/EtOAc=10→20%), and 0.87 g (3.77 mmol, 59%) of the desired compound **12** was obtained as a red oil.

Yield: 59%. ^{1}H-NMR (400 MHz, $CDCl_3$): *δ*=7.65 (d, *J*=8.6 Hz, 1H, Ar-H), 7.63 (d, *J*=2.0 Hz, 1H, Ar-H), 7.49 (dd, *J*=8.6, 2.1 Hz, 1H, Ar-H), 3.87 – 3.70 (m, 2H, -CH_2-), 3.61 – 3.50 (m, 1H, -CH-), 1.33 (d, *J*=6.9 Hz, 3H, -CH_3) ppm. ^{13}C-NMR (101 MHz, $CDCl_3$): *δ*=149.54, 140.69, 131.76, 130.59, 127.66, 125.89, 67.71, 36.48, 17.51 ppm. HRMS (ESI$^+$) *m/z:* exact mass: 258.9844 Da, observed: 281.9737 Da [M+Na]$^+$, calculated: 281.9736 Da [M+Na]$^+$, error: 0.1 mDa.

2-(4'-Amino-4-nitro[1,1'-biphenyl]-3-yl)propan-1-ol (*ANBP-OH*) 13

2-(4'-Amino-4-nitro[1,1'-biphenyl]-3-yl)propan-1-ol (ANBP-OH) **13** was synthesised according to a literature procedure.[3] The reaction was carried out twice, limited by the volume of the microwave reaction vessel. 2-(5-Bromo-2-nitrophenyl)propan-1-ol **12** (1 eq., 487.5 mg, 1.87 mmol) 4-aminophelnyboronic acid•HCl (1.2 eq., 390.03 mg, 2.25 mmol), K_2CO_3 (2.7 eq., 699.43 mg, 5.06 mmol), Bu_4NBr (1 eq., 604.25 mg, 1.87 mmol) and $Pd(OAc)_2$ (catalytic; 1 spatula tip) were placed into a 20 mL microwave vial and 5 mL EtOH/ddH_2O (2/1, degassed) was added. The vial was flushed with argon, sealed and placed into a Biotage microwave reactor (settings: 30 s pre-stirring, 150 °C, 10 min). After the reaction vessels had cooled to RT, the seal was removed, and the two reactions were combined in a separating funnel for the subsequent workup. The reaction mixture was diluted with 20 mL ddH_2O and extracted with EtOAc. The combined organic layers were washed with brine, dried over anhydrous Na_2SO_4, filtered and concentrated *in vacuo*. The crude product was purified *via* column chromatography (50 g silica, wet loading, PE/EtOAc=33→50%), yielding 0.96 g (3.53 mmol) of the desired compound **13** as a brown oil.

Yield: 94%. ^{1}H-NMR (400 MHz, $CDCl_3$): δ=7.85 (d, J=8.5 Hz, 1H, Ar-H), 7.59 (d, J=1.8 Hz, 1H, Ar-H), 7.48 (dd, J=8.5, 1.9 Hz, 1H, Ar-H), 7.42 (d, J=8.5 Hz, 2H, Ar-H), 6.77 (d, J=8.5 Hz, 2H, Ar-H), 3.85 (d, J=8.4 Hz, 5H, overlay -Ar-NH_2, -CH_2-, -OH), 3.67 (h, J=6.8 Hz, 1H, -CH-), 1.38 (d, J=6.9 Hz, 3H, -CH_3) ppm. ^{13}C-NMR (101 MHz, $CDCl_3$): δ=148.59, 147.38, 146.02, 139.14, 129.08, 128.54, 127.43, 125.82, 125.30, 124.87, 115.49, 68.20, 36.61, 17.75 ppm. HRMS (ESI$^+$) *m/z:* exact mass: 272.1161 Da, observed: 273.1248 Da [M+H]$^+$, calculated: 273.1234 Da [M+H]$^+$, error: 1.4 mDa.

Di-*tert*-butyl 2,2'-((3'-(1-hydroxypropan-2-yl)-4'-nitro[1,1'-biphenyl]-4-yl)azanediyl)diacetate (*tBu₂-DCANBP-OH*) 14

*t*Bu$_2$-DCANBP-OH **14** was synthesised according to a literature procedure.[3] ANBP-OH **13** (1 eq., 961.50 mg, 3.53 mmol) dissolved in 40 mL DMF was placed in a Schlenk flask. *tert*-Butyl bromoacetate (2.5 eq., 1.3 mL, 8.83 mmol) and DIPEA (3 eq., 1.85 mL, 10.59 mmol) were added *via* a syringe, and the clear brown/yellow reaction mixture was stirred at 90 °C under TLC monitoring. TLC analysis (PE/EtOAc=1/1) after 16 h showed complete consumption of the starting material, and the reaction was cooled to RT and quenched by slow addition of saturated aqueous $NaHCO_3$ solution, resulting in the formation of a precipitate. The suspension was transferred into a separating funnel and extracted with EtOAc. The combined organic layers were washed with brine, dried over anhydrous Na_2SO_4, filtered, and concentrated *in vacuo*. The crude product was purified *via* column chromatography (150 g silica, wet loading, PE/EtOAc=10→50%), yielding 0.75 g (1.5 mmol) of the desired compound **14** as a red/brown stringy solid.

Yield: 43%. ^{1}H-NMR (400 MHz, $CDCl_3$): δ=7.84 (d, J=8.5 Hz, 1H, Ar-H), 7.59 (d, J=1.9 Hz, 1H, Ar-H), 7.49 (d, J=8.9 Hz, 2H, Ar-H), 7.49 – 7.46 (m, 1H, Ar-H), 6.67 (d, J=8.9 Hz, 2H, Ar-H), 4.06 (s, 4H, -CH_2-), 3.83 (d, J=6.5 Hz, 2H, -CH_2-), 3.67 (h, J=6.7 Hz, 1H, -CH-), 1.48 (s, 18H, *tert*-butyl -CH_3), 1.37 (d, J=6.9 Hz, 3H, -CH_3) ppm. ^{13}C-NMR (101 MHz, $CDCl_3$): δ=169.89, 148.69, 148.49, 145.89, 139.14, 128.38, 128.24, 125.78, 125.32, 124.79, 112.69, 82.16, 68.40, 68.22, 68.19, 60.54, 54.62, 36.60, 28.24, 21.19, 19.00, 17.76, 14.33 ppm. HRMS (ESI$^+$) *m/z:* exact mass: 500.2523 Da, observed: 523.2398 Da [M+Na]$^+$, calculated: 523.2415 Da [M+Na]$^+$, error: 1.7 mDa.

Di-*tert*-butyl 2,2'-((4'-nitro3'-(1-(((4-nitrophenoxy)carbonyl)oxy)propan-2-yl)-[1,1'-biphenyl]-4-yl)azanediyl)diacetate 14a

NPC (2.5 eq., 252 mg, 1.25 mmol) was dissolved in 2 mL DCM and pyridine 4.65 equivalents,187 µL, 2.32 mmol) was added at 0 °C. The resulting suspension was stirred, and *t*Bu$_2$-DCANBP-OH **14** (1 eq., 250 mg, 0.499 mmol) in 2 mL DCM was added dropwise. The reaction was kept at 0 °C and stirred under TLC monitoring for 1 h. As TLC analysis indicated complete consumption of the starting material, the reaction was purified *via* column chromatography (silica, wet loading, PE/EtOAc=15%). 306 mg (0.460 mmol) of compound **14a** was obtained as an orange solid.

Yield: 92%. ^{1}H-NMR (700 MHz, $CDCl_3$): δ=8.25 – 8.20 (m, 2H, Ar-H), 8.12 – 8.08 (m, 1H, Ar-H), 7.91 (d, J=8.5 Hz, 1H, Ar-H), 7.61 (d, J=2.0 Hz, 1H, Ar-H), 7.51 – 7.47 (m, 2H, Ar-H), 7.31 – 7.26 (m, 2H, Ar-H), 6.70 – 6.65 (m, 2H, Ar-H), 4.58 – 4.42 (m, 2H, -CH_2-O-), 4.07 (s, 4H, -CH_2-), 4.04 – 3.96 (m, 1H, -CH-), 1.48 (s, 18H, *tert*-butyl -CH_3), 1.47 (d, J=7.0 Hz, 3H, -CH_3). ^{13}C-NMR (176 MHz, $CDCl_3$): δ=169.91, 169.90, 161.87, 155.56, 152.55, 152.54, 148.80, 147.95, 146.08, 145.54, 137.43, 128.37, 127.91, 126.26, 125.75, 125.72, 125.41, 125.30, 121.98, 115.73, 112.72, 82.31, 73.03, 54.60, 33.42, 28.24, 17.85 ppm. HRMS (ESI$^+$) *m/z:* exact mass: 665.2585 Da, observed: 688.2472 Da [M+Na]$^+$, calculated: 688.2477 Da [M+Na]$^+$, error: 0.5 mDa.

BODIPY Derivatives

(5,5-Difluoro-1,3,7,9-tetramethyl-5*H*-4λ4,5λ4-dipyrrolo[1,2-c:2',1'-*f*][1,3,2]diazaborinin-10-yl)methyl acetate (*Tetramethyl-BODIPY-OAc*) 15

Tetramethyl-BODIPY-OAc **15** was synthesised in accordance with three modified literature procedures.[4-6] 2,4-Dimethyl pyrrole (2 eq., 5.41 mL, 52.55 mmol) was placed into a three-neck round-bottom flask, dissolved in 350 mL DCM and cooled to 0 °C. Acetoxyacetyl chloride (1.2 eq., 3.39 mL, 31.53 mmol) was added *via* a syringe, and the reaction was stirred for 2 h at 40 °C. Subsequently, the reaction mixture was cooled to 0 °C and DIPEA (6 eq., 27.46 mL, 157.65 mmol) was added. The cooling bath was removed, and the resulting yellow/red solution was stirred for 15 min at RT. The reaction mixture was again cooled to 0 °C, and BF_3•OEt_2 (9 eq., 29.19 mL, 236.48 mmol) was added dropwise over 15 min, resulting in a colour change to dark red. The cooling bath was removed, and the reaction was stirred at RT for 3 h before the solvent was removed *in vacuo*, yielding a wet red solid. After purification *via* MPLC (dry loading, 100 g silica, PE/EtOAc=0→25%), 2.3 g (7.18 mmol) of compound **15** was isolated as orange-green crystals.

Yield: 27%. ^{1}H-NMR (400 MHz, $CDCl_3$): *δ*=6.08 (s, 2H, BODIPY, =CH-), 5.29 (s, 2H, BODIPY meso -CH_2-), 2.53 (d, *J*=1.3 Hz, 6H, BODIPY, -CH_3), 2.35 (s, 6H, BODIPY -CH_3), 2.13 (s, 3H, Acetyl -CH_3) ppm. ^{13}C-NMR (101 MHz, $CDCl_3$): *δ*=170.67, 156.74, 141.60, 133.43, 132.77, 122.44, 57.45, 20.71, 15.73, 14.80 ppm. HRMS (ESI$^+$) *m/z:* exact mass: 320.1508 Da, observed: 343.1416 Da $[M+Na]^+$, calculated: 343.1400 Da $[M+Na]^+$, error: 1.6 mDa.

(1,3,5,5,7,9-Hexamethyl-5*H*-4 λ^4,5 λ^4-dipyrrolo[1,2-c:2',1'-*f*][1,3,2]diazaborinin-10-yl)methanol (*Hexamethyl-BODIPY-OH*) 16

Hexamethyl-BODIPY-OH **16** was synthesised in accordance with a literature procedure.[7] Tetramethyl-BODIPY-OAc **15** (1 eq., 2.3 g, 7.18 mmol) in 100 mL absolute ether (absolutised over Na and benzophenone) was placed into a three-neck round-bottom flask. MeMgI (3 M solution in ether, 25 eq., 60 mL, 180 mmol) was added dropwise to the orange solution, and the reaction was stirred at RT. After 16 h, the reaction was cooled to 0 °C, quenched by slowly adding a saturated aqueous NH_4Cl solution and transferred into a separating funnel. The organic phase was washed with NH_4Cl solution and brine, dried over anhydrous Na_2SO_4, filtered and dried *in vacuo*. Subsequently, the crude material was re-dissolved in 60 mL THF, and LiOH•5H_2O (5 eq., 1.51 g, 35.87 mmol) dissolved in 60 mL ddH_2O was added. TLC analysis (PE/EtOAc=4/1) after 2 h indicated complete consumption of the starting material, and the reaction mixture was diluted with EtOAc, transferred to a separating funnel, and washed with saturated aqueous NH_4Cl solution and brine. The organic layer was dried over anhydrous Na_2SO_4, filtered and concentrated *in vacuo*. The crude product was purified *via* MPLC (dry loading onto AlOx, 40 g silica, PE/EtOAc=0→100%), yielding 1.0 g (3.79 mmol) of the desired compound **16** as orange/pink crystals.

Yield: 53%. ^{1}H-NMR (400 MHz, $CDCl_3$): *δ*=6.08 (s, BODIPY-C=CH-C, 2H), 4.95 (s, -CH_2-, 2H), 2.52 (s, BODIPY-CH_3, 6H), 2.46 (s, BODIPY-CH_3, 6H), 0.18 (s, BODIY-B-CH_3, 6H) ppm. ^{13}C-NMR (101 MHz, $CDCl_3$): *δ*=152.98, 138.38, 137.12, 130.73, 122.66, 56.55, 16.70, 16.07 ppm. HRMS (ESI$^+$) *m/z:* exact mass: 270.1830 Da, observed: 271.1984 Da $[M+H]^+$, calculated: 271.1976 Da $[M+H]^+$, error: 0.8 mDa.

(2,8-Diiodo-1,3,5,5,7,9-hexamethyl-5*H*-4λ4,5λ4-dipyrrolo[1,2-c:2',1'-*f*][1,3,2]diazaborinin-10-yl)methanol (*Me_6I_2-BODIPY-OH*) 17

Me_6I_2-BODIPY-OH **17** was synthesised by modifying two procedures from the original literature.[4, 7] The starting material **16** (1 eq., 1.02 g, 3.79 mmol) was placed into a 250 mL flask and dissolved in 100 mL THF. N-iodosuccinimide (NIS; 3.5 eq., 2.98 g, 13.25 mmol) dissolved in 20 mL dry THF was added to the solution *via* a

syringe. The reaction was stirred under TLC monitoring for 5 h at RT and subsequently diluted with 100 mL EtOAc and transferred to a separating funnel. The organic layer was washed with ddH$_2$O and brine, dried over anhydrous Na$_2$SO$_4$, filtered and dried *in vacuo*, yielding 1.7 g (3.35 mmol) of PPG **17** as a pink, crystalline solid.

Yield: 89%. ^{1}H-NMR (400 MHz, CDCl$_3$): *δ*=4.95 (s, -CH$_2$-, 2H), 2.57 (s, BODIPY-CH$_3$, 6H), 2.54 (s, BODIPY-CH$_3$, 6H), 0.19 (s, BODIPY-B-CH$_3$, 6H) ppm. ^{13}C-NMR (101 MHz, CDCl$_3$): *δ*=153.58, 139.56, 137.24, 130.65, 87.41, 57.04, 29.84, 23.95, 18.51 ppm. HRMS (ESI$^+$) *m/z:* exact mass: 521.9836 Da, observed: 522.9914 Da [M+H]$^+$, calculated: 522.9909 Da [M+H]$^+$, error: 0.5 mDa.

(2,8-Diiodo-1,3,5,5,7,9-hexamethyl-5*H*-4λ^4,5λ^4-dipyrrolo[1,2-c:2',1'-*f*][1,3,2]diazaborinin-10-yl)methyl (4-nitrophenyl) carbonate 17a

NPC (2.5 eq., 96.54 mg, 0.479 mmol) was dissolved in 1 mL DCM and pyridine 4.65 equivalents, 72 µL, 0.891 mmol) was added at 0 °C. The resulting suspension was stirred, and Me$_6$I$_2$-BODIPY-OH **17** (1 eq., 100 mg, 0.192 mmol) in 1 mL DCM was added dropwise. The reaction was kept at 0 °C and stirred under TLC monitoring for 30 min. As TLC analysis indicated full consumption of the starting material, the reaction was purified *via* column chromatography (silica, wet loading, PE/DCM=15→30%) and 89 mg (0.130 mmol) of compound **17a** was obtained as a pink solid.

Yield: 68%. ^{1}H-NMR (400 MHz, CDCl$_3$): *δ*=8.34 – 8.27 (m, Ar-H, 2H), 7.45 – 7.37 (m, Ar-H, 2H), 5.64 (s,-CH$_2$- 2H), 2.57 (s, BODIPY-CH$_3$, 6H), 2.52 (s, BODIPY-CH$_3$, 6H), 0.22 (s, BODIPY-B-CH$_3$, 6H) ppm. ^{13}C NMR (176 MHz, CDCl$_3$): δ=155.23, 154.49, 152.15, 145.63, 139.30, 130.94, 130.13, 125.45, 121.60, 88.17, 62.93, 29.72, 18.67, 18.54 ppm. HRMS (ESI$^-$) *m/z:* exact mass: 686.9898 Da, observed: 685.9830 Da [M-H]$^-$, calculated: 685.9826 Da [M-H]$^-$, error: 0.4 mDa.

Standard Fmoc-SPPS

Coupling of the First Amino Acid

Reactions were performed in syringe reactors outfitted with solid-phase extraction filtration frits. Rink amide-AM resin (0.74 mmol/g, 100-200 mesh, 1 eq., 0.25 mmol, 338 mg) was swelled for at least 2 h (preferably overnight) in DMF. Prior to the first amino acid coupling, Fmoc-deprotection from the Rink amide-AM resin linker was achieved by applying 50% piperidine in DMF (2x1 min), followed by a 1-min flow-wash (30 mL/min flow) using DMF. Activation of the Fmoc-amino acids (4 eq.) before the first coupling was carried out with 0.5 M HATU in DMF (3.95 eq., 1.975 mL) and DIPEA (4 eq., 174 µL, 1 mmol). The activated reagents were added to the swollen resin and coupled for 15 min. Manual stirring was applied every 5 min, followed by a 1-min flow-wash with DMF after coupling.

Elongations of the Peptide Sequence

Fmoc deprotection to prepare the last amino acid on resin for the subsequent coupling step was achieved using 50% piperidine in DMF (2x 1 min). Activation of the Fmoc-amino acid (4 equivalents, 1 mmol) was achieved with 0.5 M HATU (3.95 eq., 1.975 mL, 0.9875 mmol) in DMF and DIPEA (4 eq., 174 µL, 1 mmol) in DMF. The activated reagent mix was added to the resin and coupled for 10 min. Manual stirring was applied every 5 min, followed by a 1-min flow-wash with DMF to prepare the resin for the next coupling cycle.

Cleavage of the Peptide from the Resin

Global sidechain deprotection and simultaneous cleavage of the peptides from the resin were achieved by applying a cleavage cocktail (TFA/TIPS/ddH_2O=90/5/5, 1.5 mL/100 mg peptide on resin) for 2 h. After the cleavage, the remaining product was washed from the resin with a freshly prepared cleavage cocktail (1x5 min) and combined with the cleavage solution in a 15 mL Falcon tube. The cleavage mixture was concentrated under a N_2 stream, and cold Et_2O (-20 °C) was added to precipitate the crude product. The suspension was centrifuged for 5 min at 3,500 x *g,* and the supernatant was decanted. The pellet was washed by resuspending it in cold Et_2O and then centrifuging. The procedure was repeated twice. The crude pellet was dried under an N_2 stream and dissolved in A/B solvent (0.1% TFA in ddH_2O/0.08% TFA in ACN=1/1). The solution was lyophilised to obtain the crude peptide.

Oxidative Peptide Folding

After lyophilisation, the crude peptide was re-dissolved in aqueous 0.1 M NH_4HCO_3 buffer (pH=8.2) and stirred for 24 h at RT. Peptide concentrations were kept below 0.5 mM to prevent intermolecular disulfide bridge formation. Reaction progress was observed *via* LC-MS. After oxidative folding was completed, the reaction was quenched through acidification with TFA to a pH=2-3. The crude peptide solution was either lyophilised or directly loaded onto a preparative C_{18}-RP-HPLC column for purification.

On-Resin Photocaging to Produce Photoprobes 1, 2, 3, 4

The fully protected OT and VP were synthesised manually *via* standard Fmoc-SPPS.[8] The Fmoc group was removed from the resin, and the peptide was assembled as described in the general Fmoc-SPPS procedure. Once the peptide assembly was complete and the N-terminal Fmoc-group was removed, test cleavages with a standard cleavage cocktail (TFA/ddH_2O/TIPS=90/5/5) were performed to confirm the masses of the assembled peptide sequences *via* LC-MS, followed by drying the rest of the fully protected peptides on-resin *in vacuo*. The resins were split, and 1/5th of the peptides on-resin (0.05 mmol) was each used for the subsequent N-terminal caging.

For the subsequent N-terminal caging, the activated PPGs **9a** and **14a** (5 eq., 0.25 mmol) were re-dissolved in 2 mL 20% pyridine in DMF. The solution was placed in the syringe reactor, which was spun for 3 days. The reaction progress was monitored by test cleavages and LC-MS analysis. Once the coupling was complete, the resin was flow-washed with DCM and dried *in vacuo*, followed by peptide cleavage and global deprotection of the side chains and the *tert*-butyl ester of the PPGs. The linear caged peptides were precipitated, washed, and lyophilised as described above. Crude yields are based on the 50 µM scale used for on-resin photocaging.

After lyophilisation, the linear crude peptides were re-dissolved and oxidatively folded using the same procedure stated above. To prevent intermolecular disulfide bond formation, we maintained the peptide concentration in the oxidation buffer below 0.5 mM (~1 mg peptide/10 mL oxidation buffer). Upon completion of folding as indicated by LC-MS, we acidified the solution with TFA to pH=2-3 and purified the solution (direct loading) *via* RP-HPLC. Yields after TFA cleavage, folding and purification, and total overall yields based on scale (0.05 mmol for on-resin photocaging, 0.005 mmol for in-solution photocaging) are listed below (**Table S1**).

Reduced DCMAC-OT photoprobe 1r

TFA cleavage yield (crude) 79% (52.78 mg, 0.039 mmol), white powder. MS (ESI$^+$) *m/z:* exact mass: 1341.5006 Da, observed: 1343.48 Da $[M+H]^+$, calculated: 1342.5078 Da $[M+H]^+$, error: 0.97 Da.

DCMAC-OT photoprobe 1

Folding and RP-HPLC purification yield: 18% (9.6 mg, 0.007 mmol), white powder. HRMS (ESI^+) *m/z:* exact mass: 1339.4849 Da, observed: 1340.4936 Da $[M+H]^+$, calculated: 1340.4922 Da $[M+H]^+$, error: 1.4 mDa.
Overall yield: 14% (>95% purity).

Reduced DCMAC-VP photoprobe 2r

TFA cleavage yield (crude) 47% (33 mg, 0.023 mmol), white powder. MS (ESI^+) *m/z:* exact mass: 1418.5020 Da, observed: 710.49 Da $[M+2H]^{2+}$, calculated: 710.2583 Da $[M+2H]^{2+}$, error: 0.23 Da.

DCMAC-VP photoprobe 2

Folding and RP-HPLC purification yield: 17% (6.3 mg, 0.004 mmol), white powder. HRMS (ESI^+) *m/z:* exact mass: 1416.4863 Da, observed: 709.2515 Da $[M+2H]^{2+}$, calculated: 709.2505 Da $[M+2H]^{2+}$, error: 1 mDa.
Overall yield: 8% (>95% purity).

Reduced DCANBP-OT photoprobe 3r

TFA cleavage yield (crude) 71% (50.3 mg,0.035 mmol), yellow powder. MS (ESI^+) *m/z:* exact mass: 1422.5584 Da, observed: 1423.47 Da $[M+H]^+$, calculated: 1423.5657 Da $[M+H]^+$, error: 0.1 Da.

DCANBP-OT photoprobe 3

Folding and RP-HPLC purification yield: 28% (14 mg, 0.010 mmol), yellow powder. HRMS (ESI^+) *m/z:* exact mass: 1420.5428 Da, observed: 711.2790 Da $[M+2H]^{2+}$, calculated: 711.2787 Da, error: 0.3 mDa.
Overall yield: 20% (>95% purity).

Reduced DCANBP-VP photoprobe 4r

TFA cleavage yield (crude) 80% (60.4 mg,0.04 mmol), yellow powder. HRMS (ESI^+) *m/z:* exact mass: 1499.5598 Da, observed:749.7792 Da $[M+2H]^{2+}$, calculated: 749.7794Da $[M+2H]^{2+}$, error: 0.2 mDa.

DCANBP-VP photoprobe 4

Folding and RP-HPLC purification yield: 12% (7.38 mg, 0.005 mmol), yellow powder. HRMS (ESI^+) *m/z:* exact mass: 1497.5442 Da, observed: 749.7790 Da $[M+2H]^{2+}$, calculated: 749:7794 Da, error: 0.4 mDa.
Overall yield: 10% (>95% purity).

Solution-Phase Photocaging to Produce Photoprobes 5, 6

The folded neuropeptide (OT/VP; 1 eq., 5 mg, 0.005 mmol) was placed into a round-bottom flask and dissolved in 1.2 mL 15% DMF in THF at 0 °C. The activated Me_6I_2-BODIPY **17a** (3 eq., 10 mg, 0.015 mmol), dissolved in 1.5 mL 10% pyridine in THF, was added dropwise *via* a syringe and the reaction mixture was stirred under LC-MS monitoring for 44 h at 0 °C. After full consumption of the starting peptide, the solvent was removed *in vacuo,* and the reaction mixture was purified *via* RP-HPLC (ddH_2O, ACN, no TFA). After lyophilisation, the desired Me_6I_2-BODIPY derivatives **5** and **6** were obtained as a pink powder. Yields are based on the amount of pure neuropeptide used in the reactions.

Me_6I_2-BODIPY-OT photoprobe 5

RP-HPLC purification yield: 56% (4.33 mg, 0.003 mmol), pink powder. HRMS (ESI^+) *m/z:* exact mass: 1554.3994 Da, observed: 1555.4073 Da $[M+H]^+$, calculated: 1555.4066 Da, error: 0.7 mDa.
Overall yield: 56% (>95% purity)

Me_6I_2-BODIPY-VP photoprobe 6

RP-HPLC purification yield: 24% (1.8 mg, 0.001 mmol), pink powder. HRMS (ESI^+) *m/z:* exact mass: 1631.4007 Da, observed: 1632.4091 Da $[M+H]^+$, calculated: 1632.4080 Da, error: 1.1 mDa.
Overall yield: 24% (>95% purity)

Final Compound Overview

Table S1: Characteristics of final compounds.

Final compound	MW [Da]	Mass obs. [Da]	Mass ion	Mass calc. [Da]	Yield
DCMAC-OT **1**	1340.45	1340.4936	$[M+H]^+$	1340.4922	*14%
DCMAC-VP **2**	1417.49	709.2515	$[M+2H]^{2+}$	709.2505	*8%
DCANBP-OT **3**	1421.56	711.2790	$[M+2H]^{2+}$	711.2787	*20%
DCANBP-VP **4**	1498.61	749.7790	$[M+2H]^{2+}$	749.7794	*10%
Me_6I_2-BODIPY-OT **5**	1555.16	1555.4073	$[M+H]^+$	1555.4066	**56%
Me_6I_2-BODIPY-VP **6**	1632.21	1632.4091	$[M+H]^+$	1632.4080	**24%

Molecular weight (MW) observed in high-resolution masses and the corresponding calculated monoisotopic masses. Overall yields >95%: * on-resin photocaging, oxidative folding and RP-HPLC; ** in-solution photocaging and RP-HPLC.

Photochemical Characterisation

Compounds were placed in brown 1.5 mL LC-MS vials and dissolved in 3% ACN/PBS (pH=7.4) to obtain 500 µM solutions. After dilution to 50 µM in PBS, the UV/Vis absorbance was recorded between 300 and 600 nm using a NanoDrop 2000 (Thermo Fisher Scientific). 0.3% ACN/PBS (pH=7.4) was measured as a blank and subtracted before each compound. Curves were depicted, and peak maxima were analysed using GraphPad Prism 9. In accordance with these results, LEDs (New Energy) with peak wavelengths in proximity to measured absorbance maxima and LED drivers (Recom Power) were purchased from DigiKey at:

- UV-A (365 nm): LST1-01G01-UV01-00, luminous flux @ 500 mA/25 °C=875 mW
- Violet (405 nm): LST1-01G01-UV04-00, luminous flux @ 500 mA/25 °C=930 mW
- Blue (450 nm): LST1-01G01-RYL1-00, luminous flux @ 500 mA/25 °C=600 mW
- Green (527 nm): LST1-01G01-GRN1-00, luminous flux @ 500 mA/25 °C=270 mW
- LED driver: RACD06-500-LP.

Uncaging was tested with OT photoprobes **1**, **3**, and **5** (uncaging behaviour independent of attached peptide, tested for compound **2**, not shown). 30 µL of the 50 µM solutions in 0.3% ACN/PBS were placed into brown LC-MS vials with 200 µL polypropylene inlets. LEDs were placed over the vials, and the samples were irradiated directly at the respective times. After irradiation, the vials were sealed, and 25 µL were injected into the RP-HPLC system (Column: Kromasil 300-5-C_{18} HPLC column (4.6 x 150 mm, 5 µm, 300 Å gradient 10-90% B in 27 min; A: 0.1% TFA in ddH_2O; B: 0.08% TFA in ACN; no acid additives for BODIPY compound **5**)). 0.3% ACN/PBS (pH=7.4) was measured as a blank and subtracted before each compound. Curves were depicted and analysed with Origin 2021b. The percentage of remaining caged compounds after the respective irradiation times was determined by integrating the compound peak normalised to the absorbance at t=0 s (100%) and the baseline (0%).

MTT Assays

Cell Culture and Treatment

HEK-293 wild-type cells were stored in 1 mL 5% DMSO in fetal bovine serum (FBS) in liquid N_2. Once the cells were removed from storage, they were thawed quickly and diluted 1/9 with growth medium (Dulbecco's modified Eagle medium (DMEM) containing 10% FBS, 2 mM L-glutamine, and 1 mM Na pyruvate). The cell suspension was centrifuged (3 min at 161 x *g*), and the supernatant was aspirated. The cells were resuspended in a growth medium, seeded into T-25 cell culture flasks, and incubated (37 °C, 5% CO_2) for 24 h. Subsequently, the medium was exchanged for the growth medium (DMEM, 10% FBS, 2 mM L-glutamine, 1 mM Na pyruvate, 1% Penicillin-Streptomycin (Gibco, 10,000 U/mL)). Cells were again incubated and grown to 80-90% confluency, washed with PBS, detached with 2.5 mL StableCell trypsin solution (0.5 g porcine trypsin, 0.2 g ethylenediaminetetraacetic acid (EDTA), 4 Na/L of Hanks′ Balanced Salt Solution with phenol red) and centrifuged (3 min at 161 x *g*). The supernatant was aspirated, the pellet resuspended in the selection medium, and the cells were seeded into T-75 cell culture flasks. Cells were incubated and passaged at least twice (as previously described) at 80-90% confluency before being used for subsequent assays.

The MTT (3-(4,5-dimethylthiazol-2-yl)-2,5-diphenyltetrazolium bromide) assay is a colourimetric method to assess cell viability and cytotoxicity. It relies on the enzymatic reduction of the yellow MTT to its purple-coloured formazan derivative. This only happens in viable cells, not in dead or quiescent cells. Hence, the formation of formazan can be used to determine metabolic activity and cell viability. This is quantified by a colourimetric readout, measuring absorbance at 570 nm.[9]

Stock solutions (500 µM) of OT, OT-photoprobes (**1**, **3**, **5**), *in situ* uncaged (60 s irradiation with LED: **1**, **3**: 405 nm; **5**: 527 nm) OT-photoprobes (**1***, **3***, **5***) in PBS and Staurosporine (SSp) in PBS were prepared and stored at -20 °C. Before the assay, the stocks were thawed, and dilutions of 1 and 10 µM (10x 0.1/1 µM) in growth medium were prepared in the dark and covered with aluminium foil until needed. The assay was carried out as biological triplicates. The cells were detached at 80-90% confluency, centrifuged, and the supernatant was aspirated. The pellet was resuspended in the growth medium, and the cells were subsequently counted with a Neubauer counting chamber. 10,000 cells/well in 90 µL growth medium were seeded into 96-well plates (sterile transparent polystyrene Sarstedt tissue culture treated round base 96-well plates) and incubated for 1 h at 37 °C. 10 µL of the prepared 10x solutions were added to the wells. Cells with 100 µL of growth medium were used as a growth control (normal metabolic activity), PBS as a vehicle control, and 100 µL of medium without cells as a death control (no metabolic activity). The plate was incubated at 37 °C. After 48 h, 10 µL of a 5 mg/mL MTT solution in Hank's Balanced Salt Solution was added, and the plate was incubated for an additional 4 h at 37 °C. The plates were removed from the incubator, and 100 µL solubilisation solution (8.82 mL ddH_2O, 180 µL AcOH, 6 mL DMF, 2.4 g sodium dodecyl sulphate, sonicated and pH adjusted with 450 µL 6 M NaOH) was added and subsequently placed onto a shaker for 30 min to facilitate cell lysis and dissolve the formed formazan. Afterwards, the plate was placed into a Tecan Spark multimode microplate reader (Tecan, Männedorf, Switzerland), and after 5 min of pre-shaking, the absorbance at 570 nm was measured. The obtained values were depicted in GraphPad Prism 9, averaged, and normalised to medium without cells (0% cell viability) and to medium with cells (100% cell viability).

Functional FRET Assays – IP_1

Cell Culture and Treatment

HEK-293 cells stably expressing the GFP-tagged human G_q-GPCR of interest (hOTR, $hV_{1a}R$, $hV_{1b}R$) under the same promoter as a Neomycin-Geneticin resistance gene were stored in 1 mL 5% DMSO in fetal FBS in liquid N_2. Once the cells were removed from storage, they were thawed quickly and diluted 1/9 with growth medium (DMEM containing 10% FBS, 2 mM L-glutamine, and 1 mM Na pyruvate). The cell suspension was centrifuged (3 min at 161 x *g*), and the supernatant was aspirated. The cells were resuspended in a growth medium, seeded into T-25 cell culture flasks, and incubated (37 °C, 5% CO_2) for 24 h. Subsequently, the medium was exchanged for the selection medium (DMEM, 10% FBS, 2 mM L-glutamine, 1 mM Na pyruvate, G-418 sulfate antibiotic (Geneticin, Gibco, 50 mg/mL) 160 µL/10 mL). Cells were again incubated and grown to 80-90% confluency, washed with PBS, detached with 2.5 mL StableCell trypsin solution (0.5 g porcine trypsin, 0.2 g EDTA, 4 Na/L of Hanks′ Balanced Salt Solution with phenol red) and centrifuged (3 min at 161 x *g*). The supernatant was aspirated, the pellet resuspended in the selection medium, and the cells were seeded into T-75 cell culture flasks. Cells were incubated and passaged at least twice (as previously described) at 80-90% confluency before being used for subsequent assays.

The commercially available IP_1-accumulation assay from Cisbio (IP-One Gq kit HTRF) is a Förster resonance energy transfer (FRET) based, time-resolved fluorescence assay that utilises the formation of endogenous IP_1 upon receptor activation. The addition of LiCl blocks the degradation of IP_1 to D-myo-inositol, resulting in the accumulation of endogenous IP_1. Excitation (340 nm) of a FRET donor (anti-IP_1-Eu-Cryptate) leads to fluorescence (620 nm). If a FRET acceptor molecule, a labelled IP_1 derivative (d2-IP_1), is present at the antibody's binding site, an easily distinguishable bathochromically shifted FRET signal (665 nm) is generated.[10] Endogenous IP_1 competes with d2-IP_1 and leads to attenuation of the FRET signal that is then detected and used to determine the amount of endogenous IP_1 formed and, hence, the number of ligands bound to the GPCR.[10]

Stock solutions (1 mM) of photoprobes **1**-**6** in 30% ACN/ddH_2O and OT and VP in ddH_2O were prepared and stored at -20 °C. Before the assay, the stocks were thawed, and semi-logarithmically spaced dilutions in the provided stimulation buffer were prepared (**1**-**6**, OT: 3 µM – 30 pM; VP: 1 µM – 30 pM) in the dark and covered with aluminium foil until needed. The assay was performed according to the manufacturer's procedure. The assays for each receptor type were performed as biological triplicates. The cells were detached at 80-90% confluency, centrifuged, and the supernatant was aspirated. The pellet was resuspended in the selection medium, and the cells were subsequently counted with a Neubauer counting chamber. 10,000 cells/well in 50 µL of selection medium were seeded into 384-well plates (sterile white polystyrene Greiner tissue culture-treated flat-bottom multi-well plates) and incubated for 48 h. On the day of the assay, the medium was removed by pushing the plate onto tissue paper, and 5 µL of the provided stimulation buffer was added to each well. The plate was incubated for 15 min before 5 µL of the compounds was added. OT (for hOTR) or VP (for $hV_{1a}R$ and $hV_{1b}R$) was used as a positive control, and stimulation buffer (vehicle) as a negative control. Each concentration was measured as a technical triplicate, and after addition, the plate was incubated for 1 h before 5 µL d2-IP_1 and anti-IP_1-cryptate in the provided lysis and detection buffer were added sequentially. The plate was equilibrated for 1 h at RT, followed by readout with a Tecan Spark multimode microplate reader (Tecan, Männedorf, Switzerland). The results were determined as the emission ratio (665 nm/620 nm) after excitation at 340 nm, depicted using GraphPad Prism 9, and averaged and normalised to the highest concentration of the positive control (100%=3 µM OT / 1 µM VP) and the negative control (0%=stimulation buffer). EC_{50} values were determined by fitting a non-linear regression of the normalised mean values.

Confocal Microscopy – Immunostaining HEK-293 Cells

Cell Culture, Treatment, and Immunocytochemistry

HEK-293 cells stably expressing GFP-tagged hOTR were maintained with DMEM containing 10% FBS, 2 mM L-glutamine, 1 mM sodium pyruvate and 500 µg/mL G-418. For experimental setups, cells were seeded at 10,000 cells/well on poly-D-lysine-coated glass coverslips in black 24-well plates (Cellvis) to prevent light spillover and left to recover for 24 h. The following day, the growth medium was replaced with serum-free medium to synchronise the cells for an additional 24 h. Next, compounds were added to the wells and irradiated without delay with their respective wavelengths, as follows: (**1**: 100 nM) 365 nm, 30 s, 60 s; (**3**: 100 nM) 405 nm, 30 s, 60 s; and (**5**:10 nM) 527 nm, 10 s, 30 s. The plates were incubated for 1 h to mimic the conditions of the IP_1 assays before being processed for immunocytochemistry. Dark control plates were incubated directly after compound addition without prior irradiation. Plates were washed with ice-cold PBS containing PhosSTOP phosphatase inhibitors (Roche) in the dark, then fixed with 4% paraformaldehyde (PFA) in PBS. After 30 min, the PFA solution was removed, and the cells were washed twice with PBS to remove excess PFA.

To prevent nonspecific antibody binding, we incubated the cells with 5% normal donkey serum (Jackson ImmunoResearch), 2% bovine serum albumin (BSA), and 0.2% Triton X-100 for 1 h at RT. After removal of the blocking solution, the cells were exposed to 2% normal donkey serum, 0.1% BSA, and 0.2% Triton X-100 in PBS containing FITC-GFP (Abcam, #ab6662, 1:1000) and pCREB (Millipore, #05-667, 1:250) antibodies overnight at 4 °C. The following day, coverslips were washed with PBS and treated with 2% BSA in PBS containing anti-goat Cy2 (Jackson Immuno Research, 1:500), anti-mouse Cy5 (Jackson Immuno Research, 1:500), as well as phalloidin-555 (Invitrogen, 1:500) for f-actin labelling and Hoechst 33342 (1:10,000) as nuclear counterstain for 2 h at RT. Finally, coverslips were washed with PBS before being mounted in glycerol-gelatine. Cells were imaged using a Zeiss LSM710 confocal microscope, and fluorescent intensity was analysed with ImageJ. Student's t-tests were used to determine statistical significance.

Confocal Microscopy – Immunostaining Primary Rat Hippocampal Neurons

Primary Neuronal Cultures, Treatment, and Immunocytochemistry

Adult female Sprague-Dawley rats and their embryos (males and females, embryonic day 18) were used to prepare primary hippocampal neurons. All research procedures involving the use of animals were conducted under the Australian Code of Practice for the care and use of animals for scientific purposes and were approved by the University of Queensland Animal Ethics Committee (2021/AE000511).

Primary hippocampal neurons were isolated from male and female embryonic day 18 Sprague Dawley rat brains. Hippocampi were isolated and dissociated with 30 U of papain suspension (Worthington) for 20 min in a 37 °C water bath. A single-cell suspension was obtained by triturating tissues with a pipette and then plated at a density of 50,000 cells (per coverslip) on poly-L-lysine-coated 24-well coverslips in Neurobasal growth medium supplemented with 2 mM GlutaMAX, 1% penicillin/streptomycin, and 2% B-27. Neurons were maintained in a humidified 5% CO_2 tissue culture incubator at 37 °C and were kept in Neurobasal growth medium, which was replenished twice a week. Cells were processed at 16 days *in vitro* (DIV16).

Neurons were treated with aCSF (25 mM HEPES, 120 mM NaCl, 5 mM KCl, 2 mM $CaCl_2$, 2 mM $MgCl_2$, 30 mM D-glucose, pH=7.4; negative control) or compounds: DCMAC-OT **1** or DCANBP-OT **3** at both 50 nM or 100 nM or OT (positive control) at 100 nM in aCSF. Following immediate uncaging (except for 100 nM dark controls of DCMAC-OT **1** or DCANBP-OT **3**) and incubation of neurons in aCSF at 37 °C for 1 h, the neurons were fixed in a parafix solution (4% PFA, 4% sucrose in PBS) for 10 min. Neurons were then permeabilised with 0.25% Triton X-100 in PBS for 10 min and blocked with 10% normal goat serum (NGS) for 1 h at RT. After blocking, neurons were incubated with a monoclonal rabbit anti-pCREB antibody (9198S, Cell Signalling Technology) and a polyclonal chicken anti-MAP2 antibody (ab92434, Abcam), diluted in 3% NGS at 1/500 or 1/5000, respectively, overnight at 4 °C. Primary antibodies were revealed by incubating Alexa-568-coupled anti-rabbit IgG secondary (A21245) and Alexa-488-coupled anti-chicken IgG secondary (A32931) for 1 h at RT. Lastly, neurons were labelled with a nuclear-specific dye (DAPI) for 10 min at RT. Images were collected with a 63X oil-immersion objective on a Zeiss LSM710 confocal microscope. Fluorescence intensities were quantified using ImageJ software (National Institutes of Health).

Two-Photon Microscopy

Image Acquisition and 2PE OT uncaging

HEK-293 cells stably overexpressing hOTR-GFP were imaged using a two-photon laser scanning microscope (Ultima Investigator Multiphoton Imaging System, Bruker) equipped with a Mai Tai Ti:Sapphire laser (Spectra-Physics) and a femtosecond laser (Spectra-Physics) and controlled by Prairie View software. The laser was tuned to 920 nm to excite eGFP, and images were acquired with a water-dipping objective (25x, 1.1 numerical aperture; Nikon). Image stacks (1024 x 1024 pixels, 0.1 µm/pixels) with 0.5 µm z-steps were collected before and 10 min after the uncaging protocol. All imaging experiments were performed at 35 °C. Cells were bathed in either aCSF or aCSF supplemented with 100 nM DCMAC-OT **1** or 100 nM DCANBP-OT **3**. Afterwards, the region of interest for uncaging was positioned along the cell membrane of the targeted cells to ensure localised photolysis of the OT compounds. The Ti: Sapphire laser was tuned to 730 nm to uncage DCMAC-OT **1** (60 pulses, 30 ms, 0.3 Hz) or 780 nm to uncage DCANBP-OT **3** (60 pulses, 45 ms, 0.25 Hz).

Ex vivo

Experimental Models and Subject Details

The following mouse lines were used in this study: *Sst-IRES-Cre, B6J.Cg-Ssttm2.1(cre)Zjh/MwarJ* (The Jackson Laboratory). All mice were backcrossed to C57BL/6J or C57BL/6JRj mice for at least 6 generations. Male and female mice were used at postnatal day 52-128. Animals used were group-housed. Mice were housed under a 12-h light/dark cycle. Experiments were conducted during the light phase of the schedule. All procedures were performed in accordance with the guidelines of the Medical University of Vienna and under approved licenses by the Austrian Ministry of Science (2022-0.506.969).

Stereotactic AAV Injections

8-14 weeks old mice underwent stereotaxic surgery. Anaesthesia was induced with 5% isoflurane and maintained with 1.5%–2% isoflurane. Carprofen (4 mg/kg) and Lidocaine (2-5 mg/kg) were given subcutaneously before incision.

For injections, a small craniotomy (~1 mm diameter) was made using the following coordinates (distance from bregma [mm] / distance from midline [mm] / depth [mm] / angle [°]):

- Anterior cingulate cortex (ACC): 1.6 / 0.9 / 1.6 / 20
- Primary auditory cortex (Au1): 3.25 / ~4.4 / 0.9

A glass micropipette was inserted through a small durotomy to deliver the virus. The pipette was held in place for 3 min. 75-200 nL AAV were injected at 40 nL/min using a UMP3 microsyringe pump (World Precision Instruments, Sarasota, Florida). The pipette was held in place for an additional 2 min after the end of the injection. The pipette was then slowly retracted. The scalp incision was sutured, and post-surgery analgesics (4 mg/kg Carprofen) were injected subcutaneously every 24 h to aid recovery for the following two days. AAVs were allowed to express for at least 4 weeks before the mice were used for experiments. Optimal AAV titres were determined by a series of injections with increasing dilutions as well as injections into wild-type mice to test for spontaneous recombination of the double-floxed inverted open reading frame (DIO).

The following AAVs and titres were used:

- pAAV2/9-CBA-DIO-GCaMP6s-P2A-mBeRFP-WPRE-pA (VVF UZH # v828-9): 2.9 x 10^{12} GC/mL

***Ex vivo* epifluorescence imaging**

Mice were deeply anaesthetised with inhaled isoflurane and transcardially perfused with ~30 mL ice-cold sucrose-based solution (212 mM sucrose, 3 mM KCl, 1.25 mM $Na_2H_2PO_4$, 26 mM $NaHCO_3$, 1 mM $MgCl_2$, 0.2 mM $CaCl_2$, 10 mM glucose) oxygenated with carbogen gas (95% O_2, 5% CO_2, pH=7.4). The mice were decapitated, brains dissected, and 300 µM coronal slices were prepared on a Leica VT1200 vibratome (0.12 mm/s speed, 0.8 mm amplitude) in oxygenated cold sucrose solution. Slices were incubated for 10 min at 37 °C in oxygenated Ringer's solution (125 mM NaCl, 25 mM $NaHCO_3$, 1.25 mM NaH_2PO_4, 2.5 mM KCl, 2 mM $CaCl_2$, 1 mM $MgCl_2$, 25 mM glucose), then kept at RT. Imaging was performed on an Olympus BX51WI microscope equipped with an ORCA-Fusion Digital CMOS camera (Hamamatsu, #C14440-20UP) and a 10x UMPLFLN10XW objective (Olympus). Excitation and emission filters were set for GCaMP (470/40 nm, 525/50 nm; 495 nm beamsplitter, AHF #F46-002) and mBeRFP (575/32 nm, 625/32 nm, 604 nm beamsplitter; AHF #F46-804). Videos were acquired using a Hamamatsu Orca Fusion camera at 4 Hz with a 50 ms exposure time per frame. µManager software was used to control the camera.[11] The excitation light (CoolLED pE-300^{white}) was synchronised with the camera *via* a TTL trigger to avoid excessive bleaching. Imaging windows in cortical slices were selected based on clearly visible mBeRFP fluorescence as an indicator for sufficient GCaMP expression.[12] The bath temperature was set to 31 °C. Baseline recordings of 5 min were performed before data acquisition to allow the slices to adjust to the conditions. Slices were continuously submerged in recirculating oxygenated Ringer's solution throughout the whole recording session. To visualise viral expression levels, the red fluorescence channel (mBeRFP) was imaged during the initial 12 s (first 50 frames). For the rest of the recording, green emission was imaged to visualise GCaMP. For the OT bath application, slices were first recorded for a 3-min baseline period in aCSF in the absence of the peptide. Subsequently, 1 µM OT was bath-applied for 2 min. After 15 min of recording, 30 mM KCl was added to the bath to verify neuronal responsiveness by inducing calcium-dependent fluorescence increases. For *ex vivo* validation of DCMAC-OT **1**, brain slices were first recorded for a 3-min baseline, after which neurons were illuminated with a 405 nm external LED for 1 min, followed by a 3-minute LED-off period. Subsequently, 1-5 µM DCMAC-OT **1** was

bath-applied and continuously recirculated until the end of the experiment. 5 min after application of DCMAC-OT **1**, the 405 nm LED was switched on for 1 min to uncage DCMAC-OT **1**. 30 mM KCl was applied 10 min after. The total recording lasted 35 min. The 405 nm LED power was set to 10 mW (141 µW/mm^2 power density) at the plane of the imaged slice, measured with a Thorlabs power meter PM100D (S121C sensor). To test the specificity of receptor activation *via* OT release after uncaging, slices were incubated with OTA for ~7 min prior to imaging. During recordings, the same illumination and bath-application protocol described above was used, except that 10 µM OTA was continuously present throughout the experiment.

Data analysis was performed using a custom-written MATLAB script. Regions of interest (ROI) were selected based on mBeRFP expression. Movement corrections were performed by tracking manually selected markers in the imaging window. Fluorescence intensity was measured from each ROI across the whole video. Photobleaching was corrected by fitting a mono- or bi-exponential decay to the raw intensity measurement. Background fluorescence from the slice was subtracted from all ROIs. Since 405 nm illumination of GCaMP increases emission in a largely calcium-independent manner (close to the isosbestic point), the increase in intensity during 405 nm LED illumination periods was subtracted from the trace. Calcium-dependent emission changes should stay largely unaffected by this subtraction. ROIs were subsequently eliminated if the fluorescence increase in response to KCl was <2x SD of the 3 min baseline recording. $\Delta F/F_0$ was calculated by subtracting from the fluorescence intensity of each cell the mean baseline fluorescence and dividing by the mean baseline fluorescence. For illustration, the final average $\Delta F/F_0$ traces for each slice were separated into the three parts for 405 nm LED, DCMAC-OT **1** application in the dark, and 405 nm LED during DCMAC-OT **1** applications and merged into one figure aligned to the start of the 405 nm LED and DCMAC-OT **1** application, respectively. A cell was counted as responding to DCMAC-OT **1** uncaging if the mean response during the first minute of stimulation was >2x SD of the last minute before stimulation. Mean $\Delta F/F_0$ was calculated across the 60 s LED-illumination time window. For the OT bath application, the analysis window was shifted by 30 s (starting 30 s after OT first reached the bath) to reflect the slower onset of responses during the wash-in period of OT.

All data are represented as mean ± SEM.

For statistical analysis, data were tested for normality using the Shapiro-Wilk test. Since the data were non-normally distributed, the Mann-Whitney U test was used to compare uncaging responses with and without antagonist. *** indicated p-values of <0.001.

Caging rational

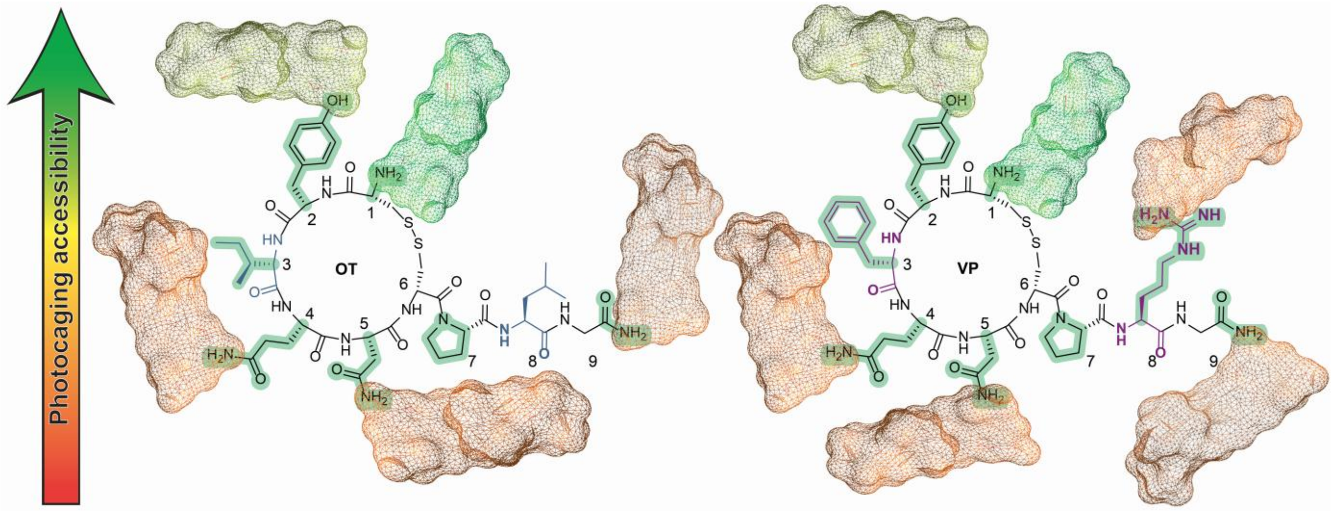

Figure S1. Possible points of attachment for PPGs on OT and VP. Electron densities of PPG (exemplary DCMAC) indicate chemically possible anchoring points; colour indicates synthetic feasibility, red=low, and green=high. Amino acids crucial for receptor activation and potency are highlighted in light green.

Uncaging mechanism

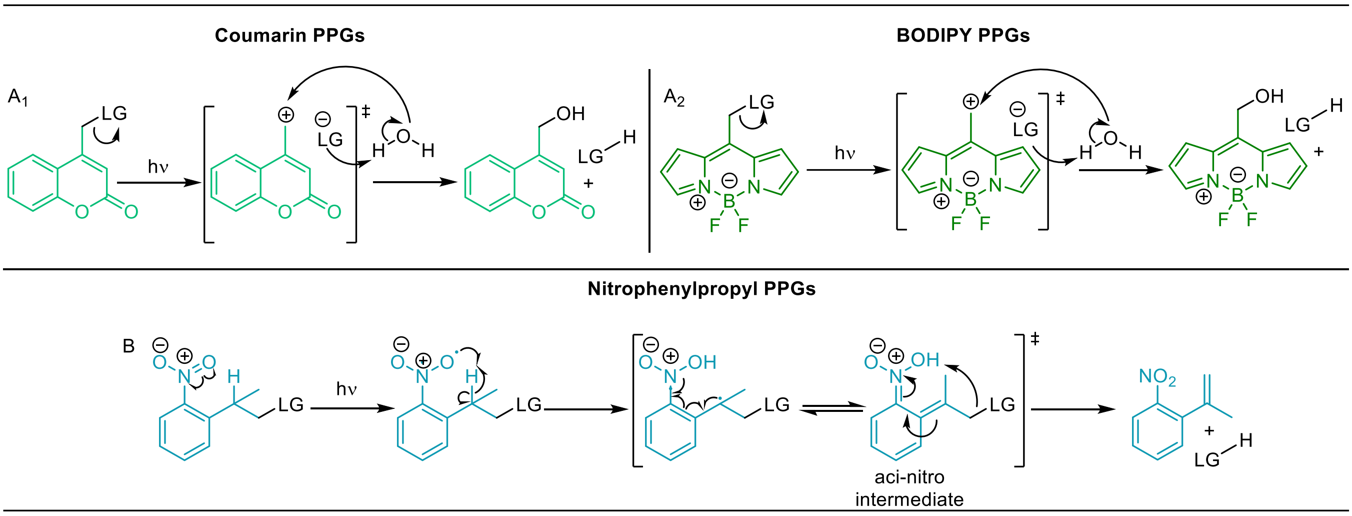

Figure S2. Overview of uncaging mechanisms. $A_{1/2}$) Coumarin and BODIPY PPGs follow a photo S_N1 cleavage mechanism, resulting in a cationic transition state. Subsequently, a nucleophile from the solvent (in physiological systems, H_2O) attacks the electrophilic carbocation and regenerates the original PPG. B) Nitrophenyl propyl PPGs follow a bi-radical Norrish type II photocleavage. Irradiation leads to a biradical transition state, followed by γ-proton abstraction and the formation of a cyclic aci-nitro intermediate, resulting in the loss of aromaticity. Electron pair rearrangement reinstates aromaticity and simultaneously expels the cargo. The spent photocage is thereby transformed into a nitro-styrene derivative.

Uncaging in 30%ACN/ddH$_2$O

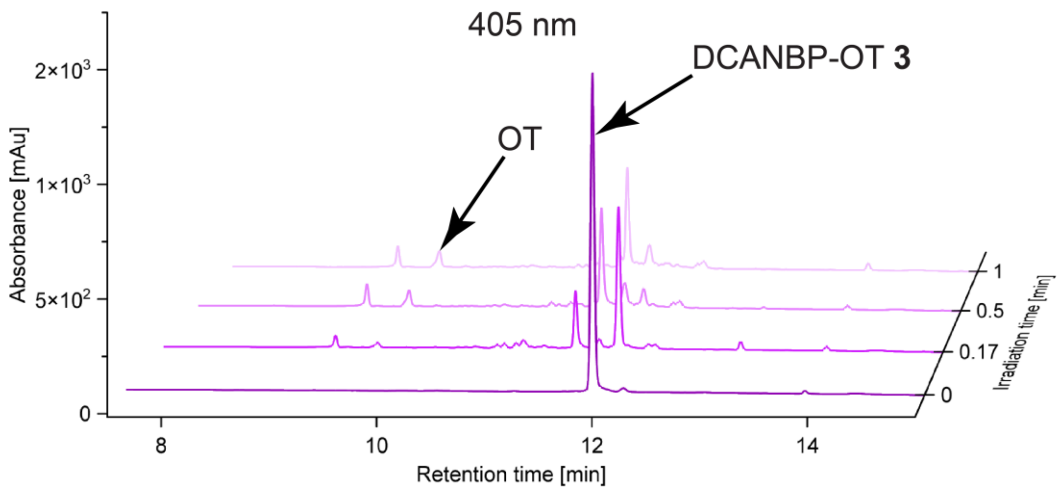

Figure S3. Uncaging in ACN. 50 µM solution of DCANBP-OT **3** in 30% ACN/ddH$_2$O was irradiated with a 405 nm LED. Uncaging was quantitative after 1 min.

Dark stability

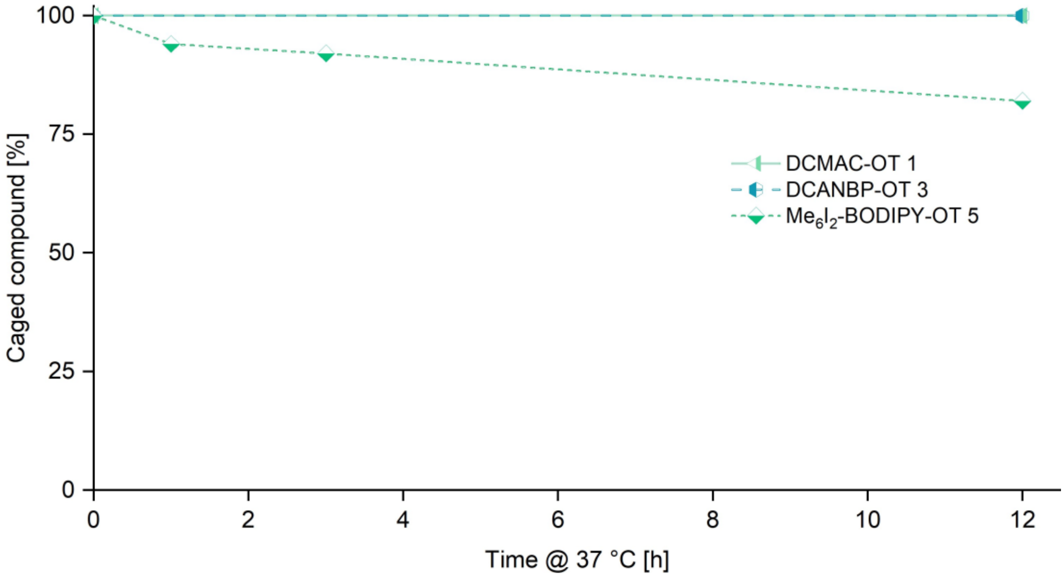

Figure S4. Dark stability of the caged OT derivatives. 50 µM solutions of compounds **1**, **3** or **5** in PBS were incubated at 37 °C and HPLC samples were measured (25 µL injection, 5% B to 95% B in 27 min; eluents A: 0.1%TFA in ddH$_2$O, eluent B: 0.08% TFA in 90% ACN/ddH$_2$O) to determine the stability of the derivatives at physiological pH. Due to the acid sensitivity of the BODIPY core structure, measurements for compound **5** were done without acid additives in the eluents. Peaks were integrated, and hydrolytic decomposition was plotted as a percentage of the remaining caged derivatives. DCMAC-OT **1** and DCANBP-OT **3** remained fully stable for at least 12 h, whereas Me$_6$I$_2$-BODIPY-OT **5** showed 18% hydrolytic degradation over the tested period.

BODIPY-amine

During reaction optimisation, we observed that higher temperatures (RT → 40 °C) resulted in multiple peaks without the desired product mass. Additionally, we observed that stronger bases, such as DIPEA or Et_3N, increased reaction rates but exclusively led to compounds with a BODIPY attached *via* an amine linkage rather than a carbamate (**Figure S51**). When uncaging these BODIPY-amine derivatives, we observed masses corresponding to disulfide reduction with the BODIPY cage still attached (**Figure S5**).

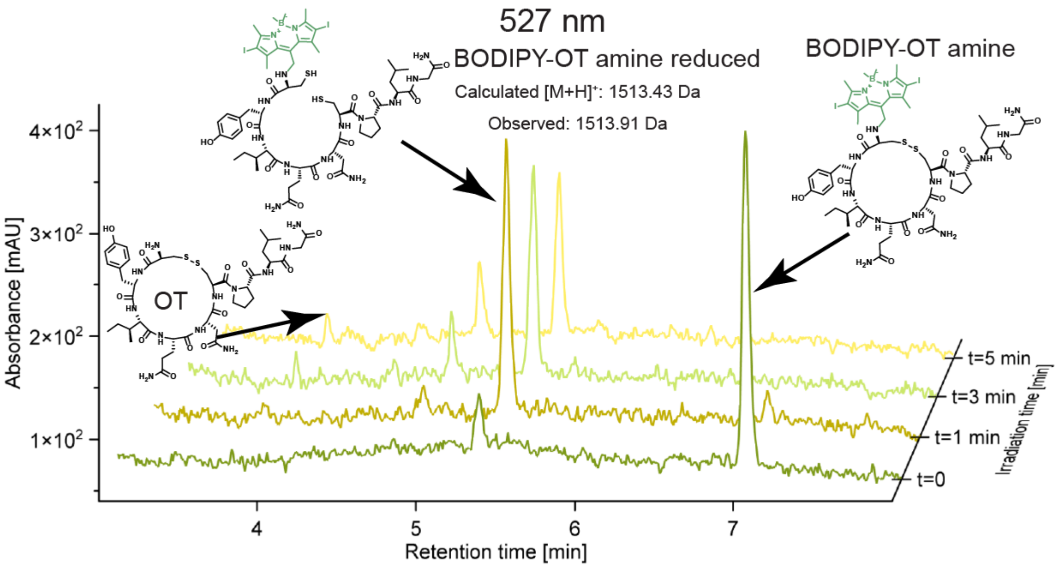

Figure S5. Uncaging of BODIPY-amine in ACN. 50 µM solution of BODIPY-OT-amine in 30% ACN/ddH_2O was irradiated for 1, 3, and 5 min with a 527 nm LED. Uncaging produced a peak with a mass corresponding to a reduced version with the BODIPY cage still attached as an amine.

IP1 assays

Table S2. Results of cellular functional IP_1-FRET assay on HEK-293 cells overexpressing the GFP-tagged human neuropeptide receptor of interest.

**Receptor**	**hOTR**				**hV1aR**				**hV1bR**			
**Compound**	**OT**	**1**	**3**	**5**	**VP**	**2**	**4**	**6**	**VP**	**2**	**4**	**6**
**logEC_{50}**	-8.151	-5.969	-4.554	-7.317	-9.006	-6.123	-5.475	-8.500	-8.101	-5.348	-4.767	-7.168
**± SEM [M]**	0.048	0.055	0.172	0.080	0.037	0.046	0.043	0.064	0.074	0.130	0.094	0.126
**EC_{50} [nM]**	7.1	1,074	27,940	48.2	0.9	754	3,350	3.2	7.9	4,485	17,110	68.0
**ΔEC_{50} [fold]**	/	>150x	>3,900x	7x	/	>750x	>3,300x	3x	/	>560x	>2,130x	8.5x

ΔEC_{50} is calculated by dividing the reference EC_{50}.

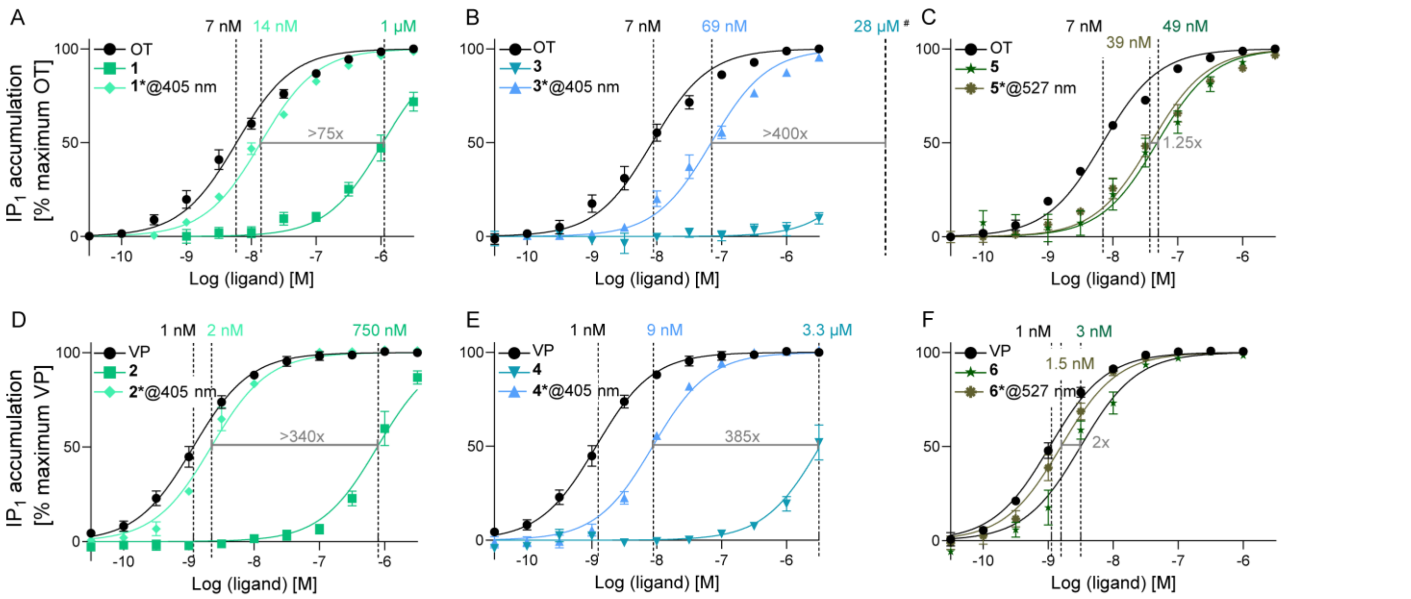

Figure S6. Pharmacological evaluation of *in situ* uncaged neuropeptides at human OTR and $V_{1a}R$. Cellular functional IP_1 assays were performed on HEK-293 cells stably overexpressing hOTR or $hV_{1a}R$. Oxytocin and vasopressin derivatives *in situ* uncaged for 60 s (**1-4**: 405 nm; **5**, **6**: 527 nm). Each point represents at least three independent measurements with technical triplicates. Results were normalised to OT/VP (100%) and negative control (vehicle; 0%) activity. Error bars indicate the standard error of the mean (SEM). (A) **1** at hOTR, (B) **3** at hOTR, (C) **5** at hOTR, (D) **2** at $hV_{1a}R$, (E) **4** at $hV_{1a}R$, (F) **6** at $hV_{1a}R$. EC_{50} values are shown by dashed vertical lines and presented above with the same colour coding for the corresponding compounds; asterisks indicate extrapolated values, and numbers on horizontal lines indicate an x-fold decrease of EC_{50} values after pre-uncaging.

Table S3. Results of cellular functional IP_1-FRET assay on HEK-293 cells overexpressing the GFP-tagged human neuropeptide receptor of interest with *in situ* uncaged probes.

**Receptor**	**hOTR**			**hV1aR**		
**Compound**	**1***	**3***	**5***	**2***	**4***	**6***
**logEC50**	-7.852	-7.160	-7.413	-8.660	-8.060	-8.802
**± SEM [M]**	0.030	0.047	0.055	0.038	0.033	0.043
**EC50 [nM]**	14.1	69.2	38.6	2.2	8.7	1.58
**ΔEC50 fold]**	76x	404x	1.25x	343x	385x	2x

ΔEC_{50} is calculated by dividing the caged EC_{50} by the pre-uncaged EC_{50}.

Confocal Microscopy

HEK-293 cells

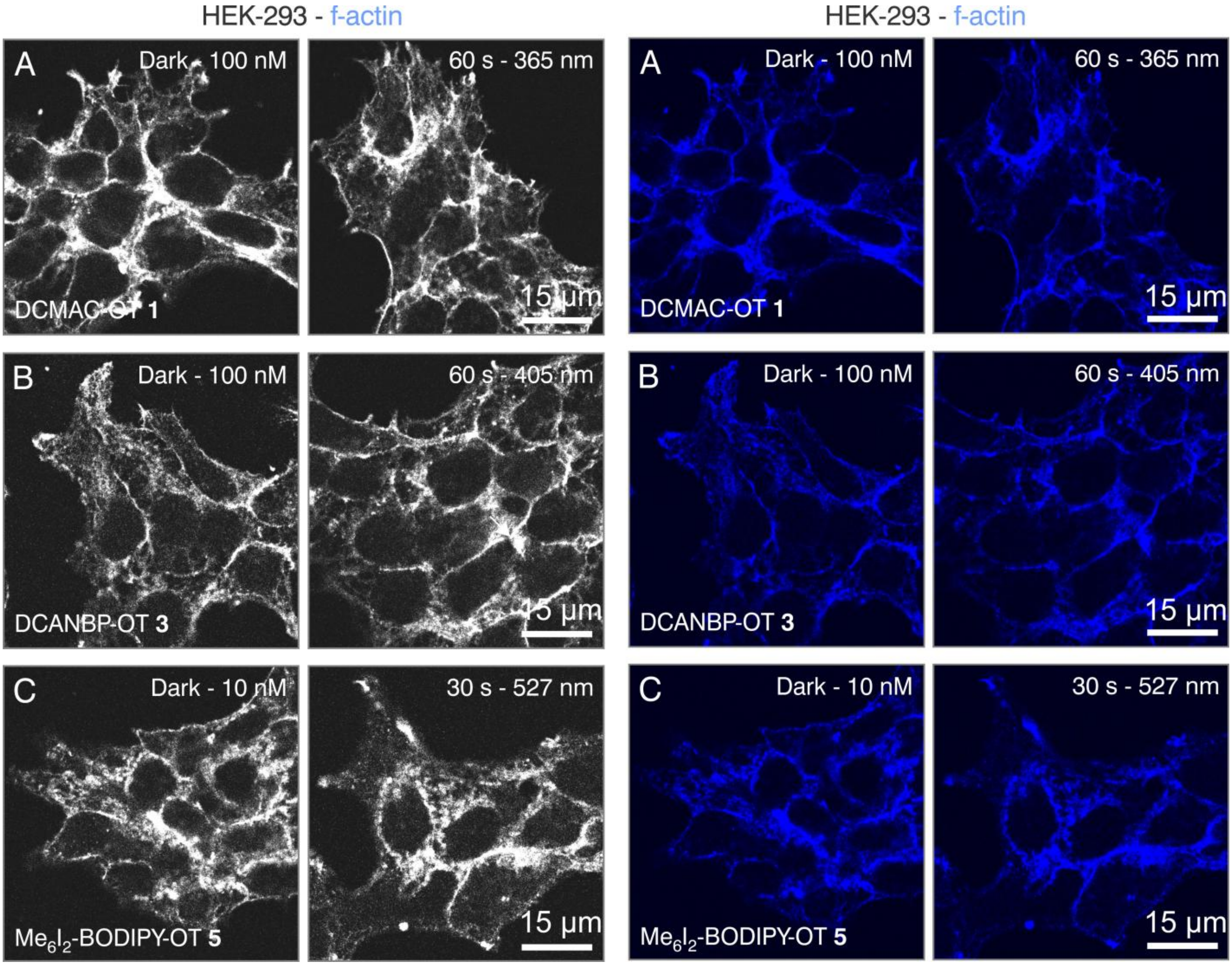

Figure S7. *In vitro* evaluation of the biological activity of OT photoprobes, caged and uncaged, on HEK-293 cells overexpressing hOTR-GFP. f-actin channel left grey, right blue.

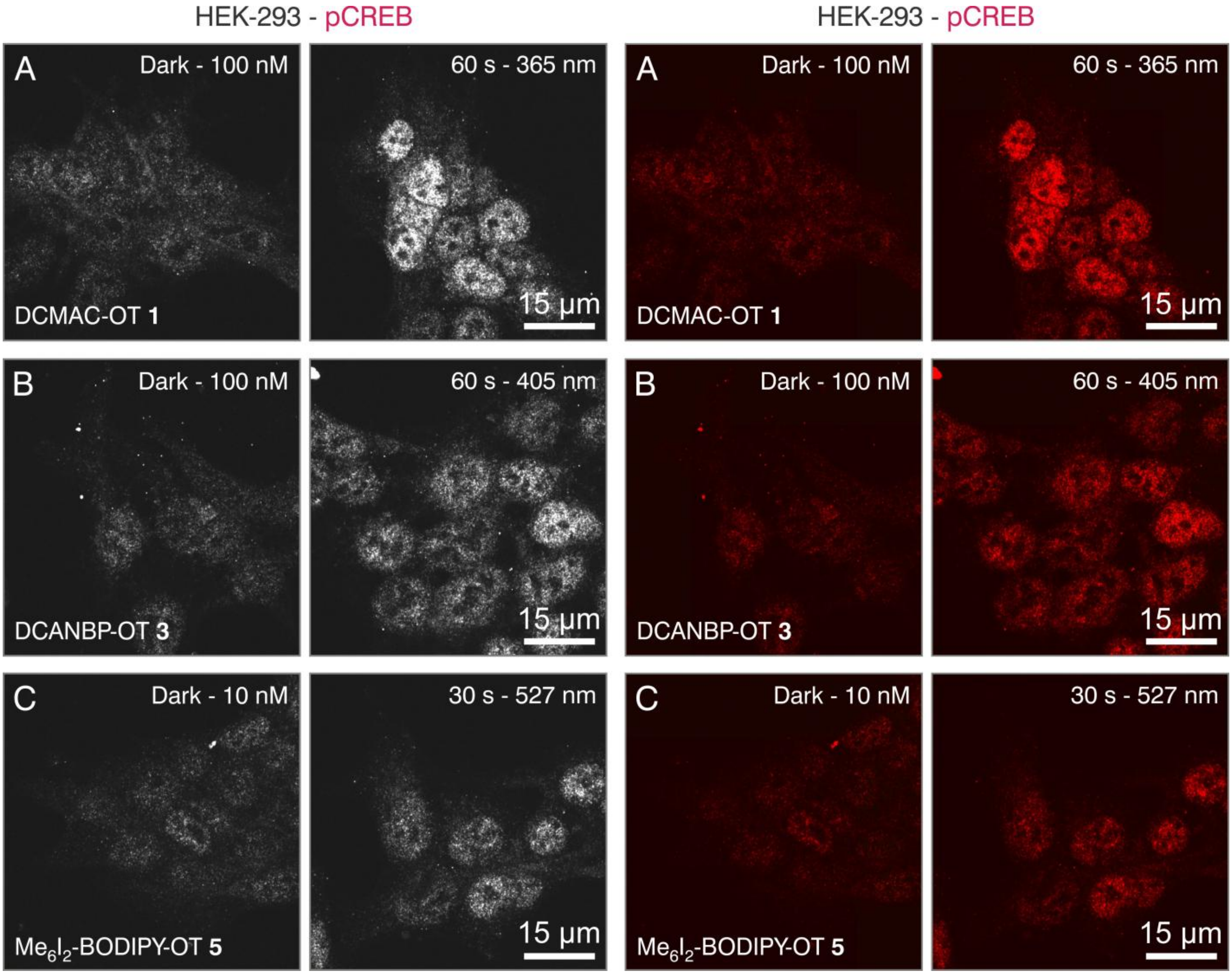

Figure S8. *In vitro* evaluation of the biological activity of OT photoprobes, caged and uncaged, on HEK-293 cells overexpressing hOTR-GFP. pCREB channel left grey, right red.

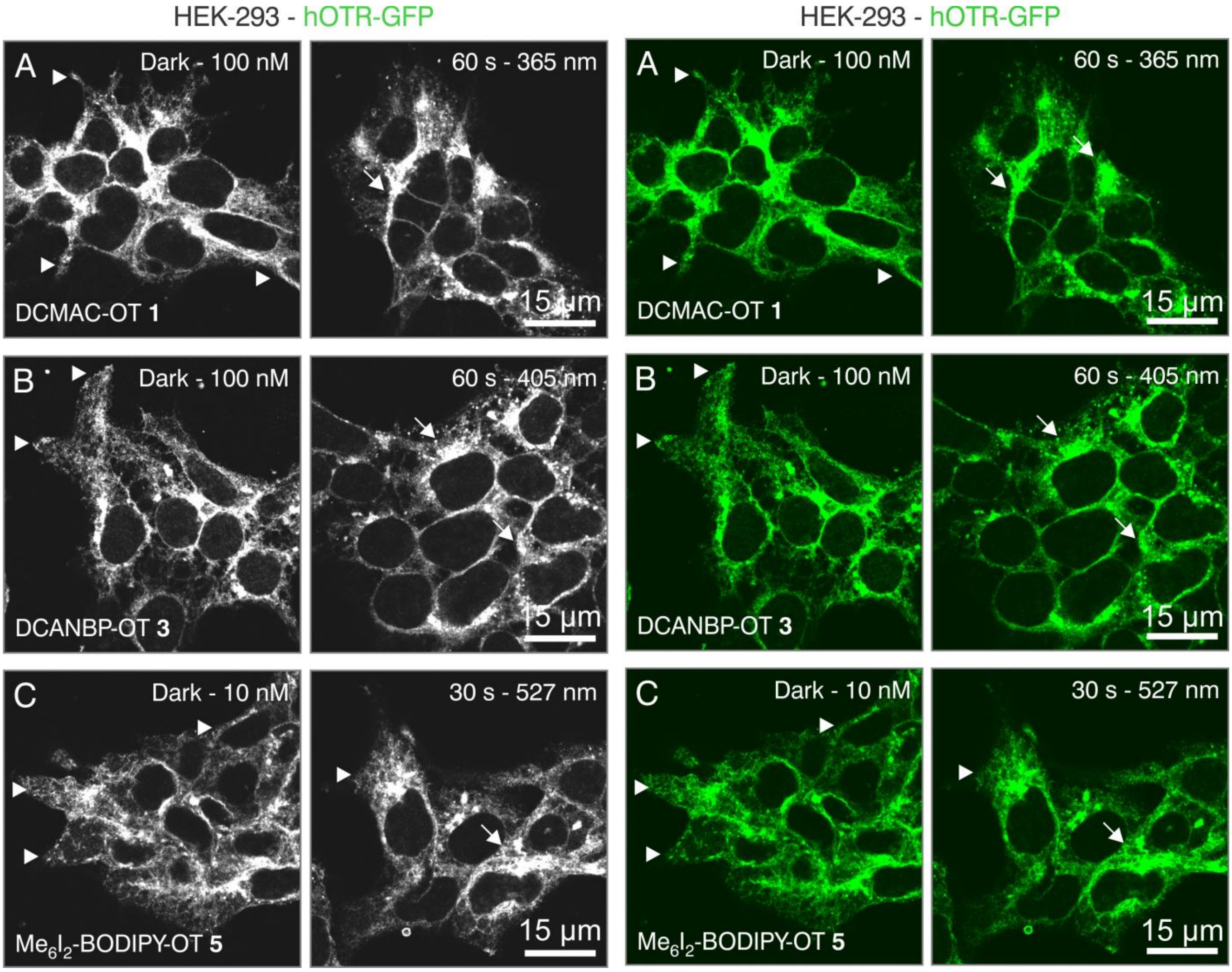

Figure S9. *In vitro* evaluation of the biological activity of OT photoprobes, caged and uncaged, on HEK-293 cells overexpressing hOTR-GFP. GFP channel left grey, right green. Arrowheads indicate regions with receptors on cell membranes, arrows indicate areas with receptor internalisation.

Primary neurons

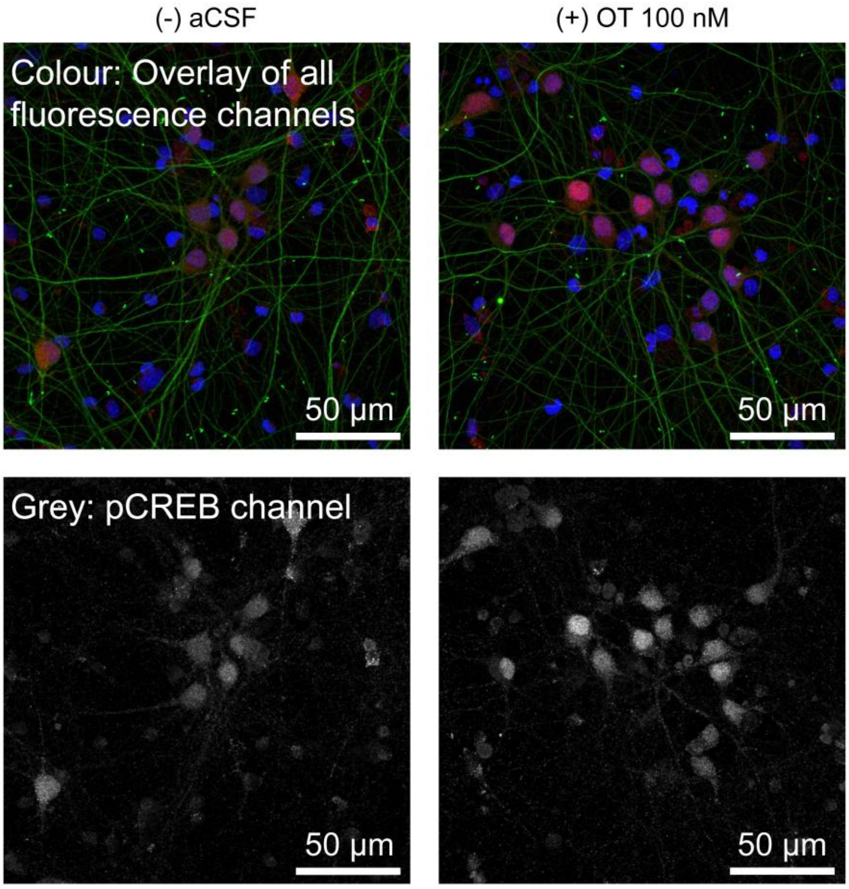

Figure S10. *In vitro* evaluation of the biological activity of OT photoprobes: (-) Negative control (aCSF) and (+) positive control (100 nM OT): Nuclei were stained with DAPI (blue), and dendrites with antibodies for MAP2 (green). Phosphorylation of CREB (red) was quantified after immunocytochemistry of n=~100 cells over two coverslips and normalised to the negative control (aCSF).

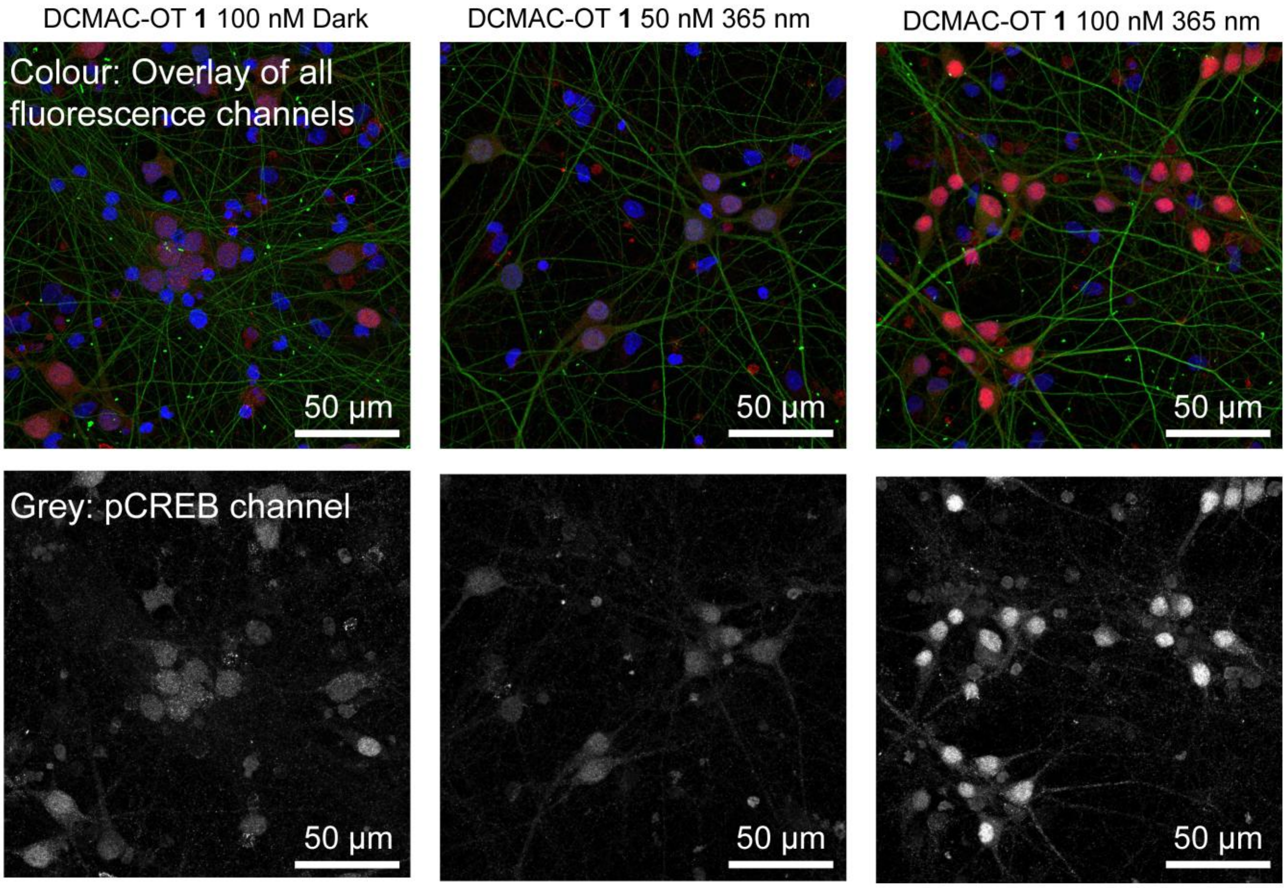

Figure S11. *In vitro* evaluation of the biological activity of OT photoprobes, caged and uncaged, on primary rat hippocampal neurons. DCMAC-OT **1** Nuclei were stained with DAPI (blue), and dendrites with antibodies for MAP2 (green). Phosphorylation of CREB (red) was quantified after immunocytochemistry of n=~100 cells over two coverslips and normalised to the negative control (aCSF). (-) Negative control (aCSF) and (+) positive control (OT, 100 nM), DCMAC-OT **1**, 100 nM, dark and irradiated at 365 nm for 60 s.; grey, separated pCREB channel.

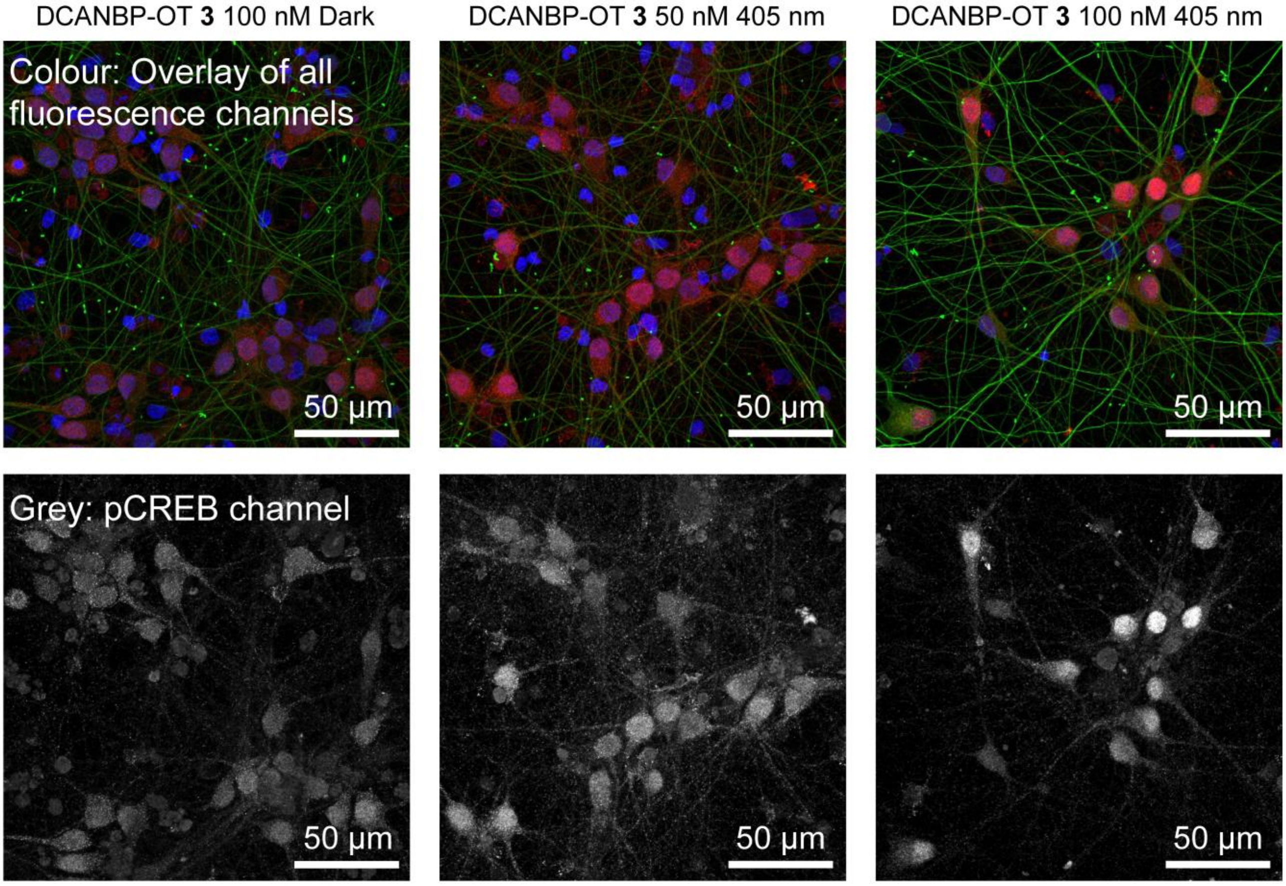

Figure S12. *In vitro* evaluation of the biological activity of OT photoprobes, caged and uncaged, on primary rat hippocampal neurons. DCANBP-OT **3** Nuclei were stained with DAPI (blue), and dendrites with antibodies for MAP2 (green). Phosphorylation of CREB (red) was quantified after immunocytochemistry of n=~100 cells over two coverslips and normalised to the negative control (aCSF). (-) Negative control (aCSF) and (+) positive control (OT, 100 nM), DCANBP-OT **3**, 100 nM, dark and irradiated at 405 nm for 60 s; grey, separated pCREB channel.

NMR spectra of compounds 7-17a

*t*Bu2-DCMAC 7

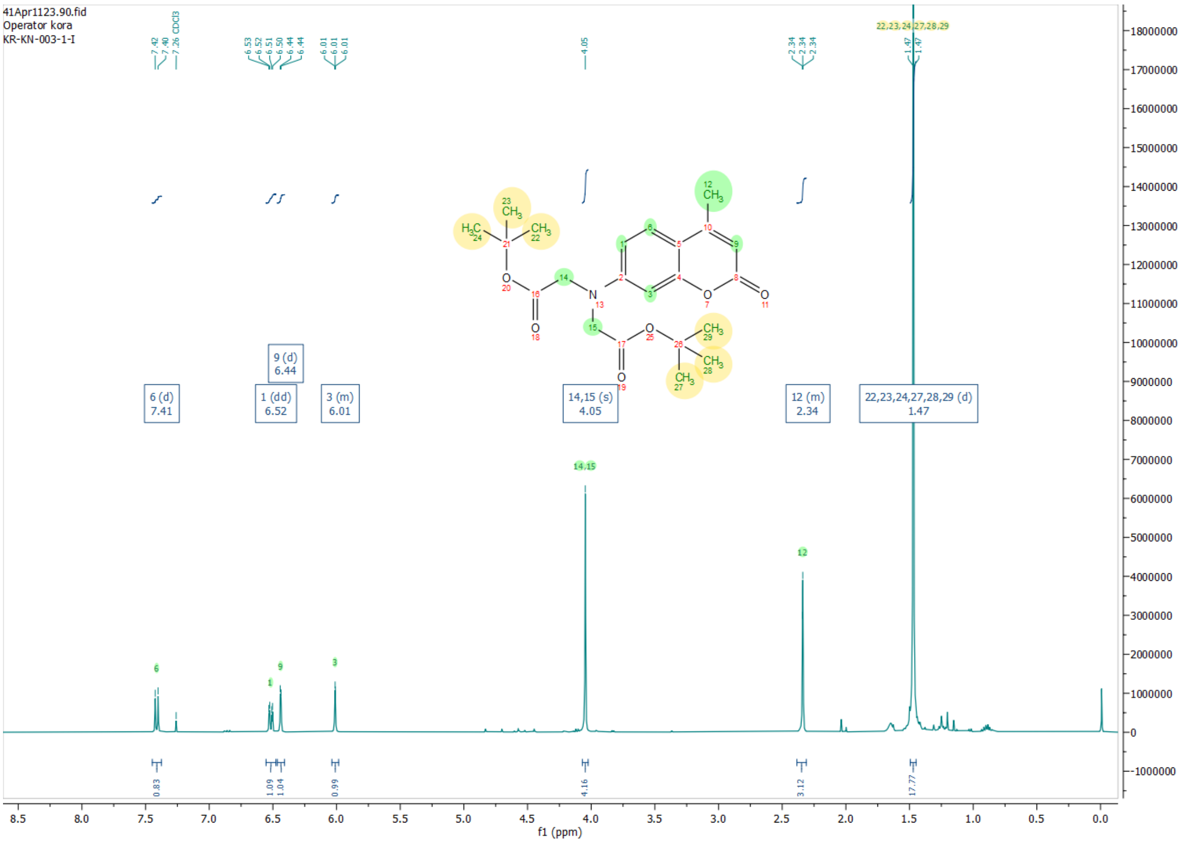

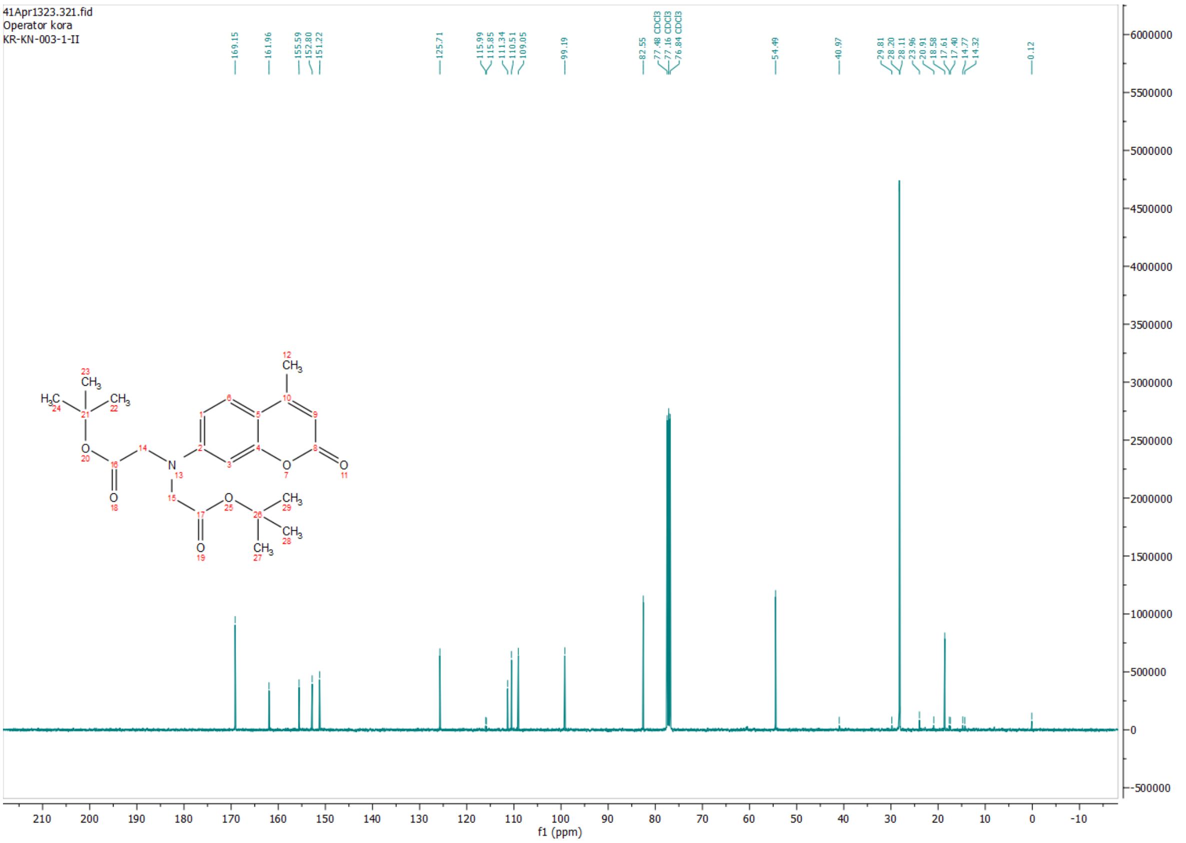

***t*Bu₂-DCMAC-CHO 8**

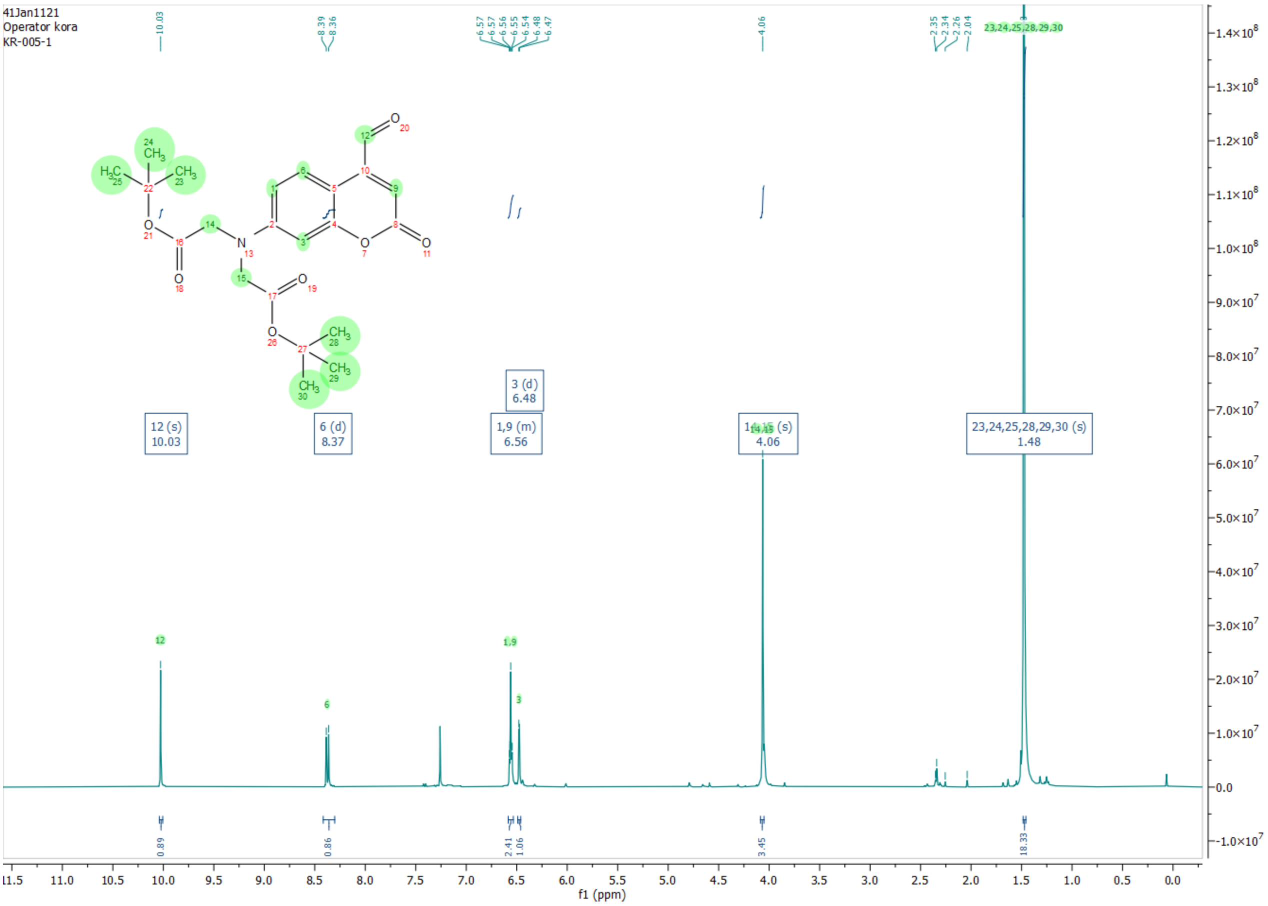

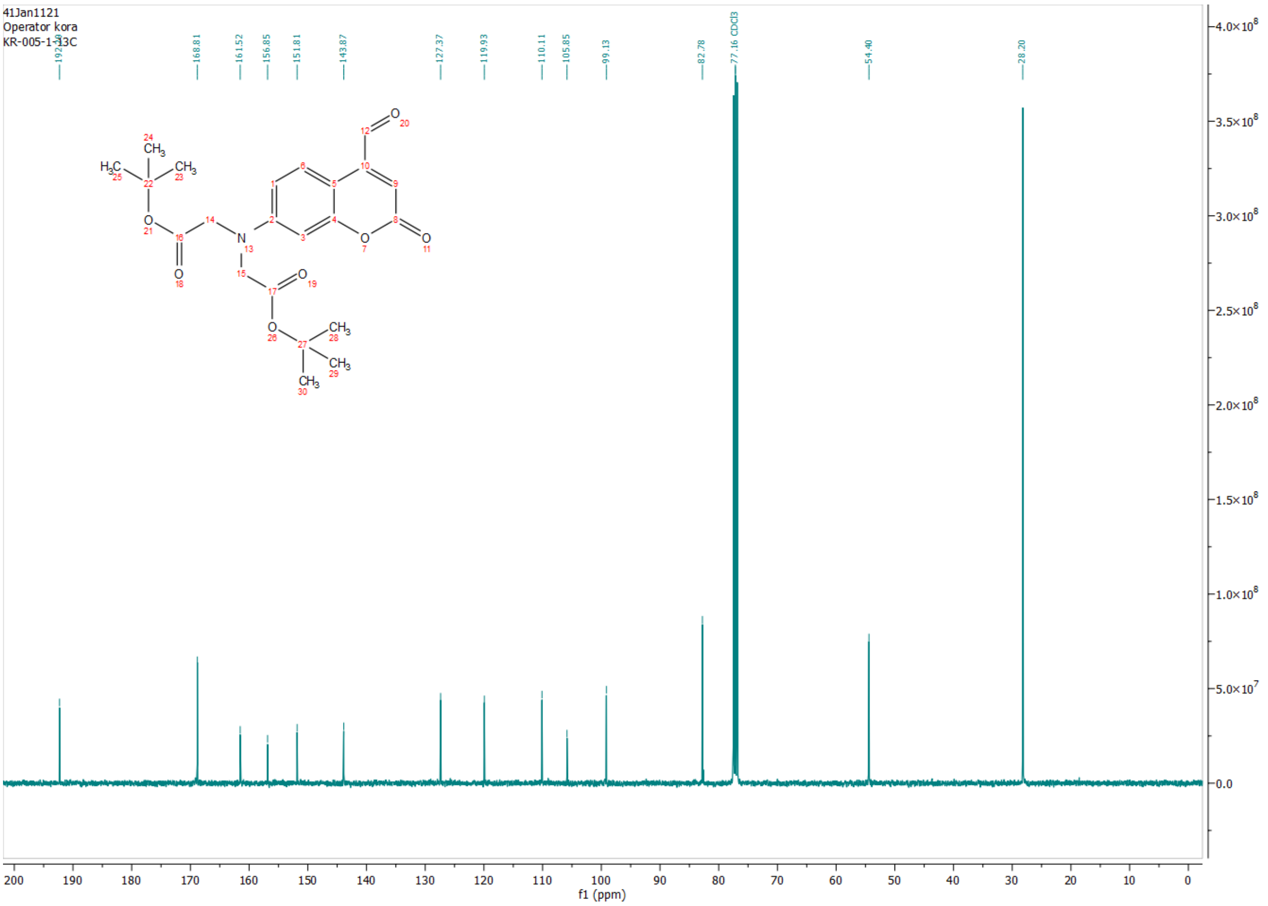

***t*Bu₂-DCMAC-OH 9**

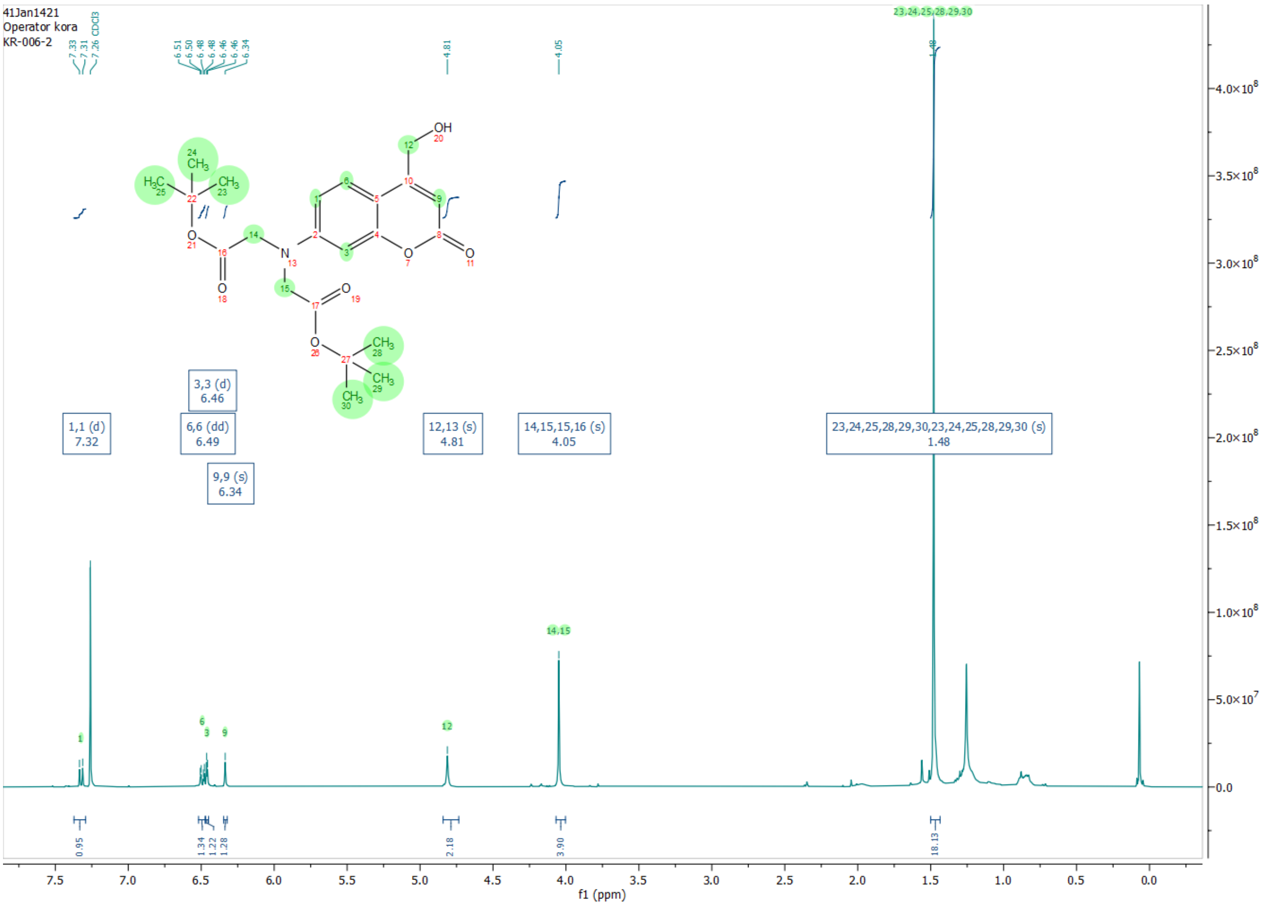

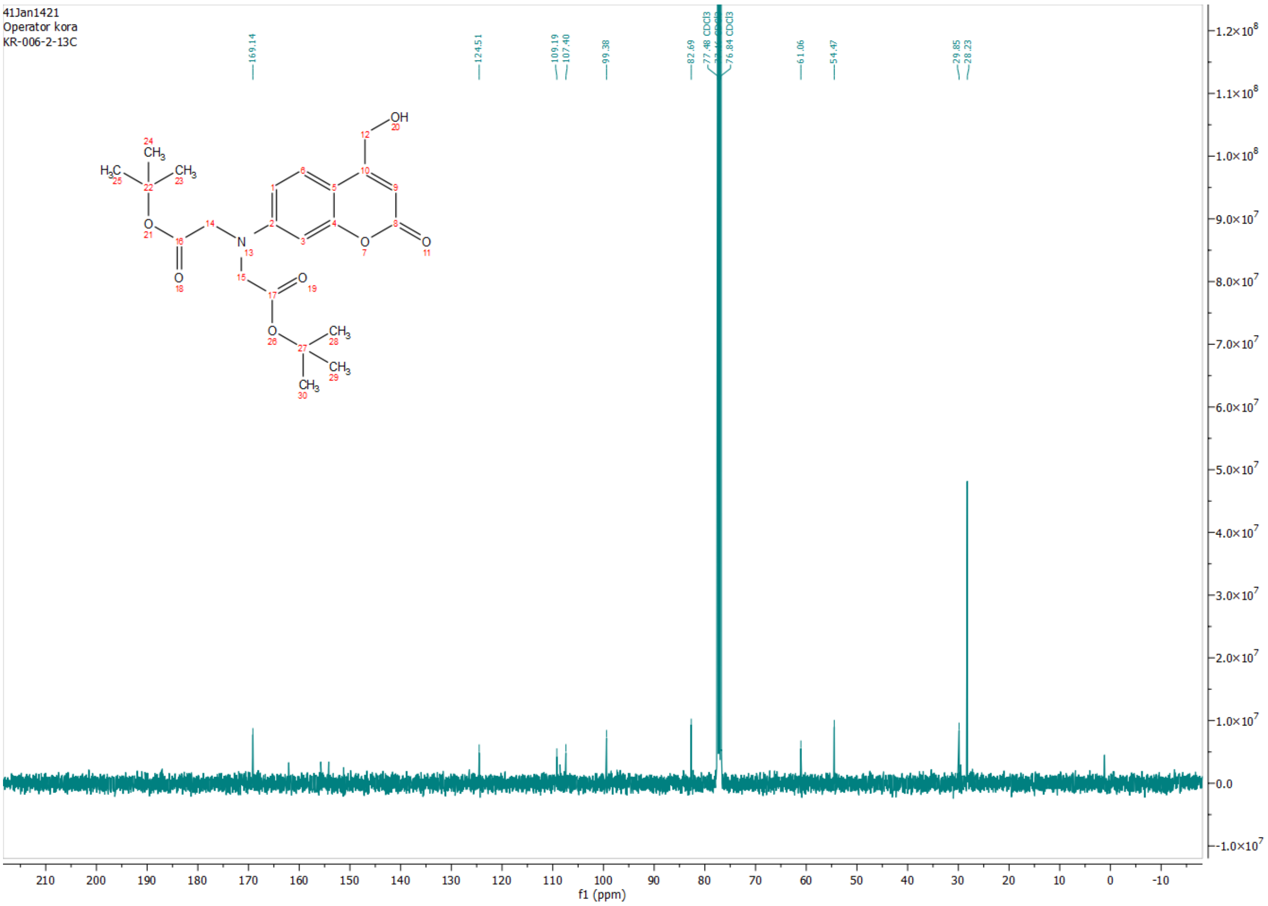

tBu₂-DCMAC-O-Carbonate 9a

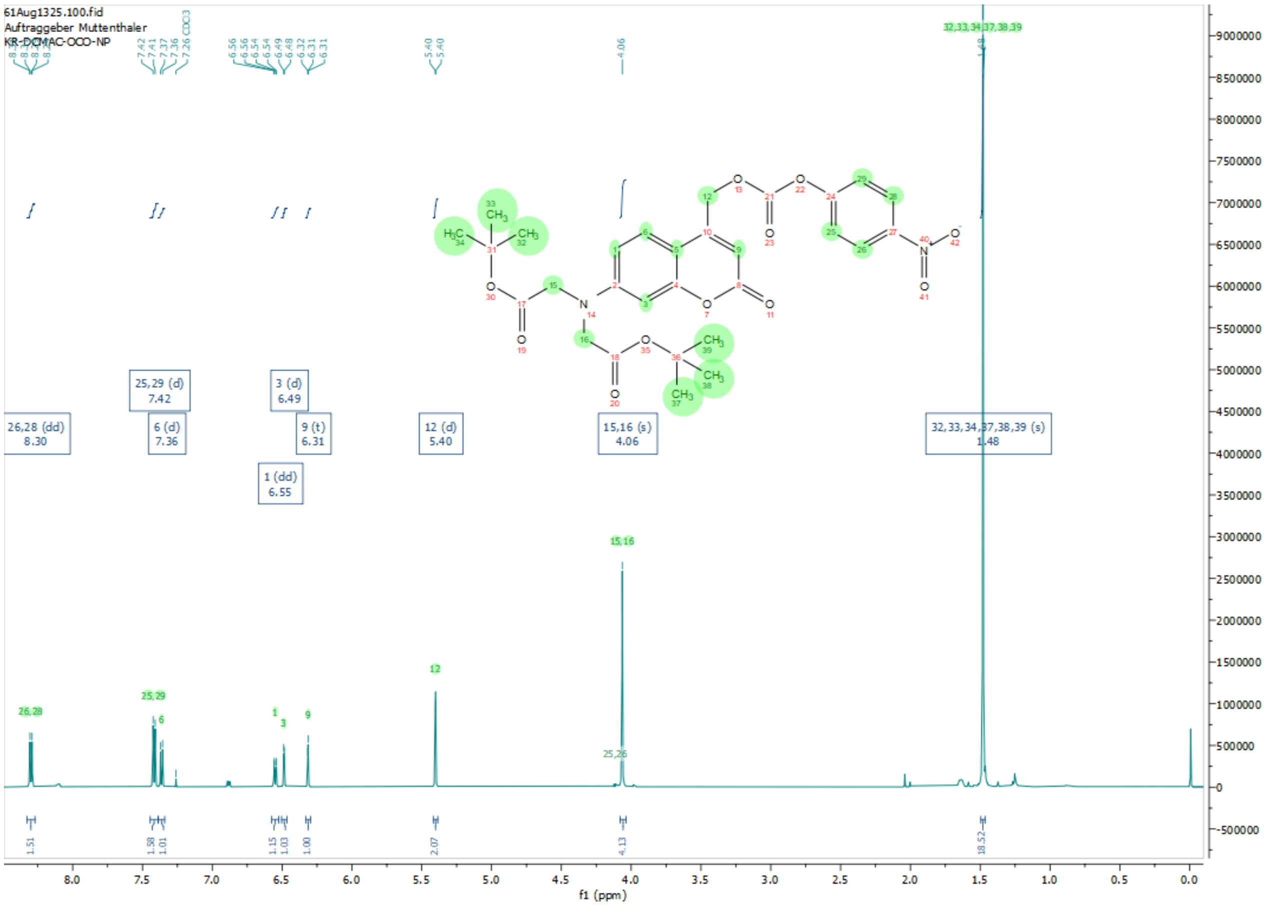

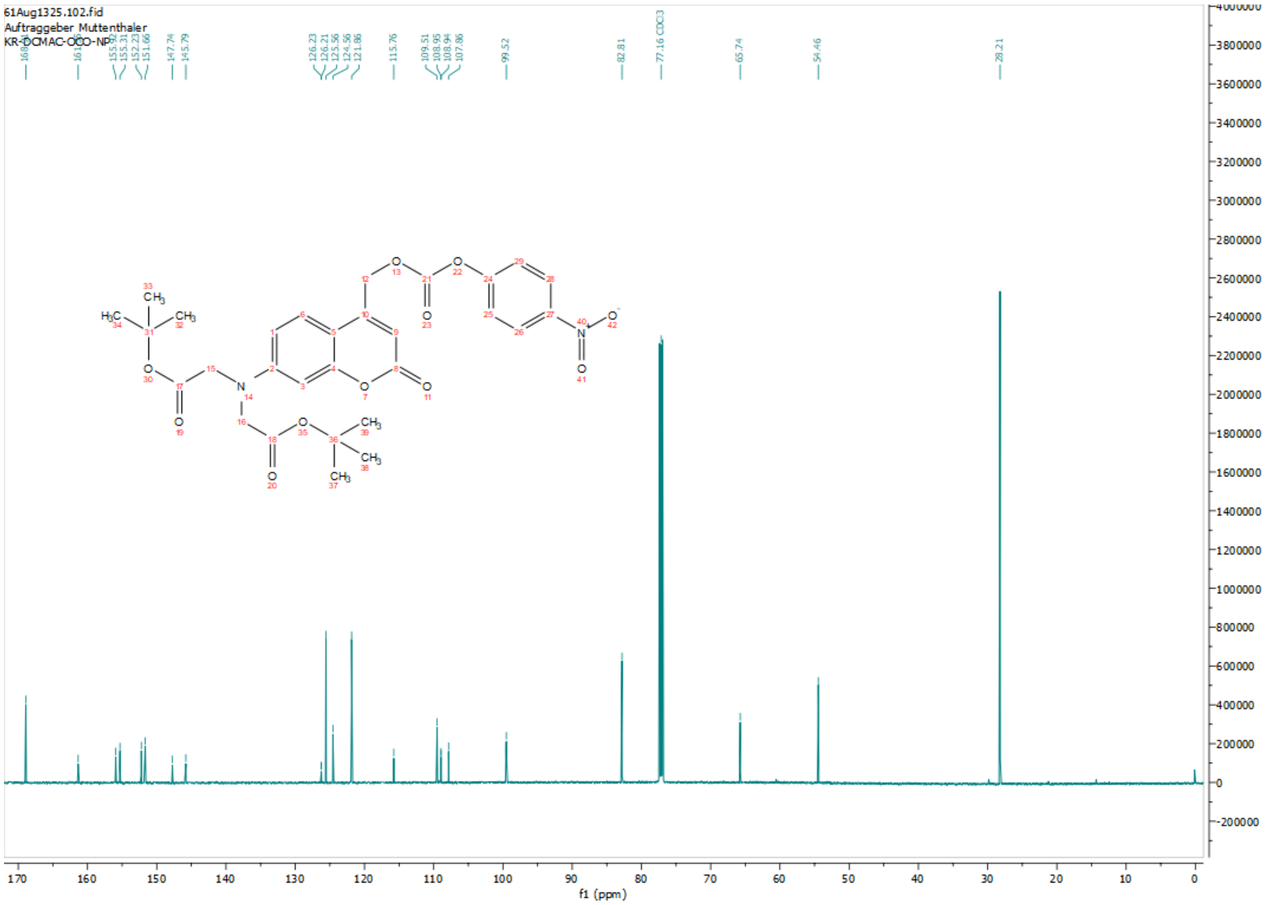

***tert*-Butyl 2-(5-bromo-2-nitrophenyl) acetate 10**

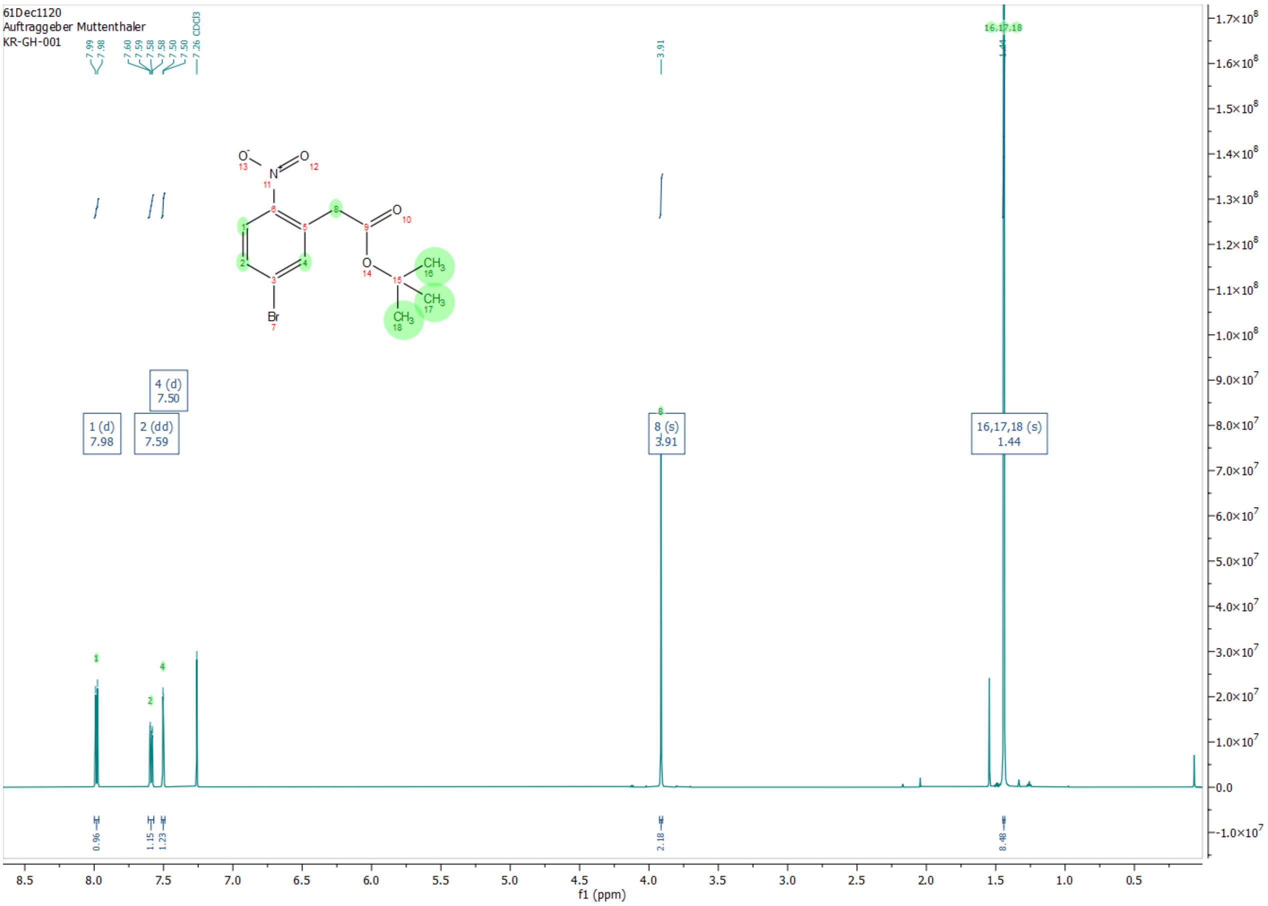

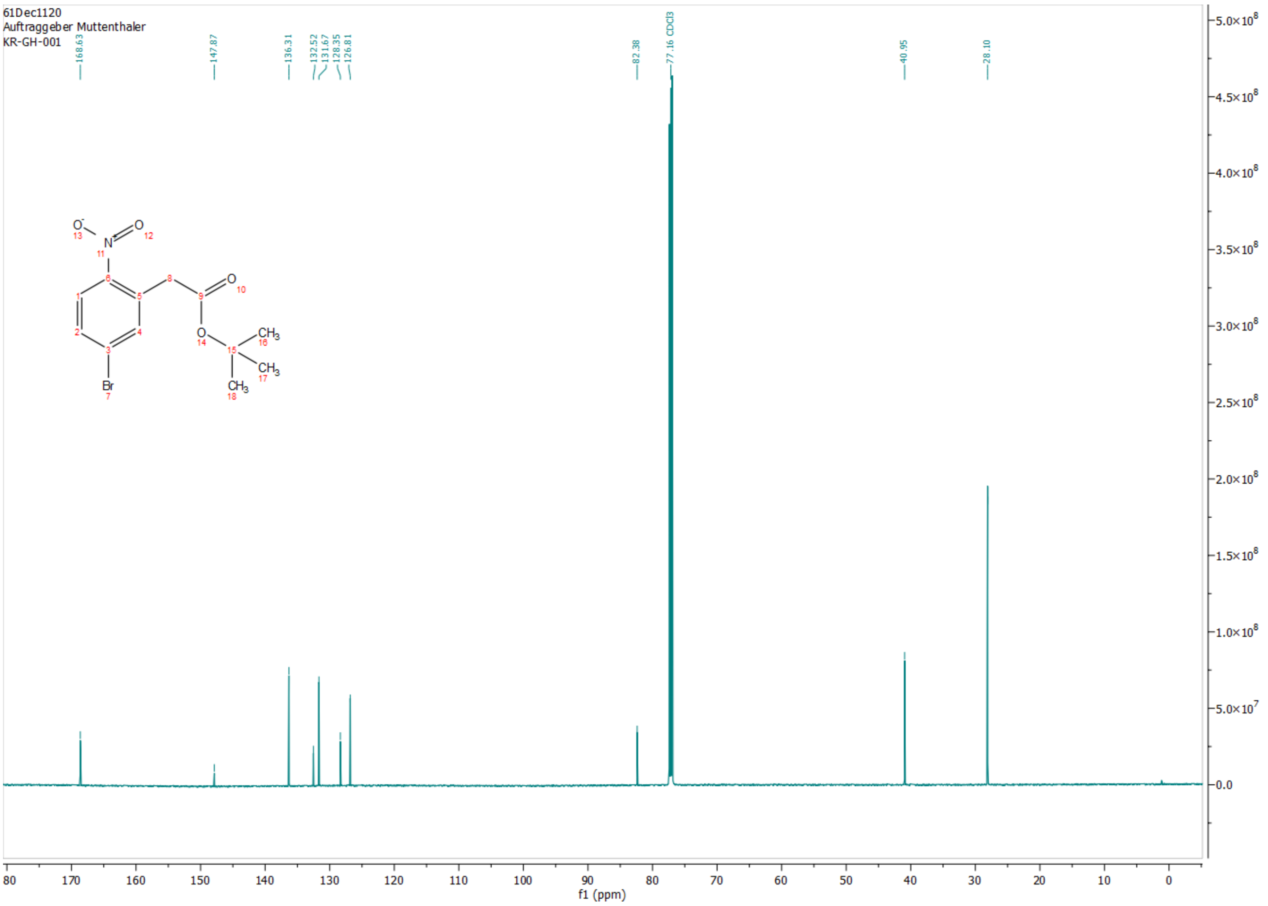

tert-Butyl 2-(5-bromo-2-nitrophenyl)propanoate 11

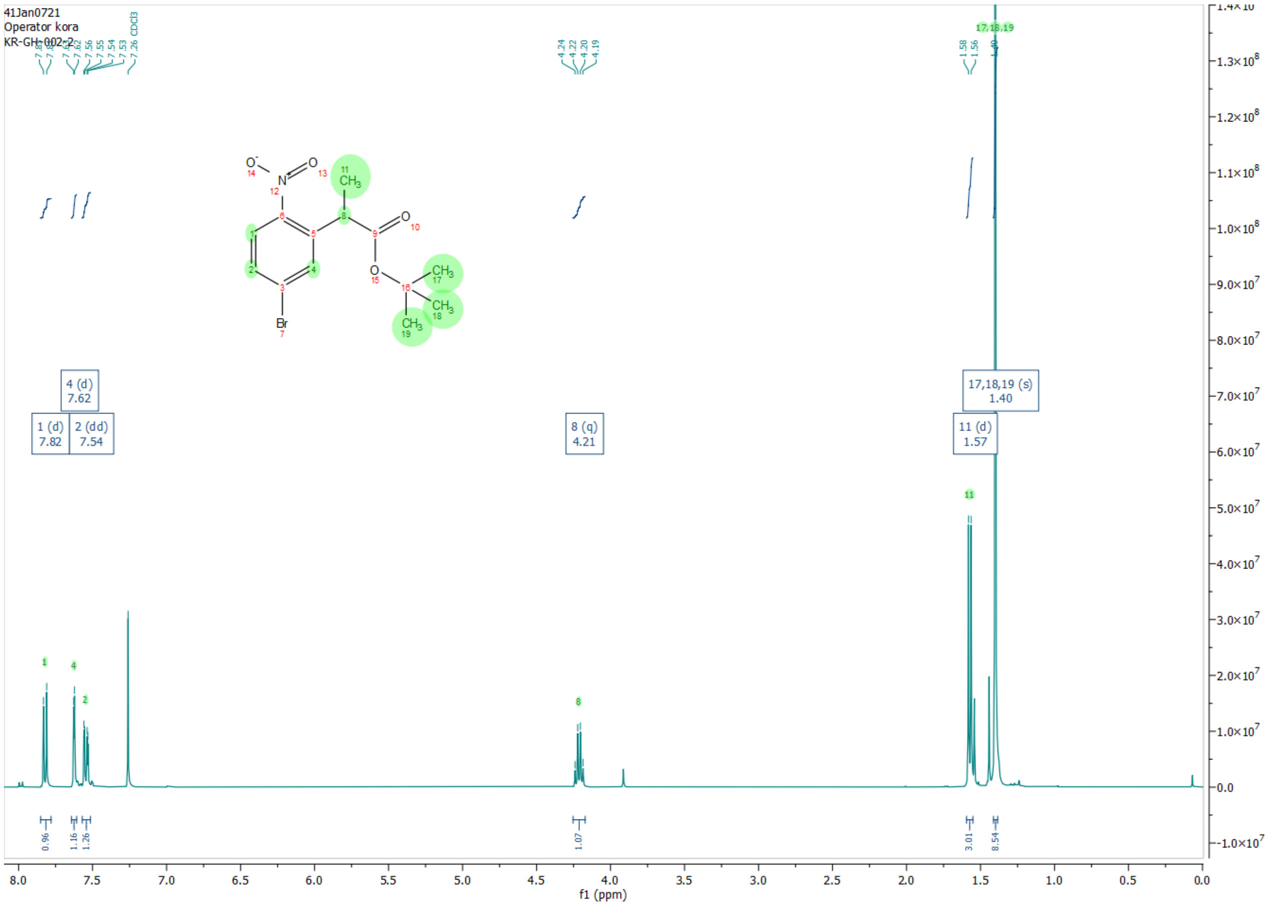

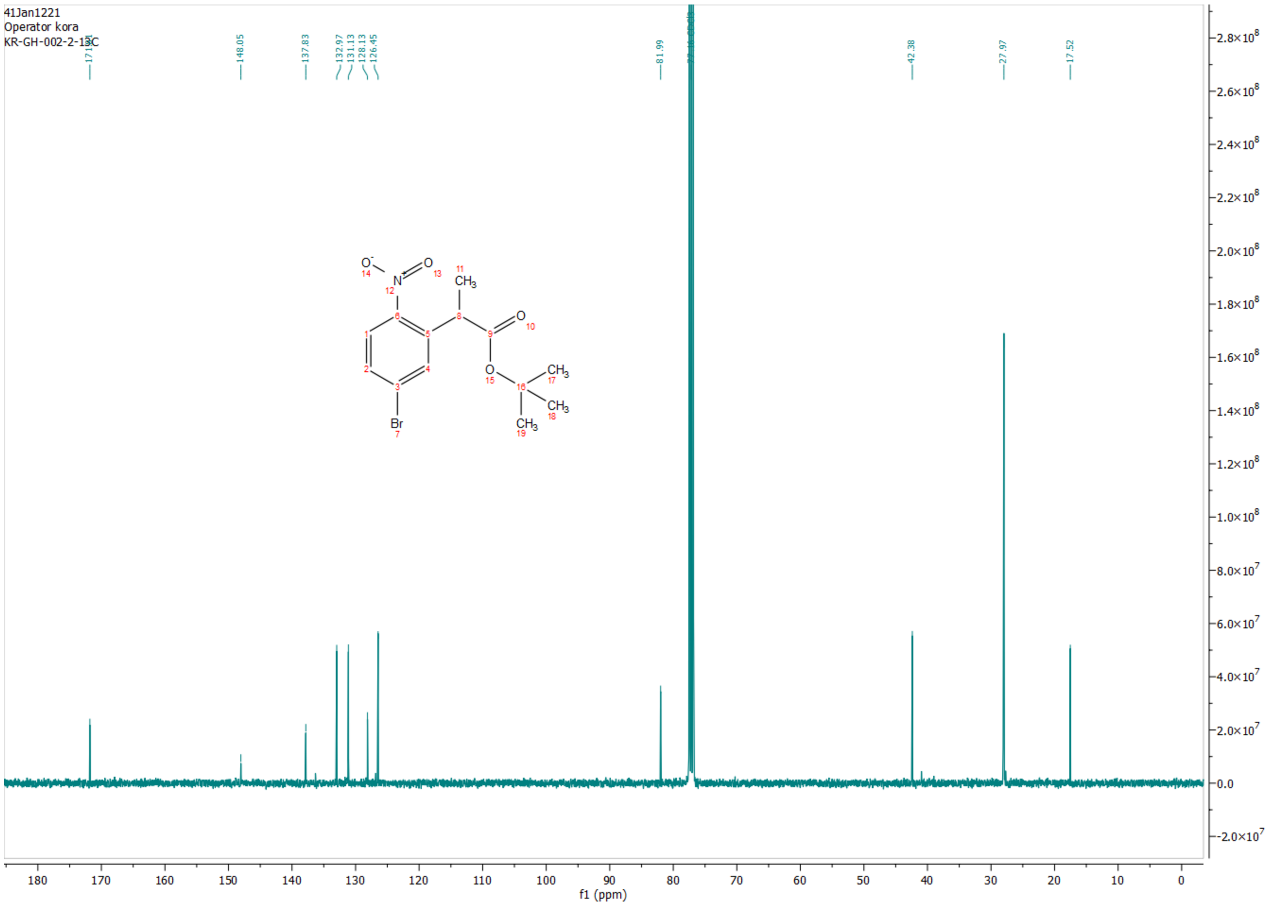

2-(5-Bromo-2-nitrophenyl)propan-1-ol 12

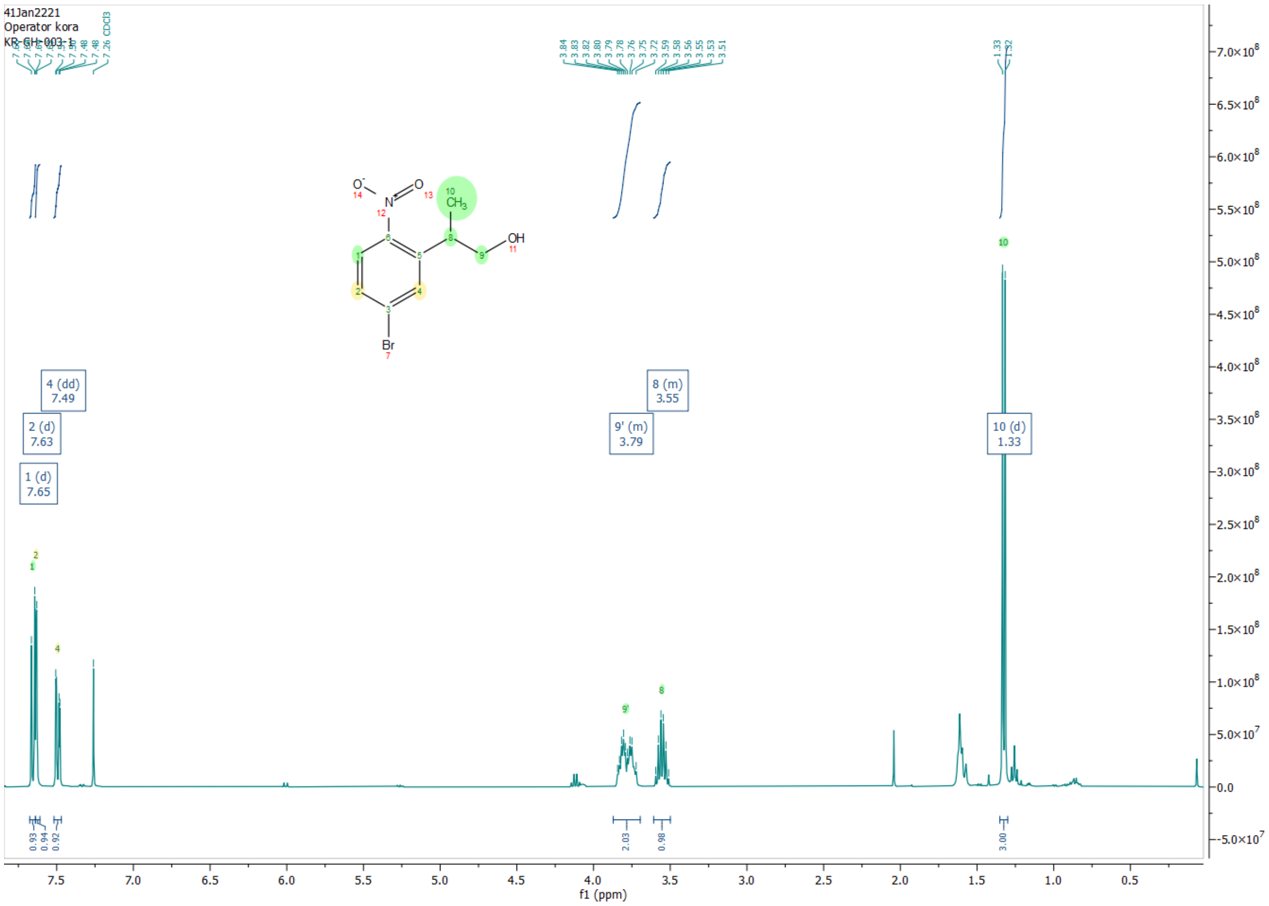

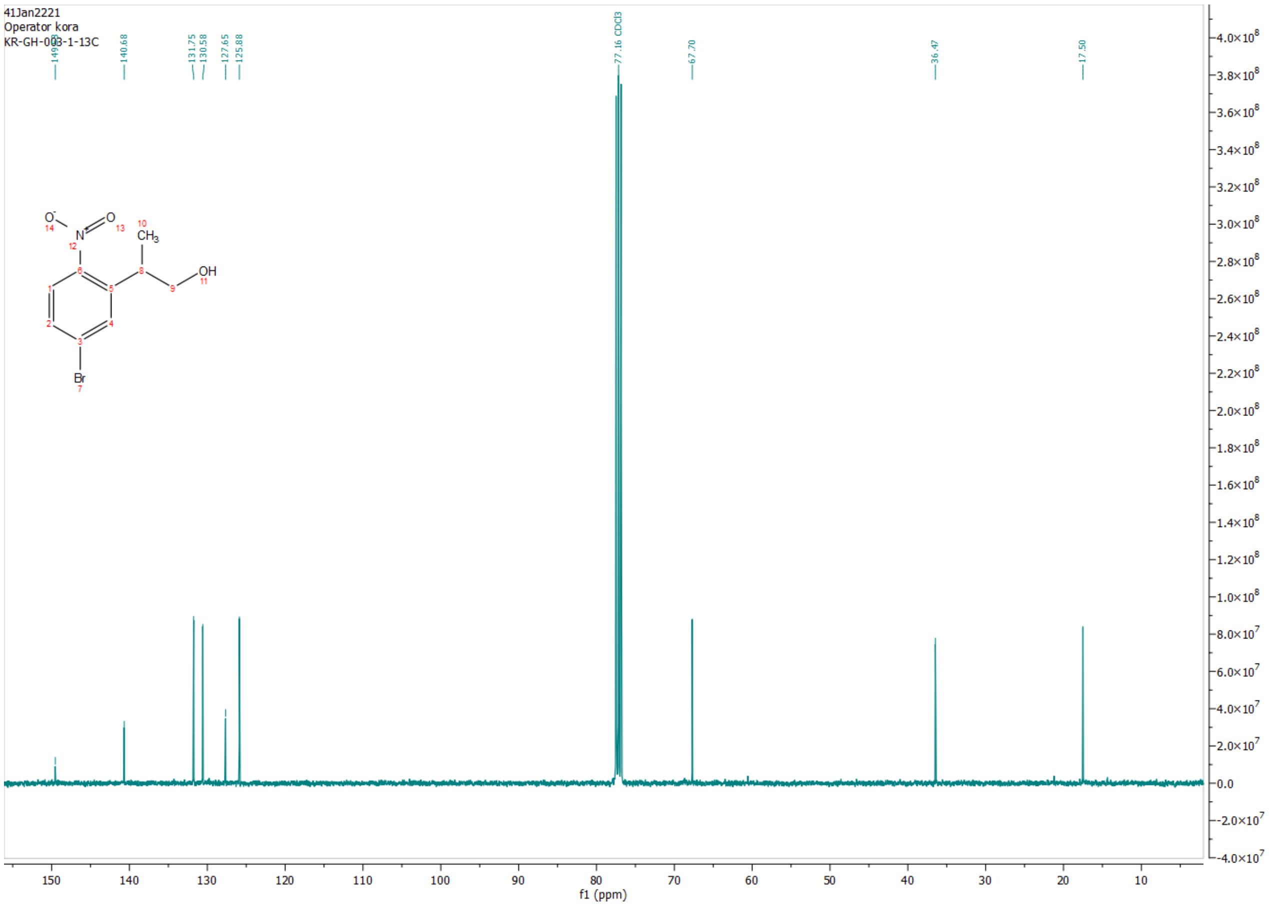

ANBP-OH 13

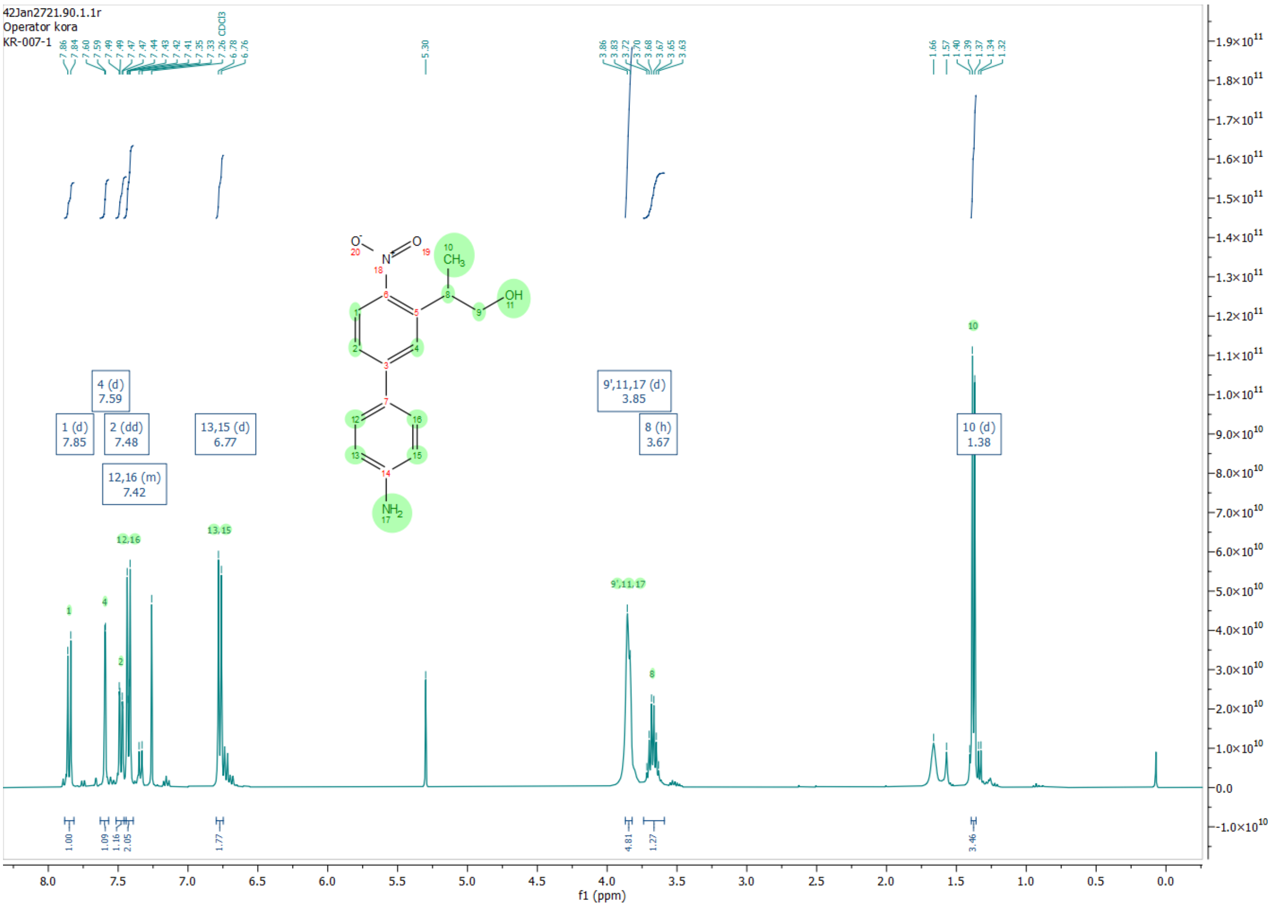

42Jan2721.90.1.1r
Operator kora
KR-007-1
1 (d)
7.85
4 (d)
7.59
2 (dd)
7.48
12,16 (m)
7.42
13,15 (d)
6.77
9',11,17 (d)
3.85
8 (h)
3.67
10 (d)
1.38
f1 (ppm)

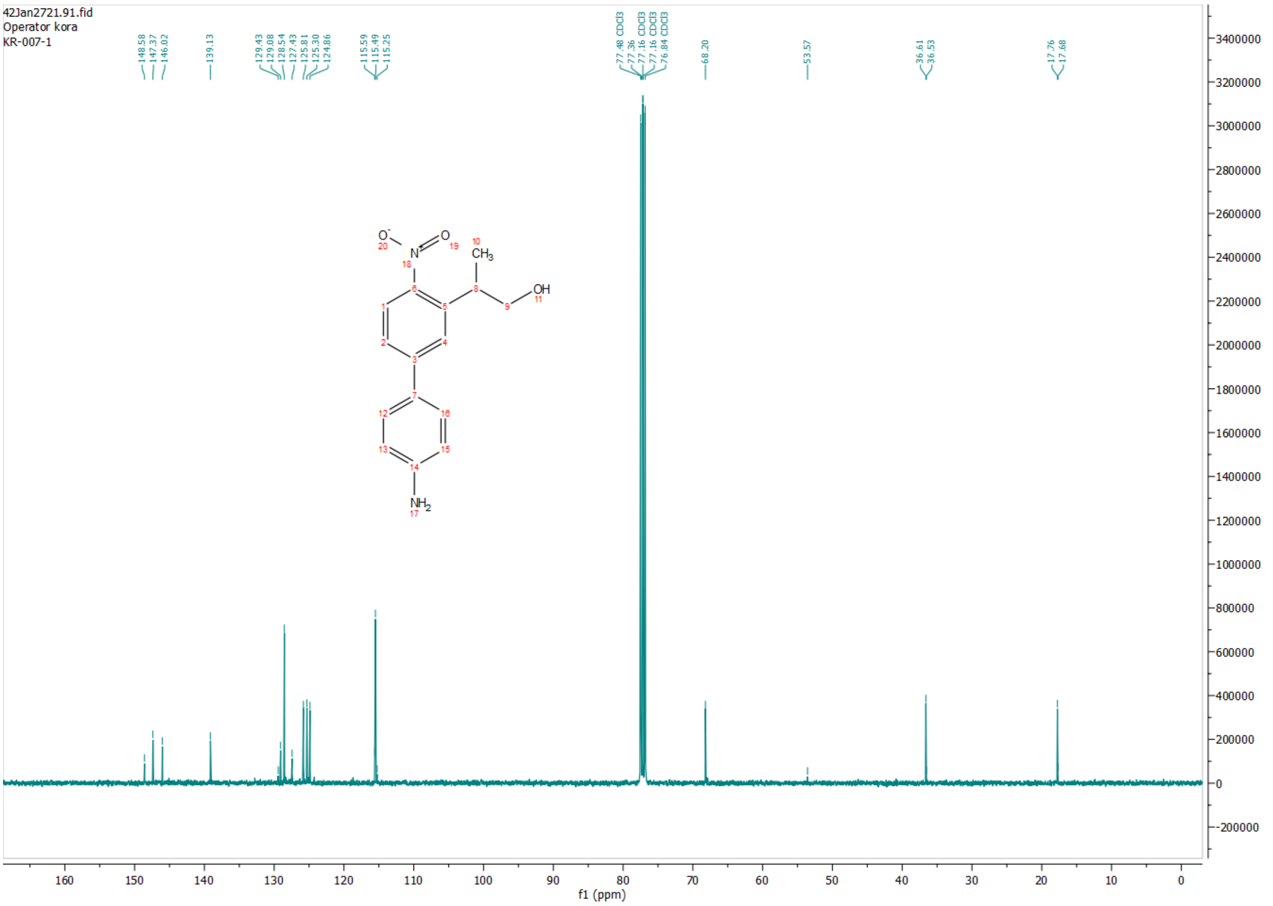

42Jan2721.91.fid
Operator kora
KR-007-1
f1 (ppm)

***t*Bu₂-DCANBP-OH 14**

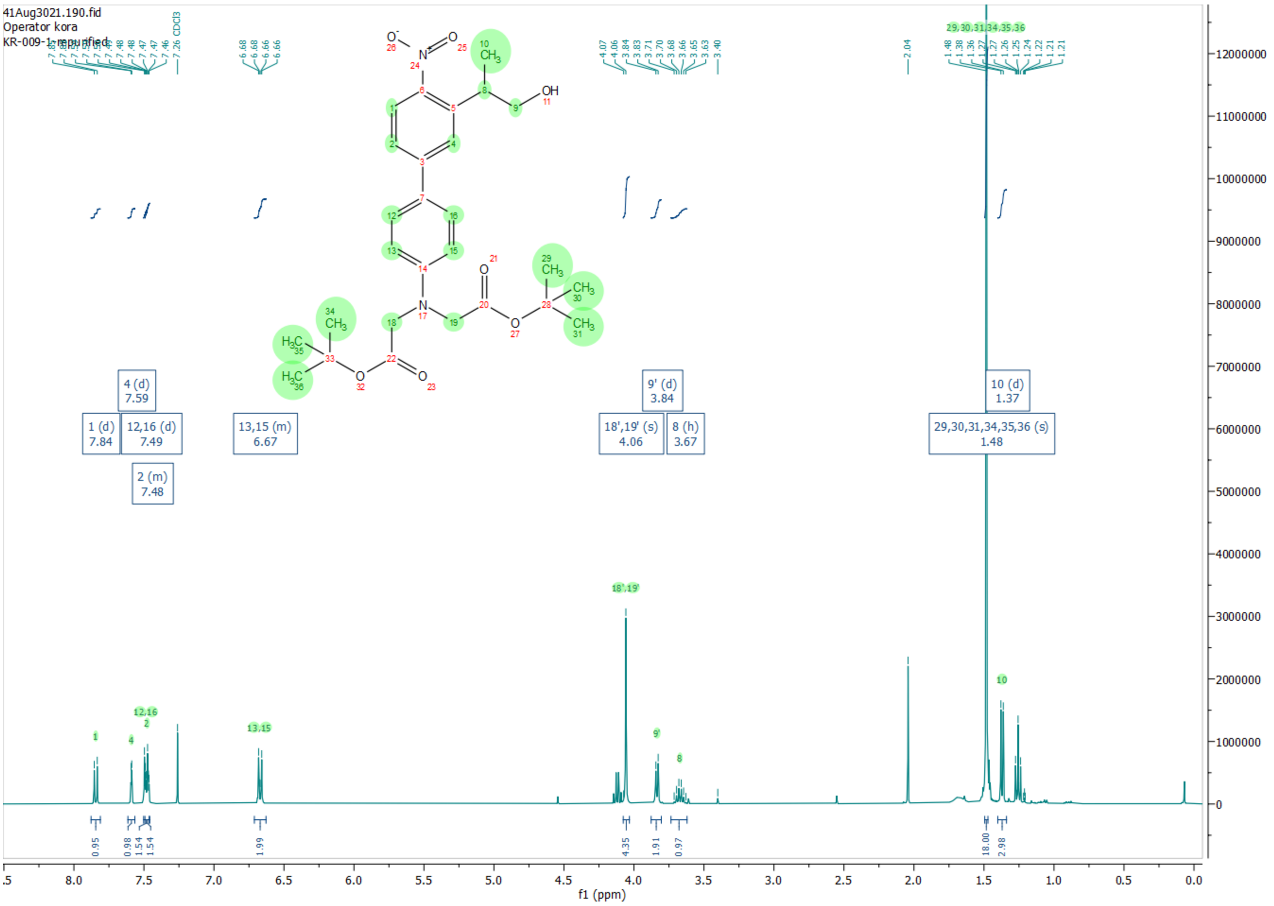

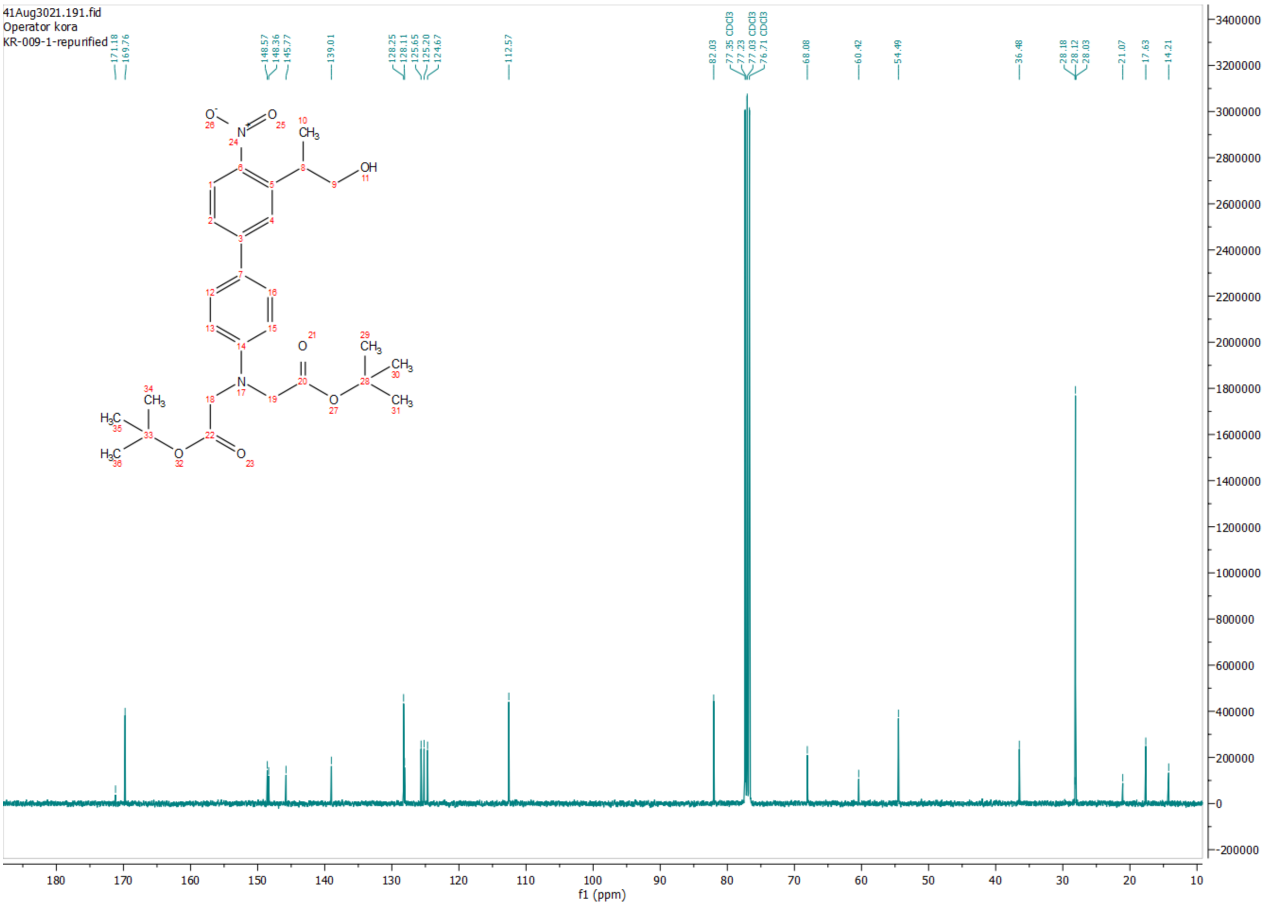

***t*Bu₂-DCANBP-O-Carbonate14a**

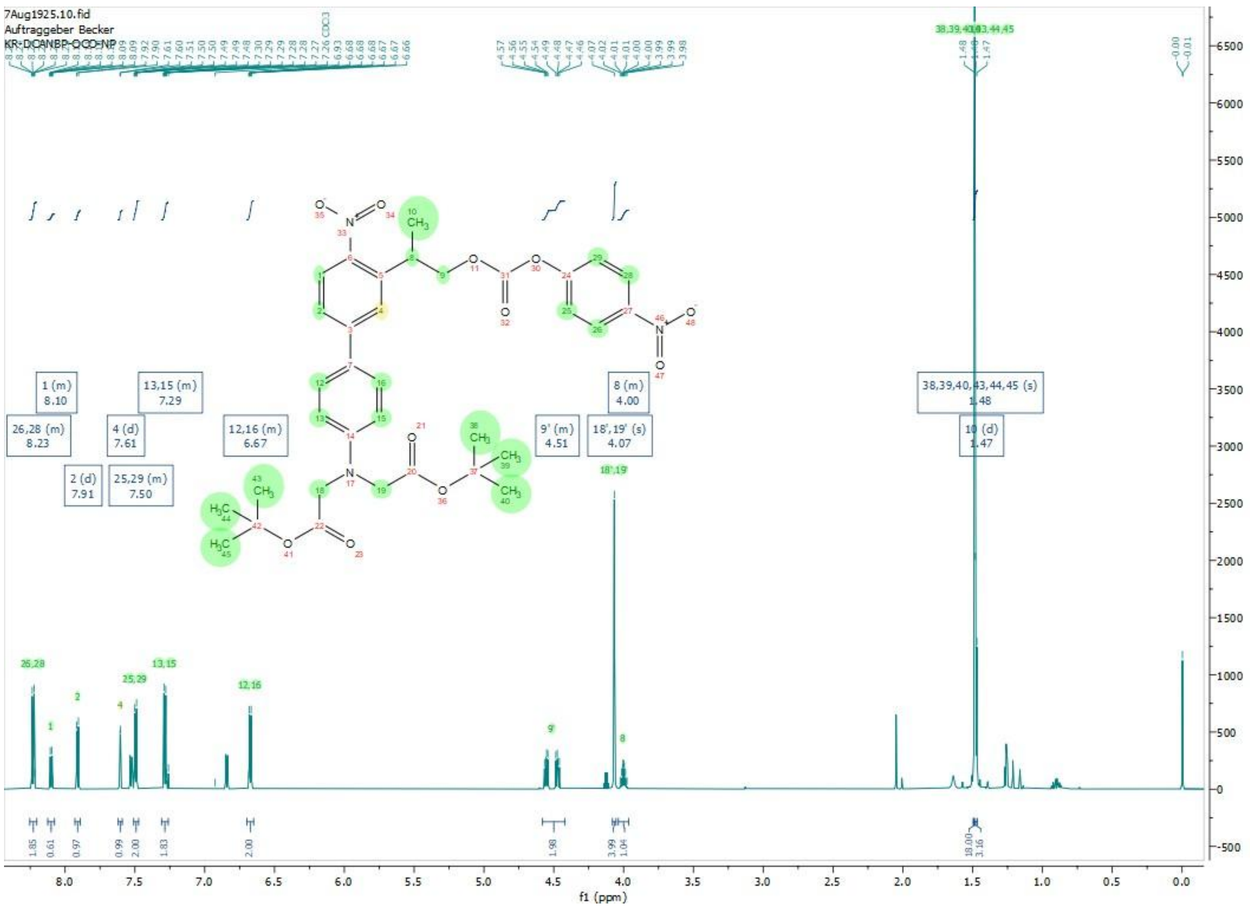

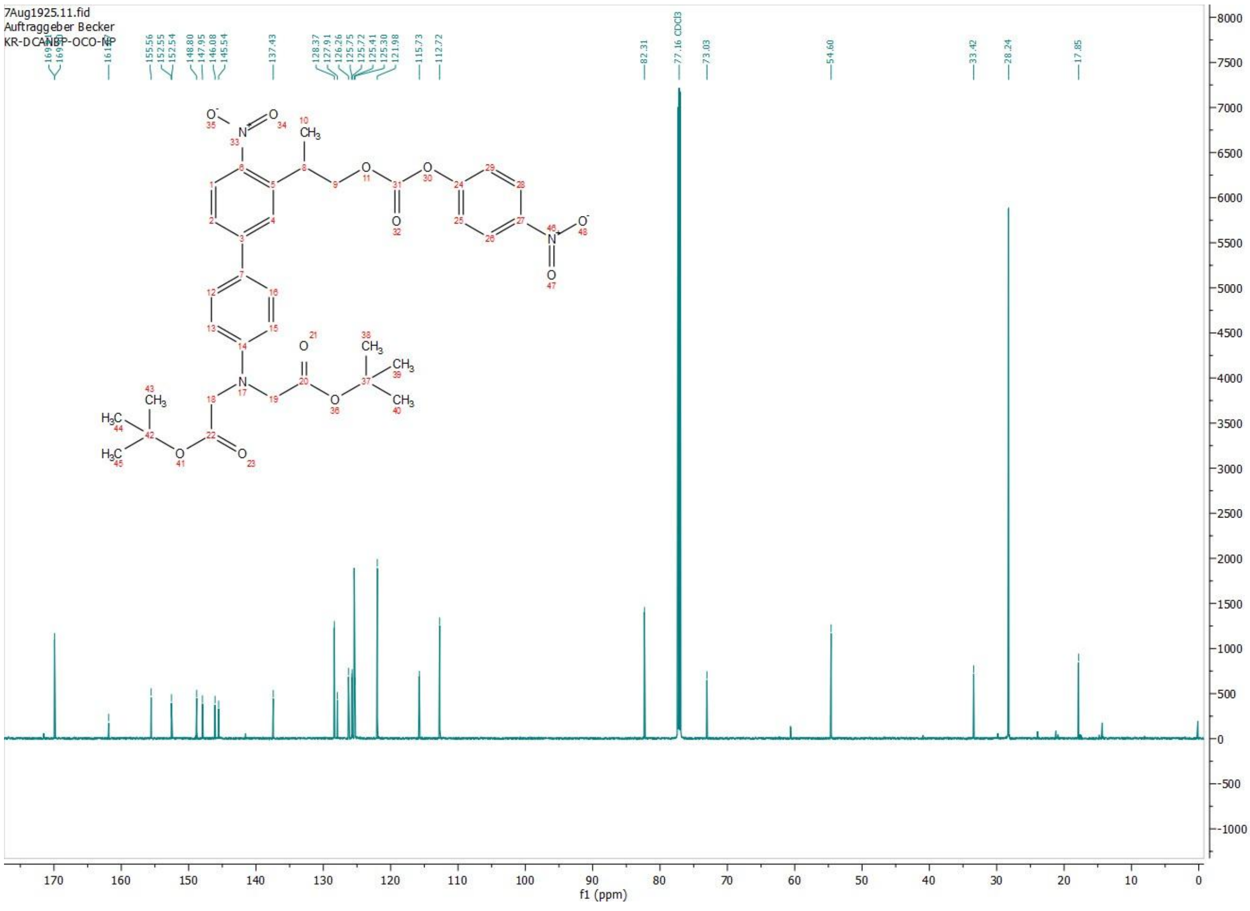

Tetramethyl-BODIPY-OAc 15

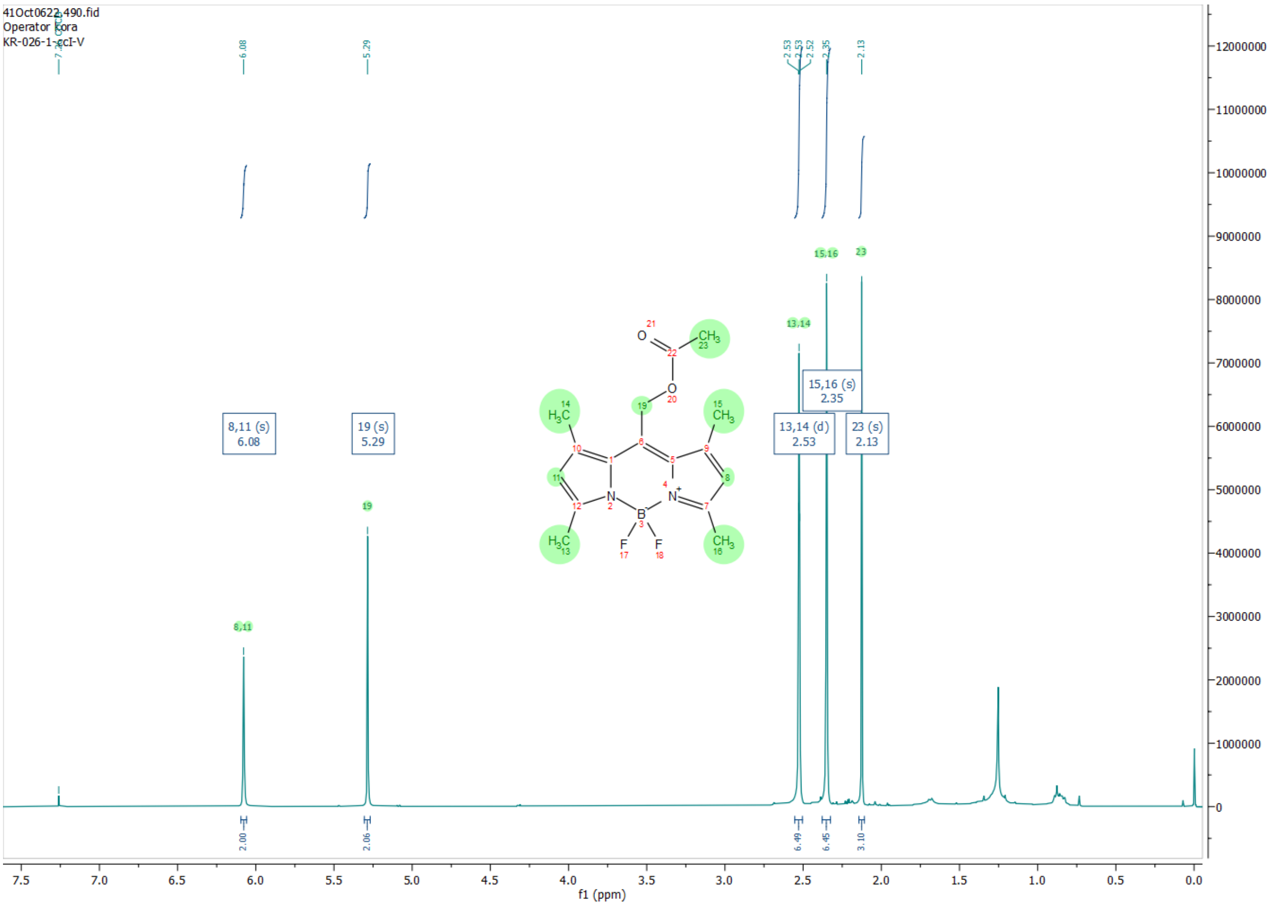

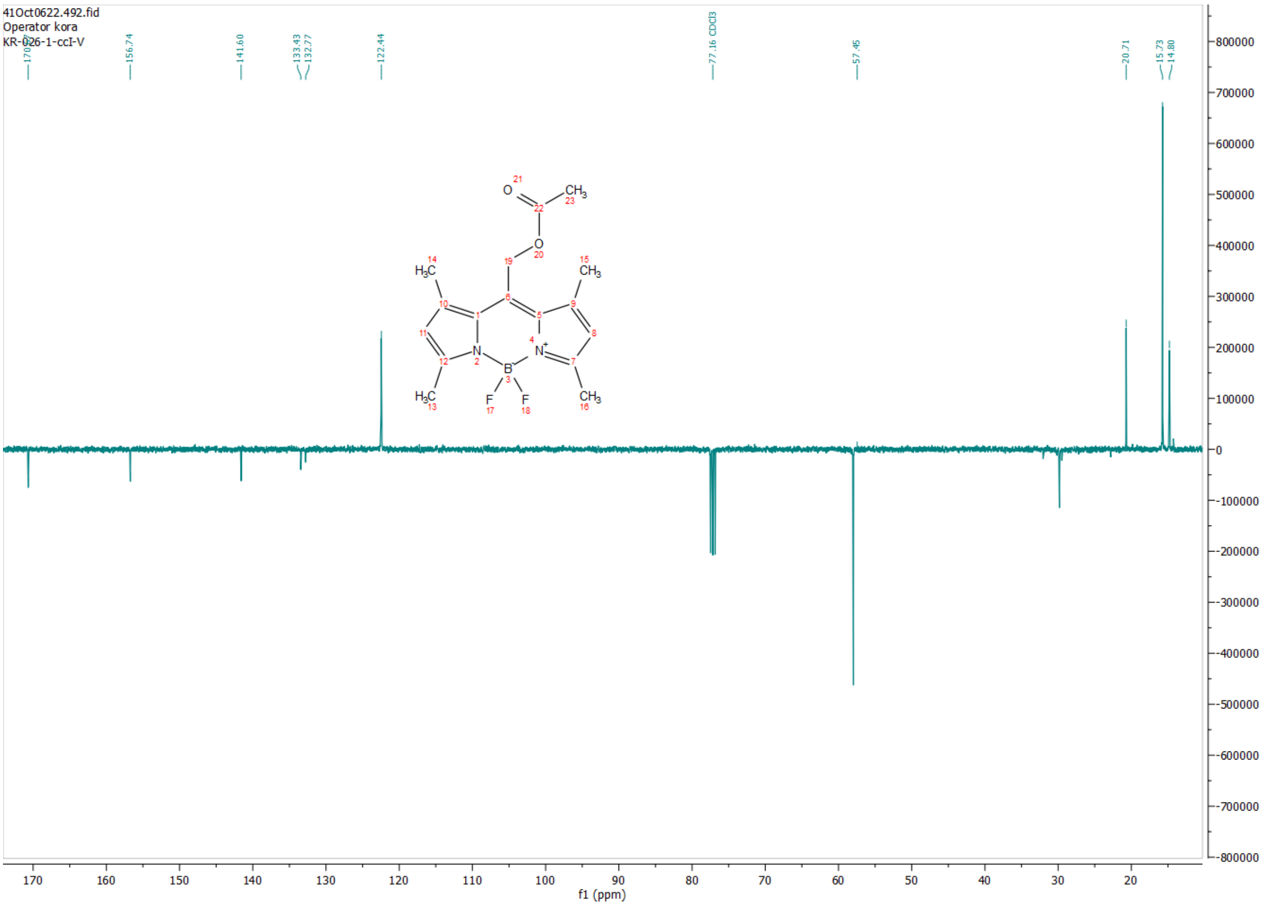

Hexamethyl-BODIPY-OH 16

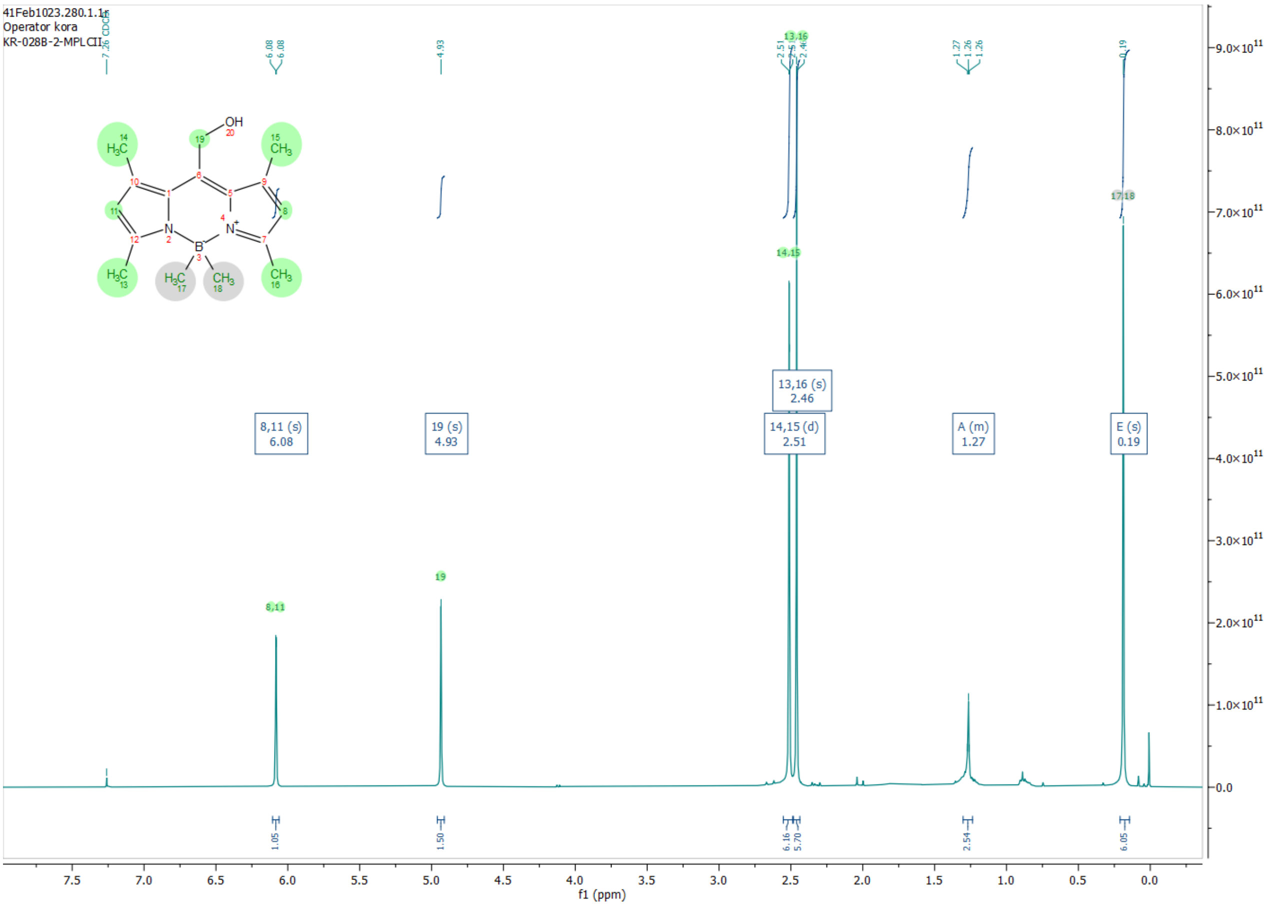

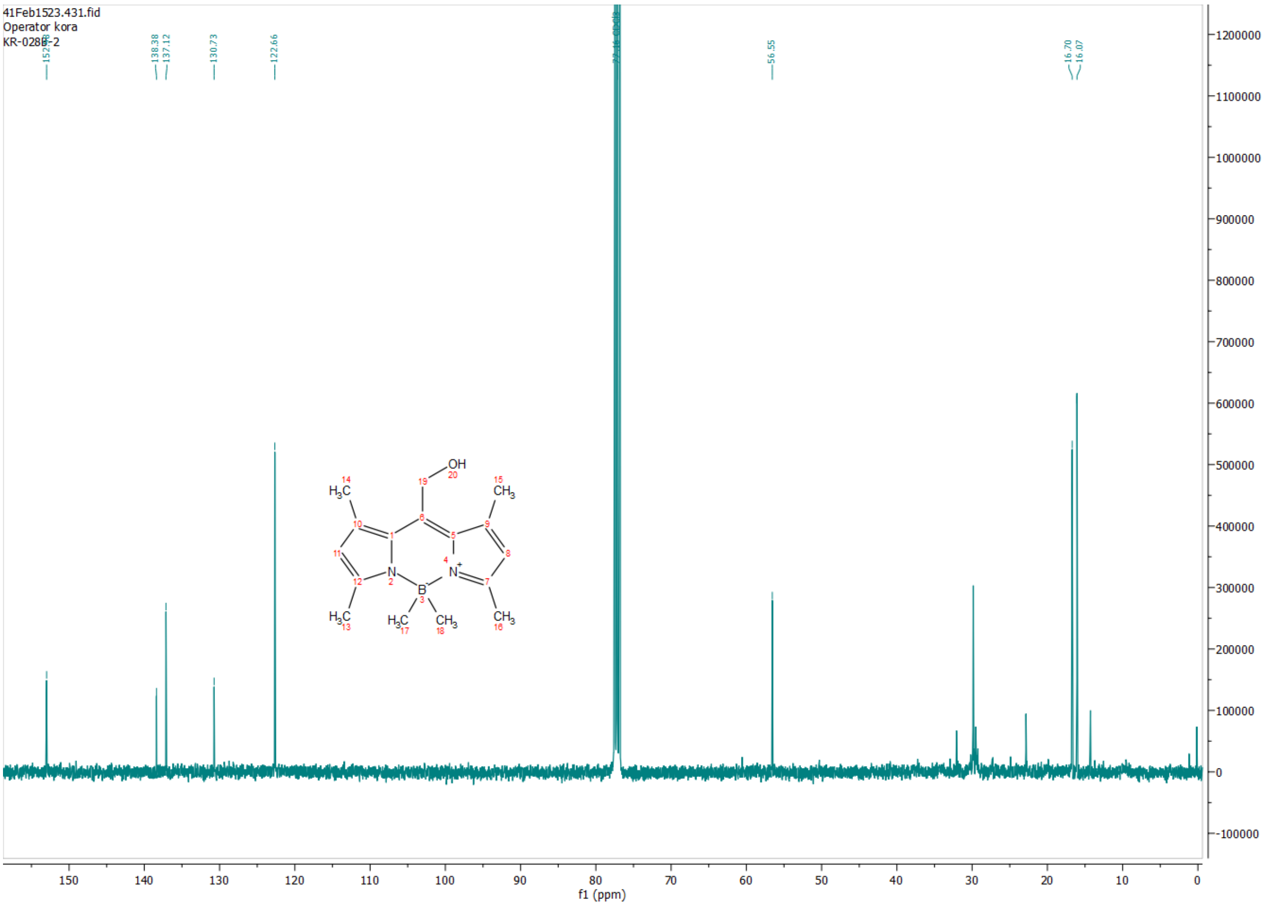

Me_6I_2-BODIPY-OH 17

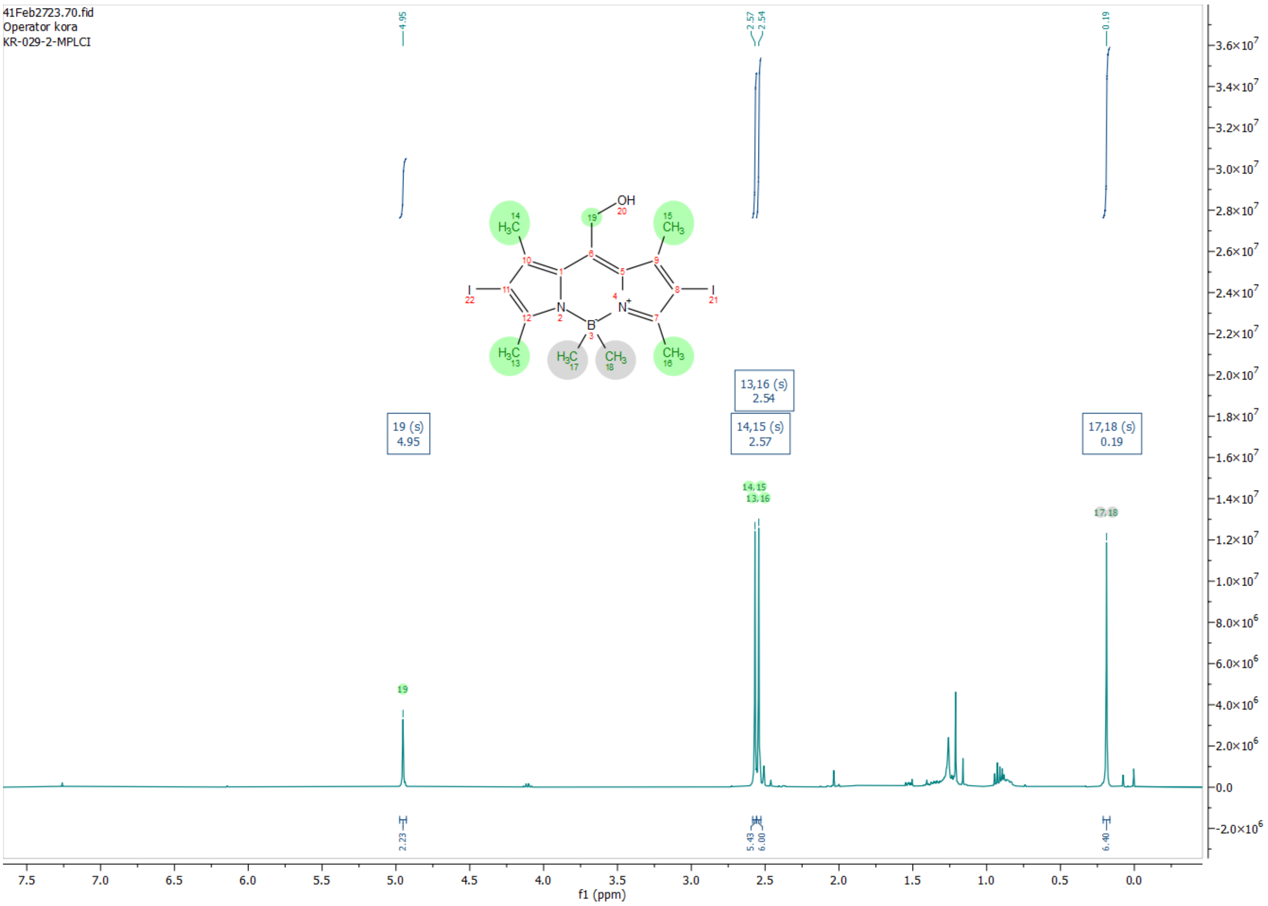

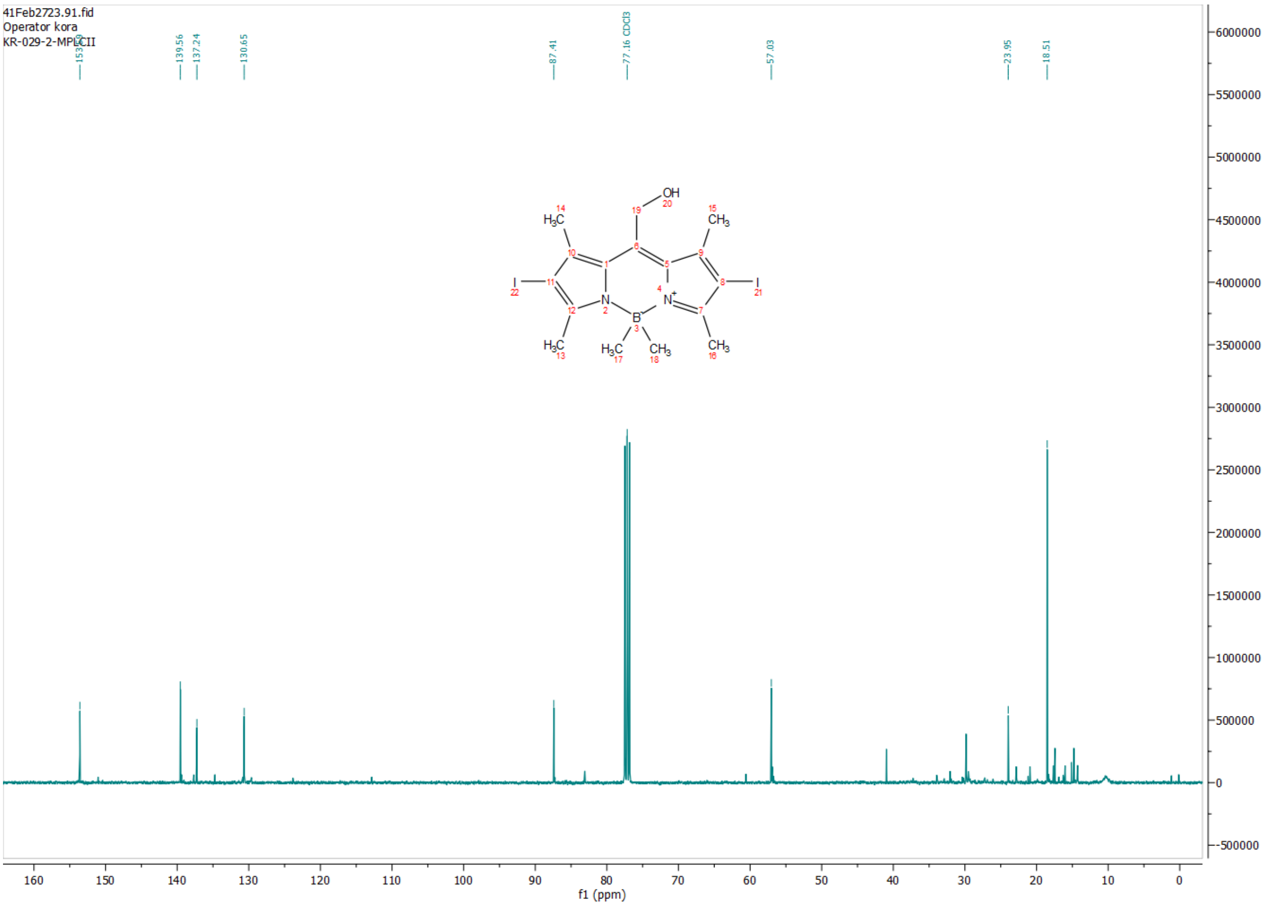

Me$_6$I$_2$-BODIPY-O-Carbonate 17a

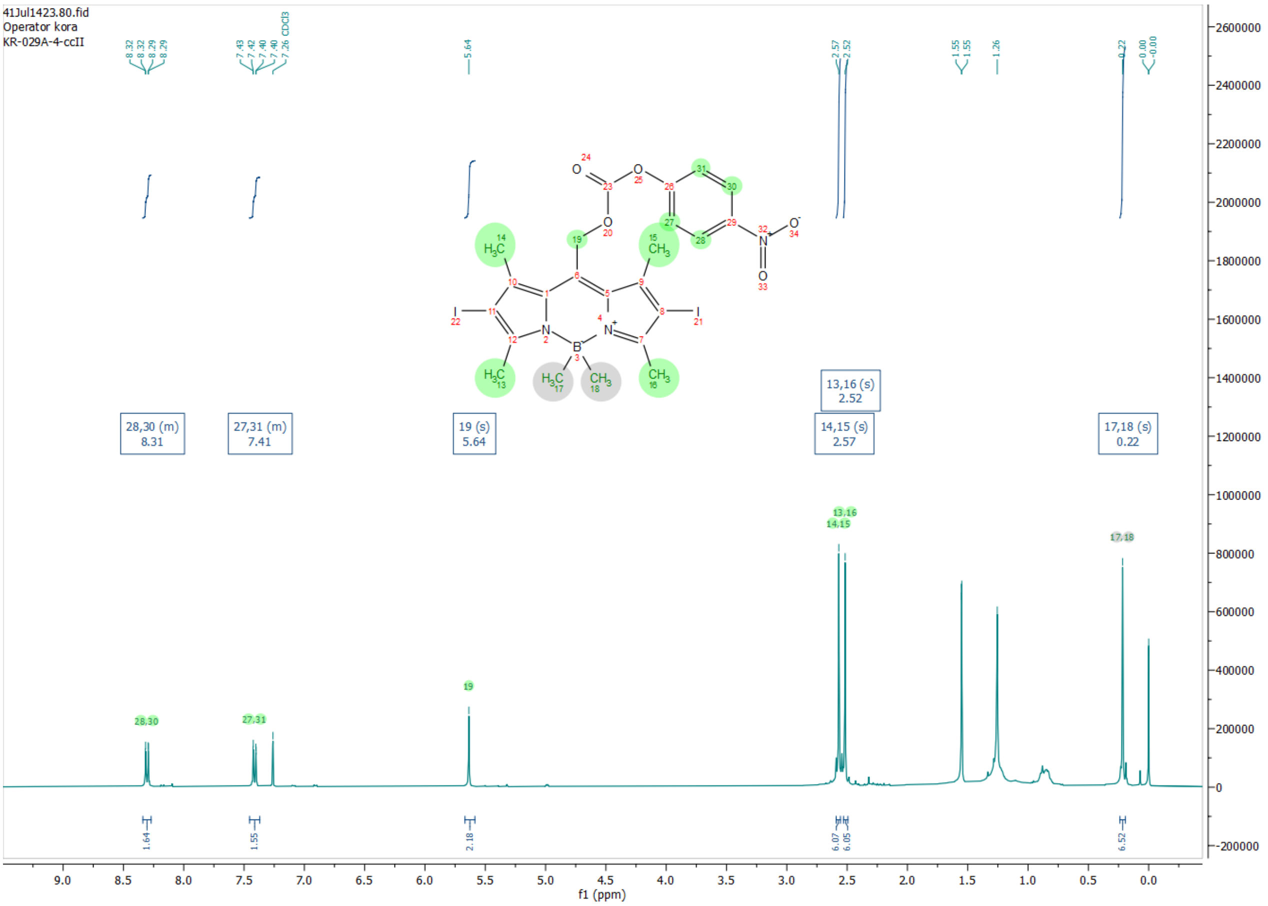

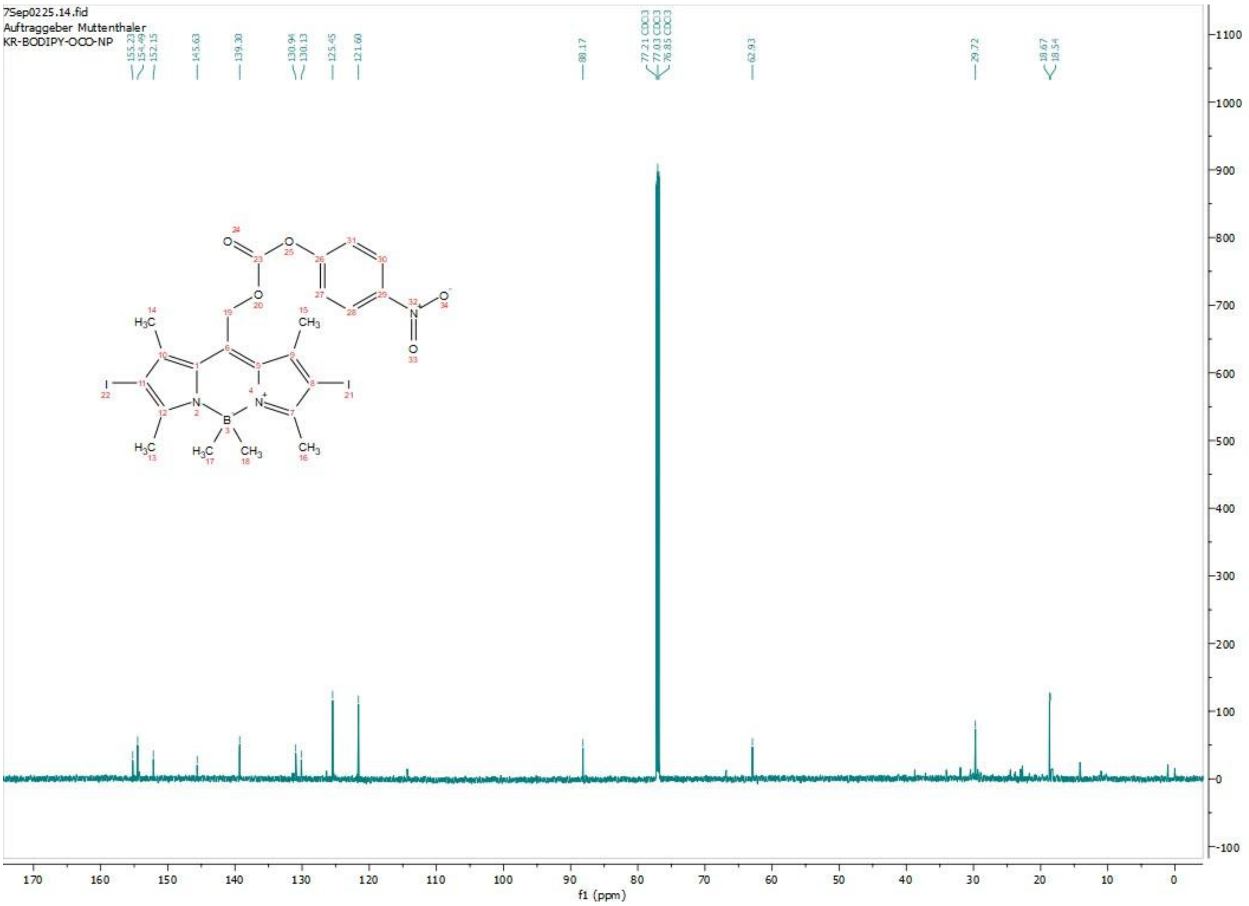

Analytical RP-HPLC 1-6

DCMAC-OT 1

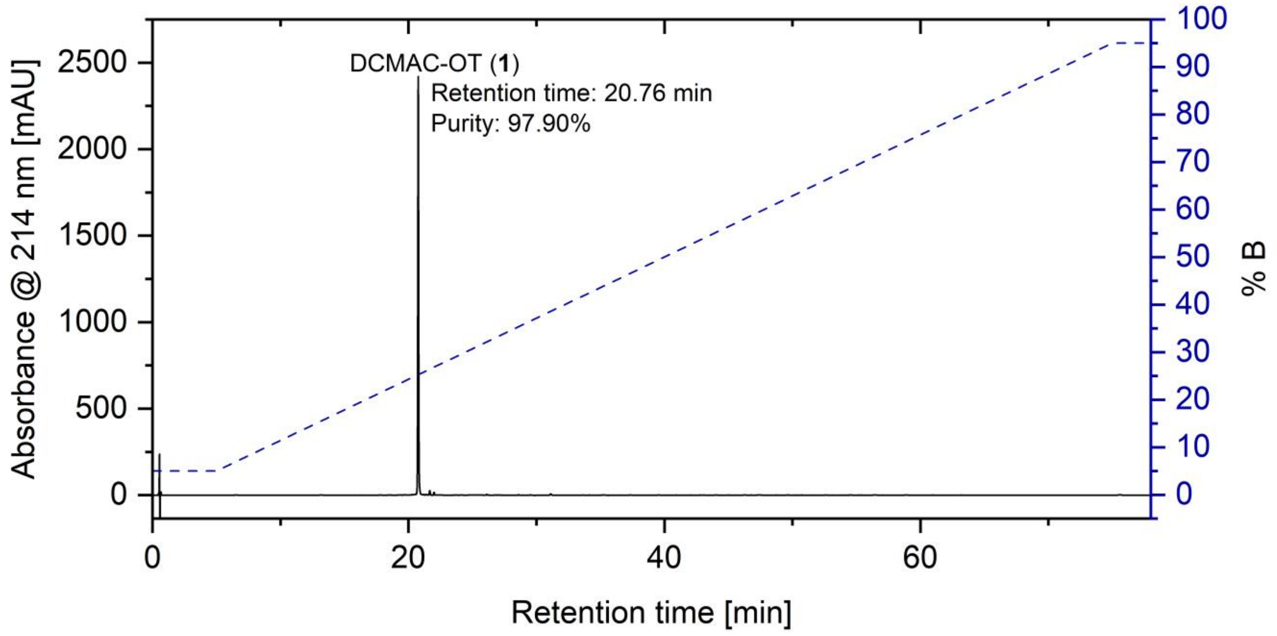

DCMAC-VP 2

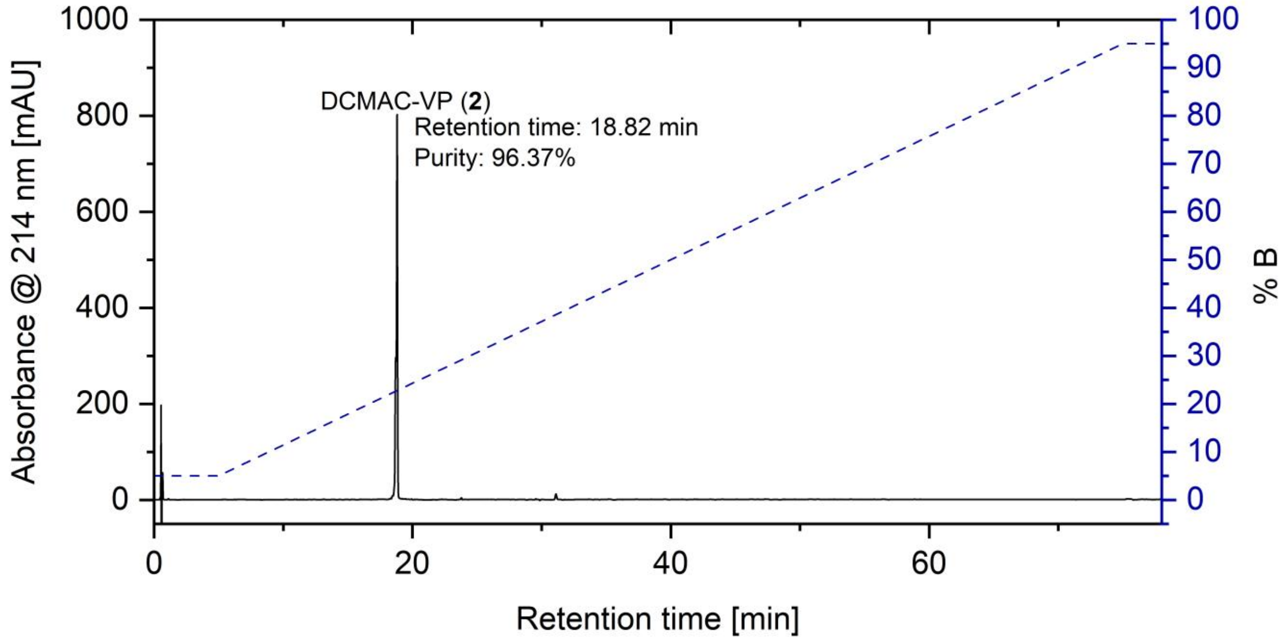

DCANBP-OT 3

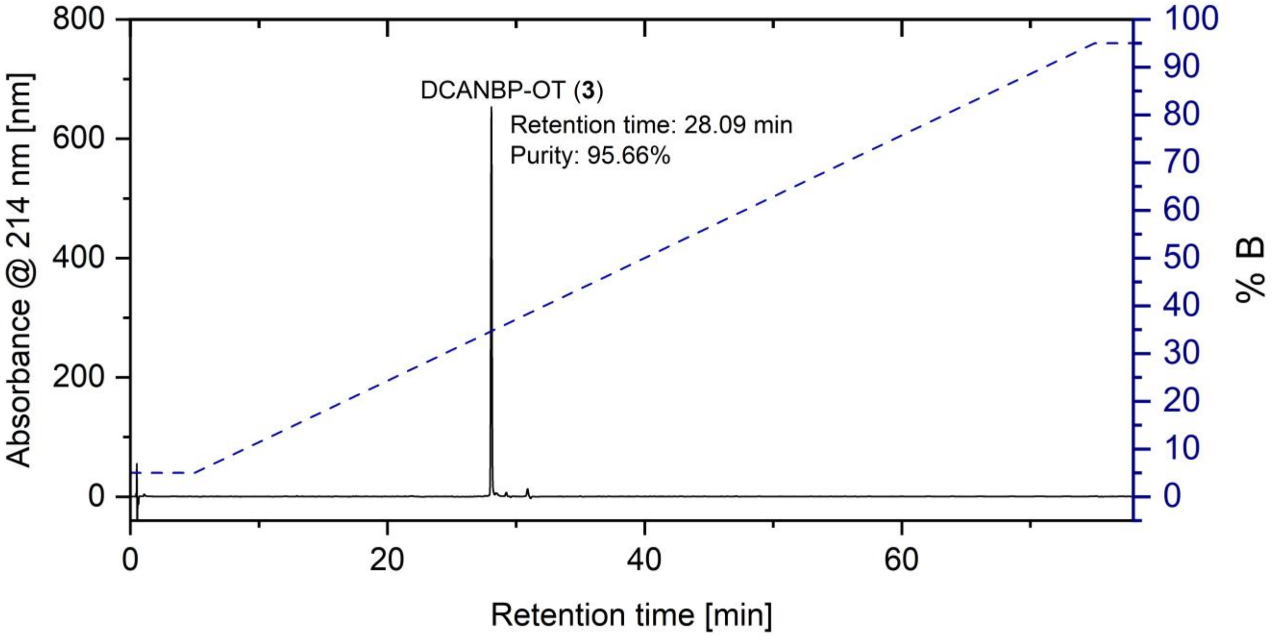

DCNABP-VP 4

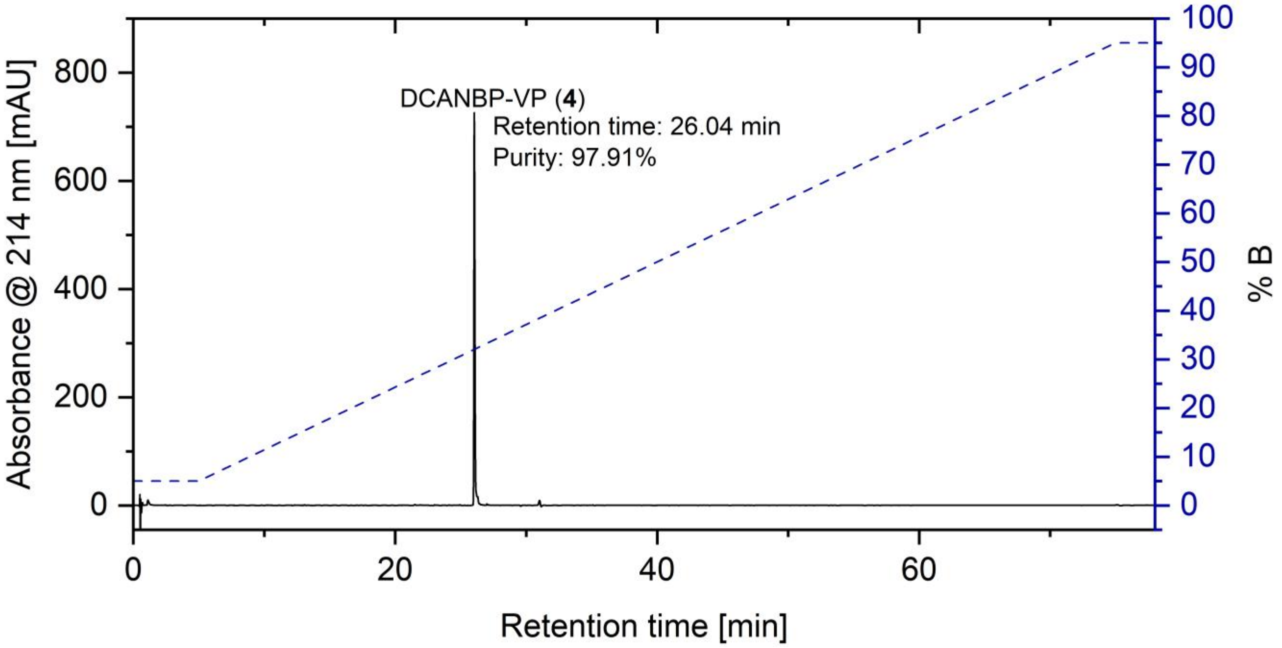

Me$_6$I$_2$-BODIPY-OT 5

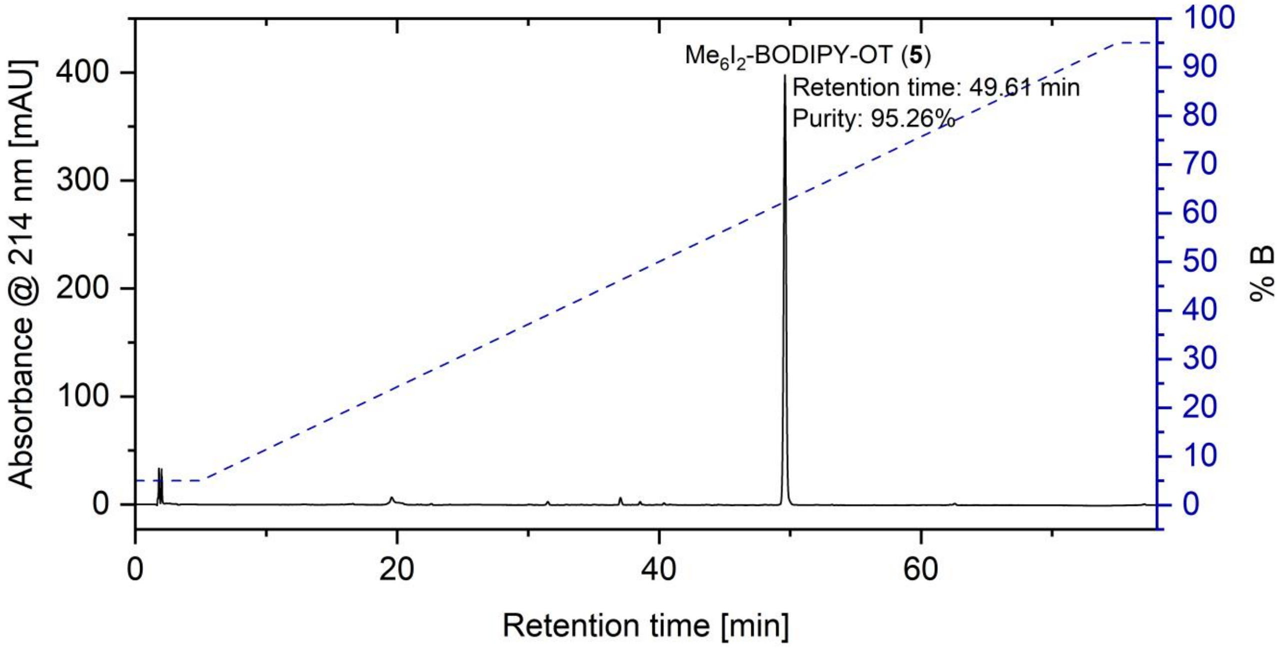

Me$_6$I$_2$-BODIPY-VP 6

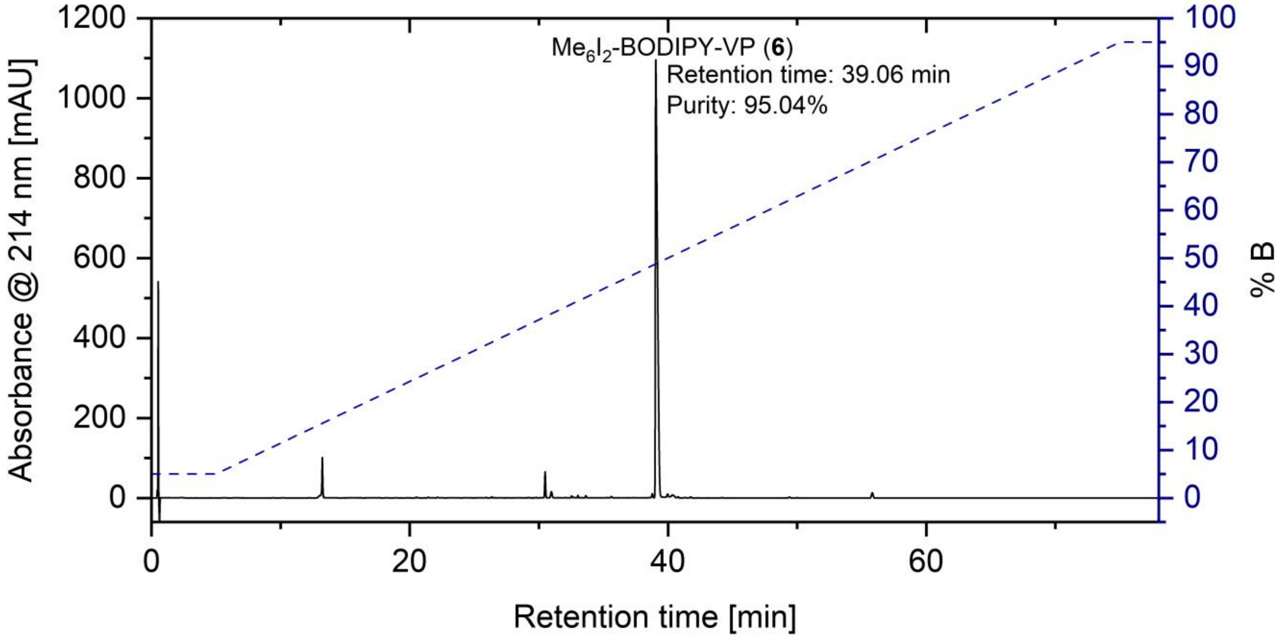

High-resolution mass spectra of compounds 1-17

DCMAC-OT 1

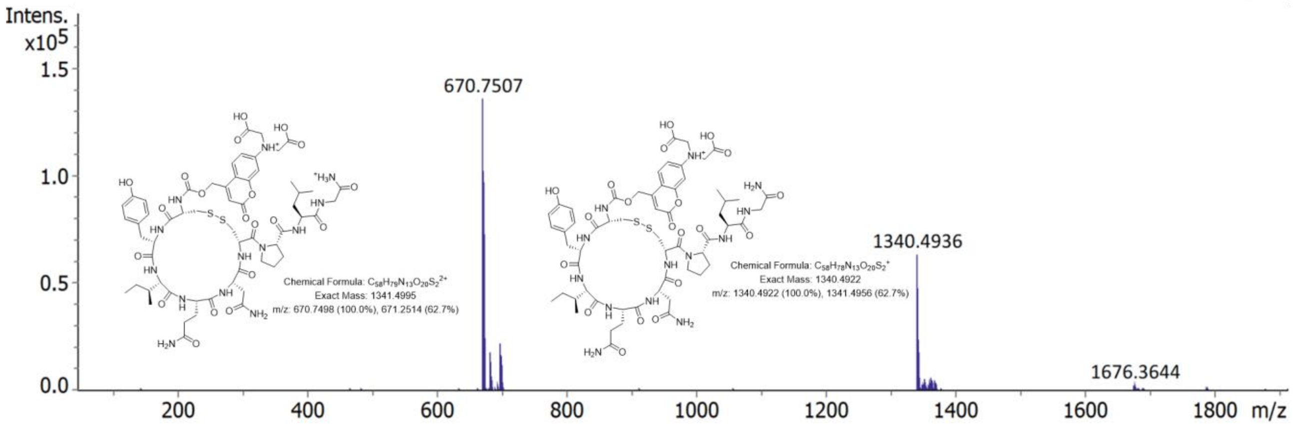

DCMAC-VP 2

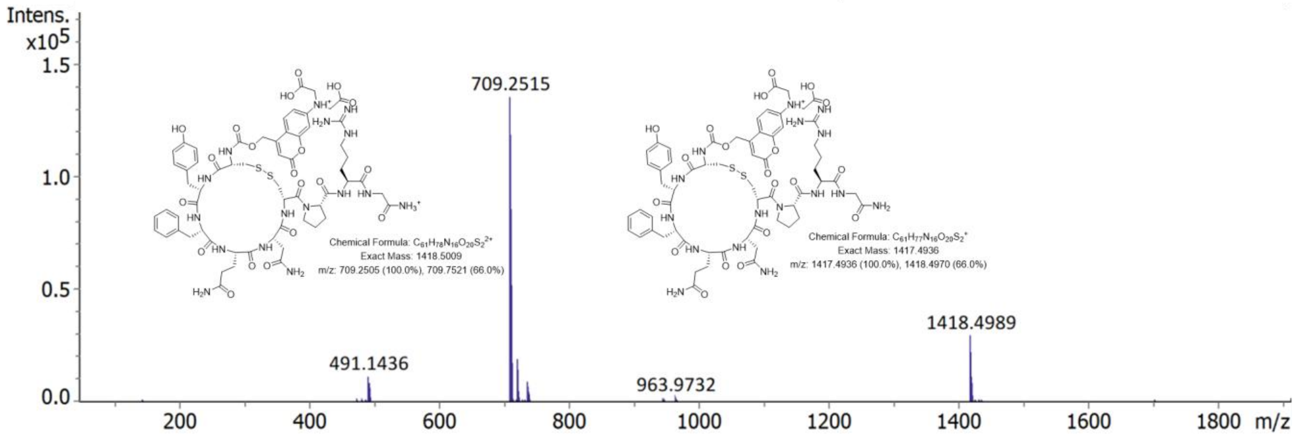

DCANBP-OT 3

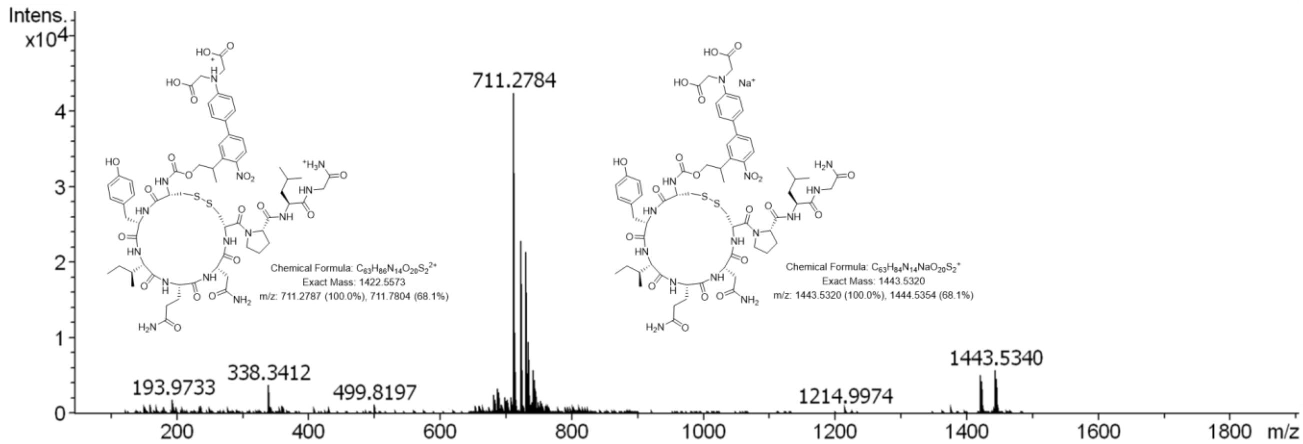

DCANBP-VP 4

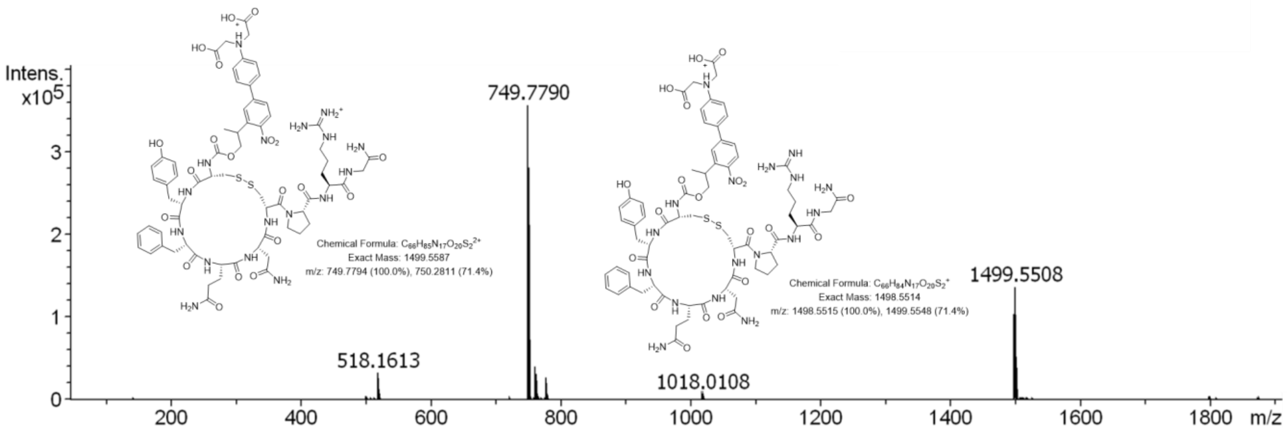

Me₆I₂-BODIPY-OT 5

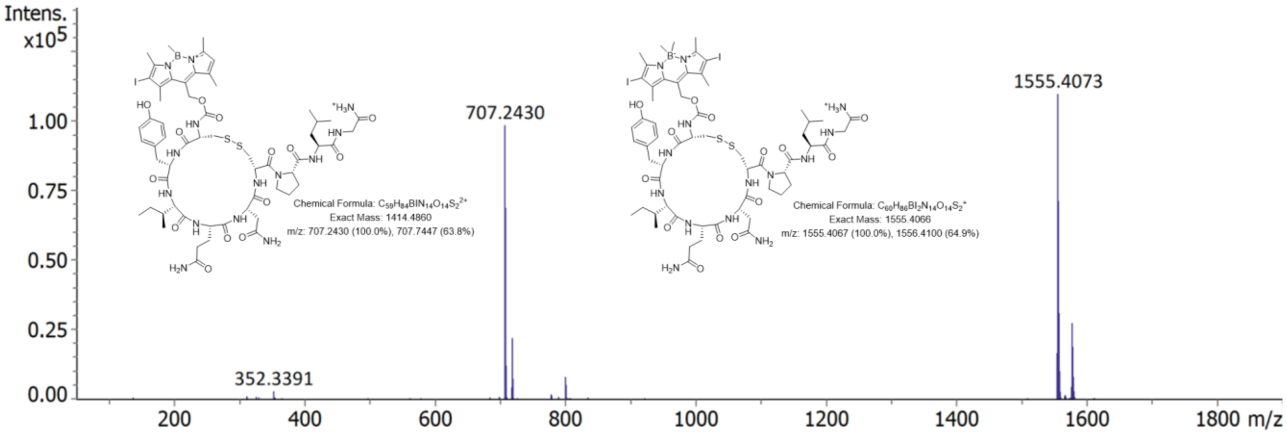

Me₆I₂-BODIPY-VP 6

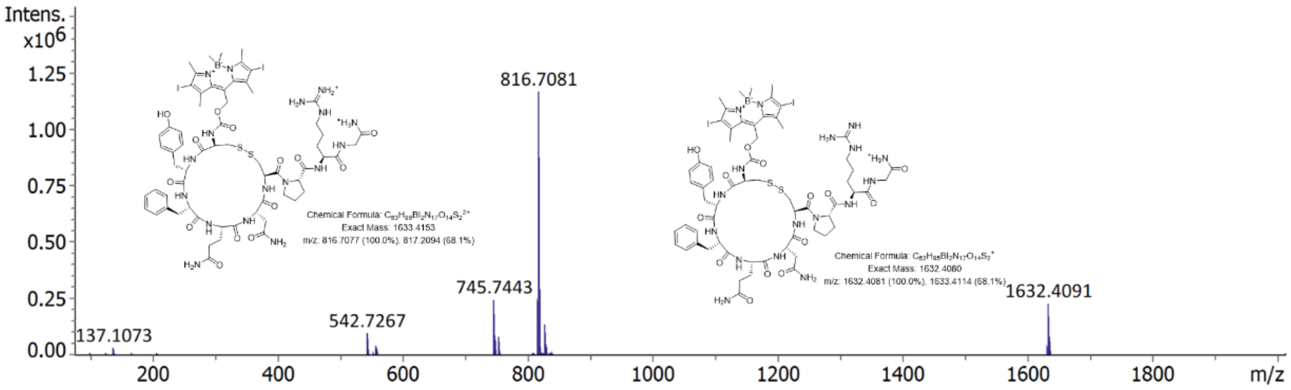

***t*Bu$_2$-DCMAC 7**

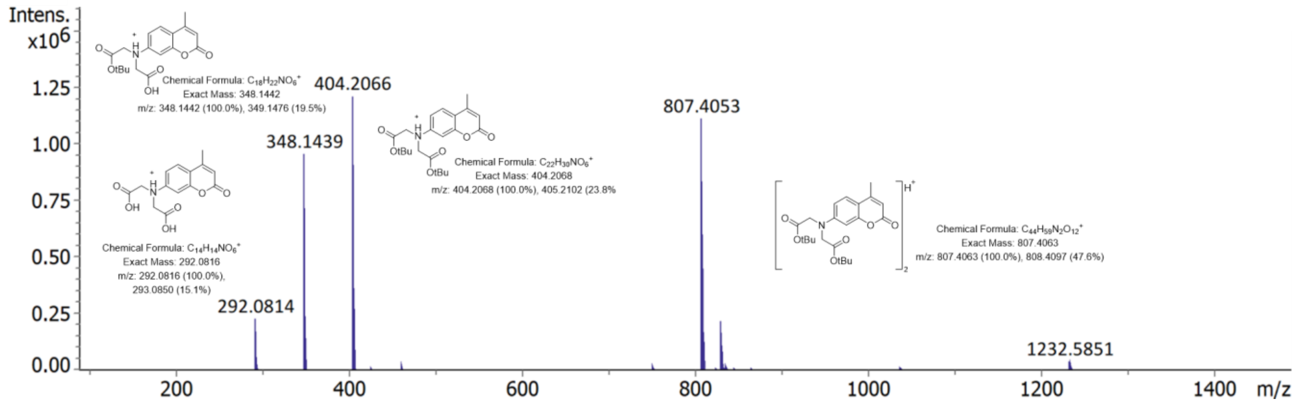

***t*Bu$_2$-DCMAC-CHO 8**

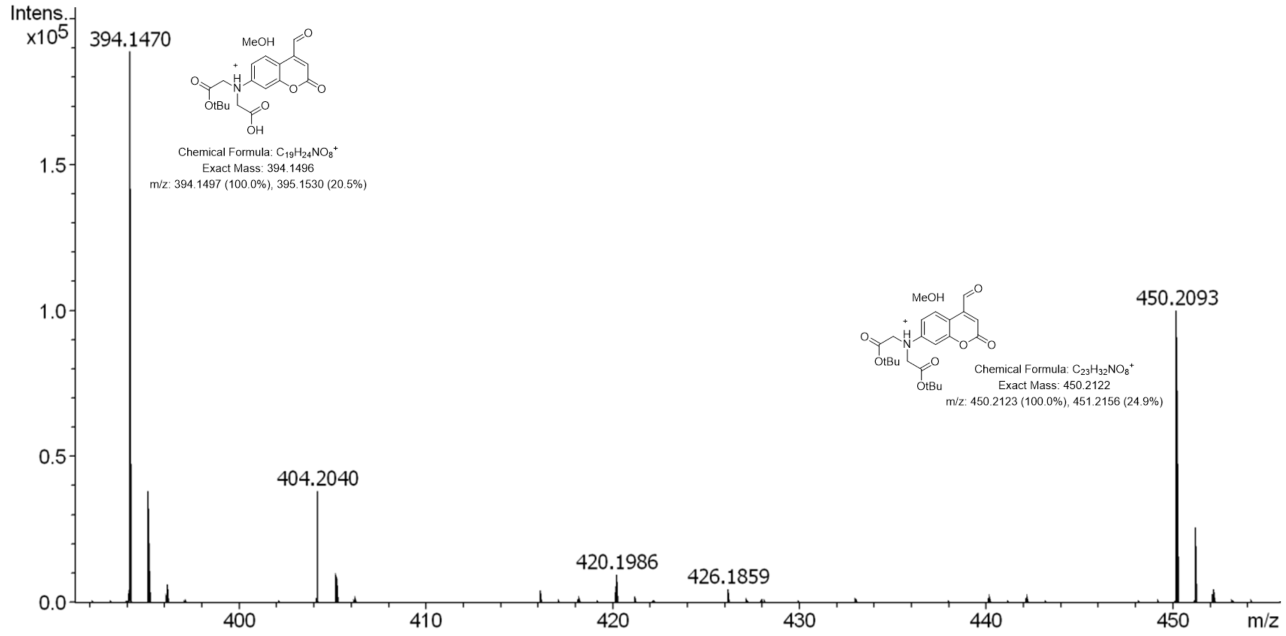

***t*Bu$_2$-DCMAC-OH 9**

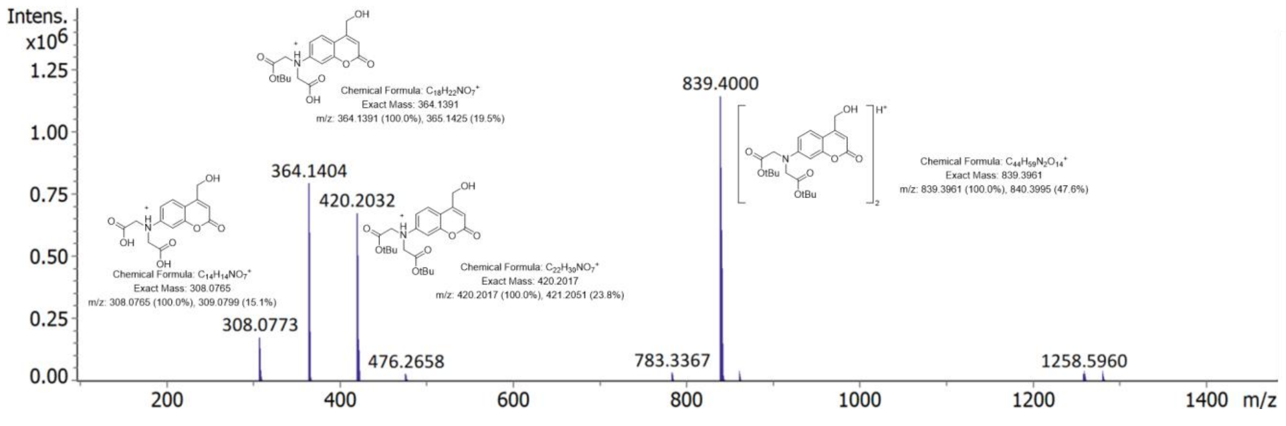

***t*Bu₂-DCMAC-O-Carbonate 9a**

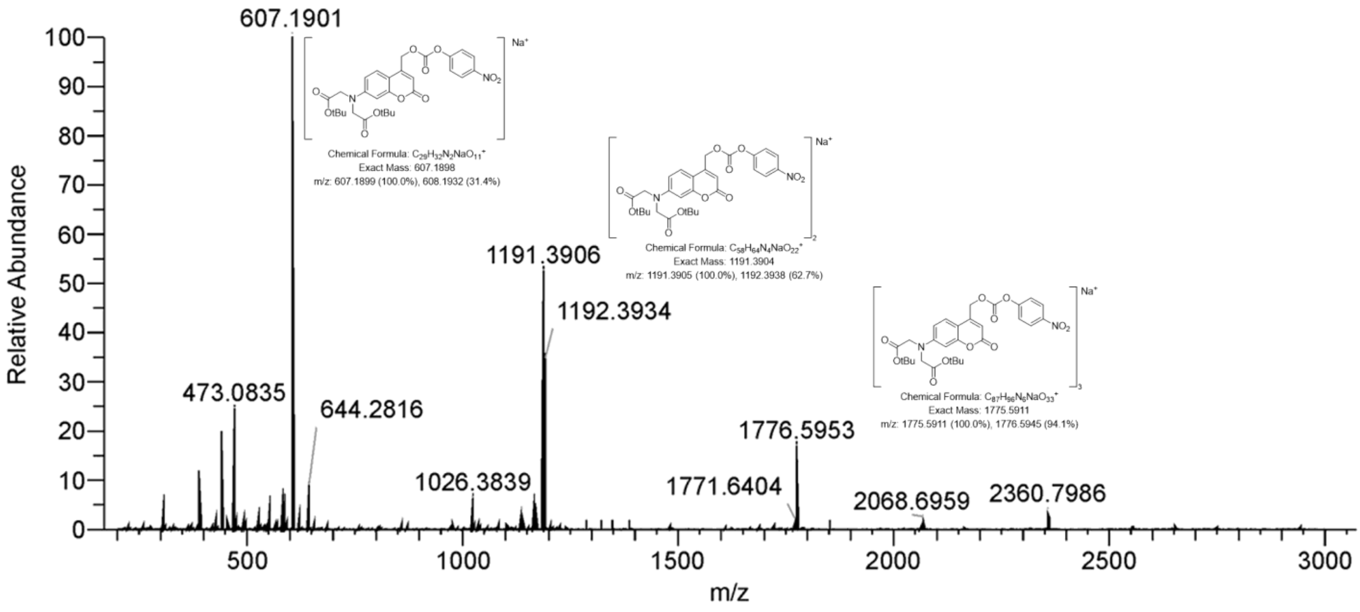

***tert*-Butyl 2-(5-bromo-2-nitrophenyl) acetate 10**

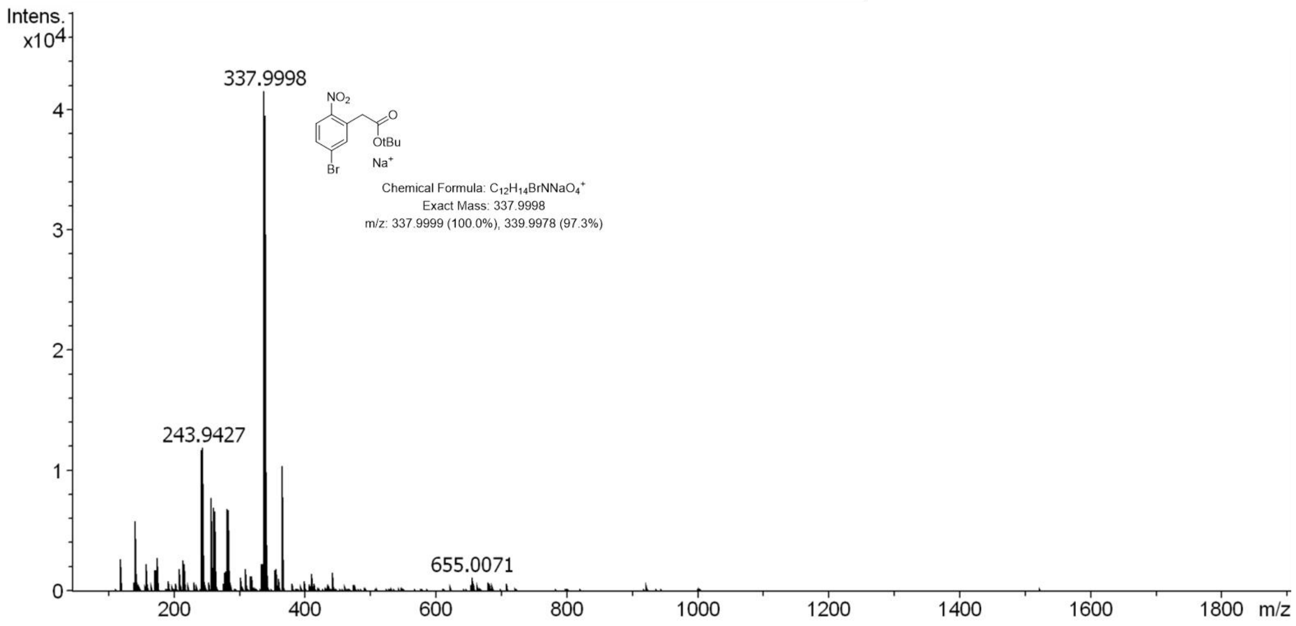

tert-Butyl 2-(5-bromo-2-nitrophenyl)propanoate 11

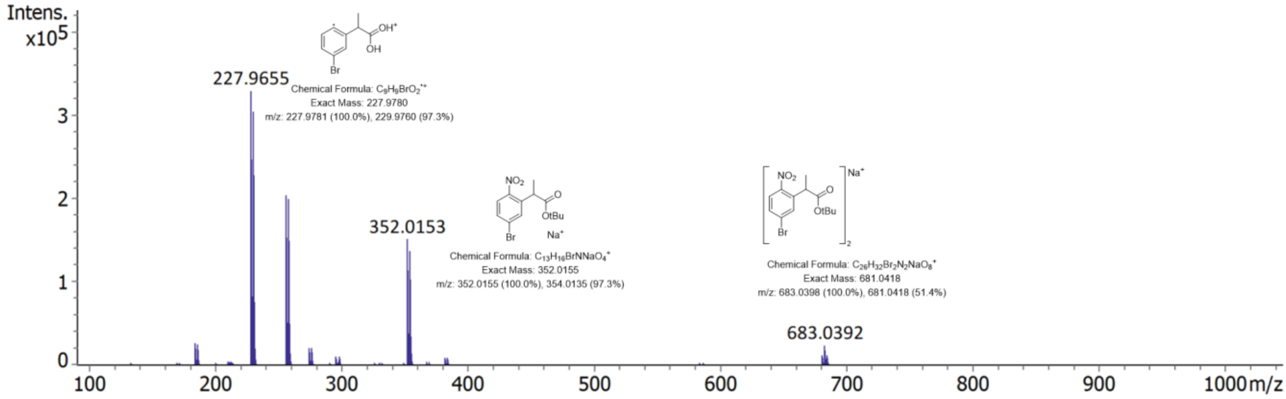

2-(5-Bromo-2-nitrophenyl)propan-1-ol 12

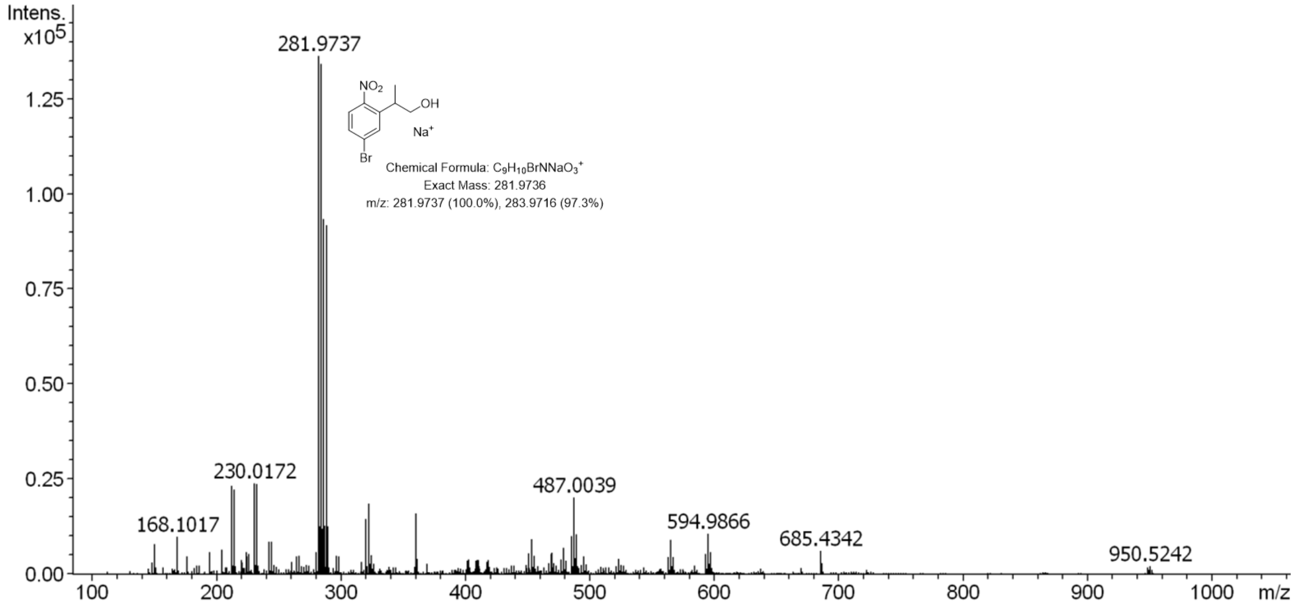

ANBP-OH 13

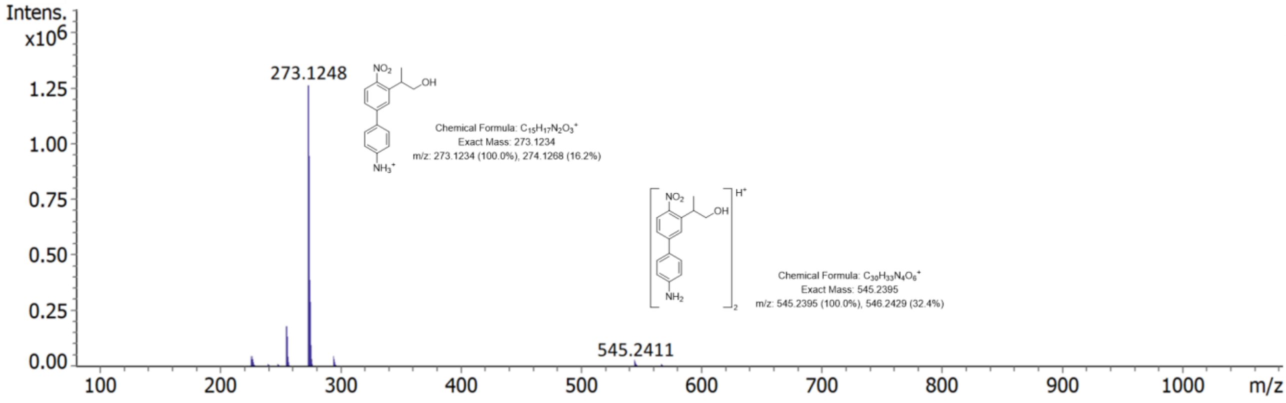

tBu_2-DCANBP-OH 14

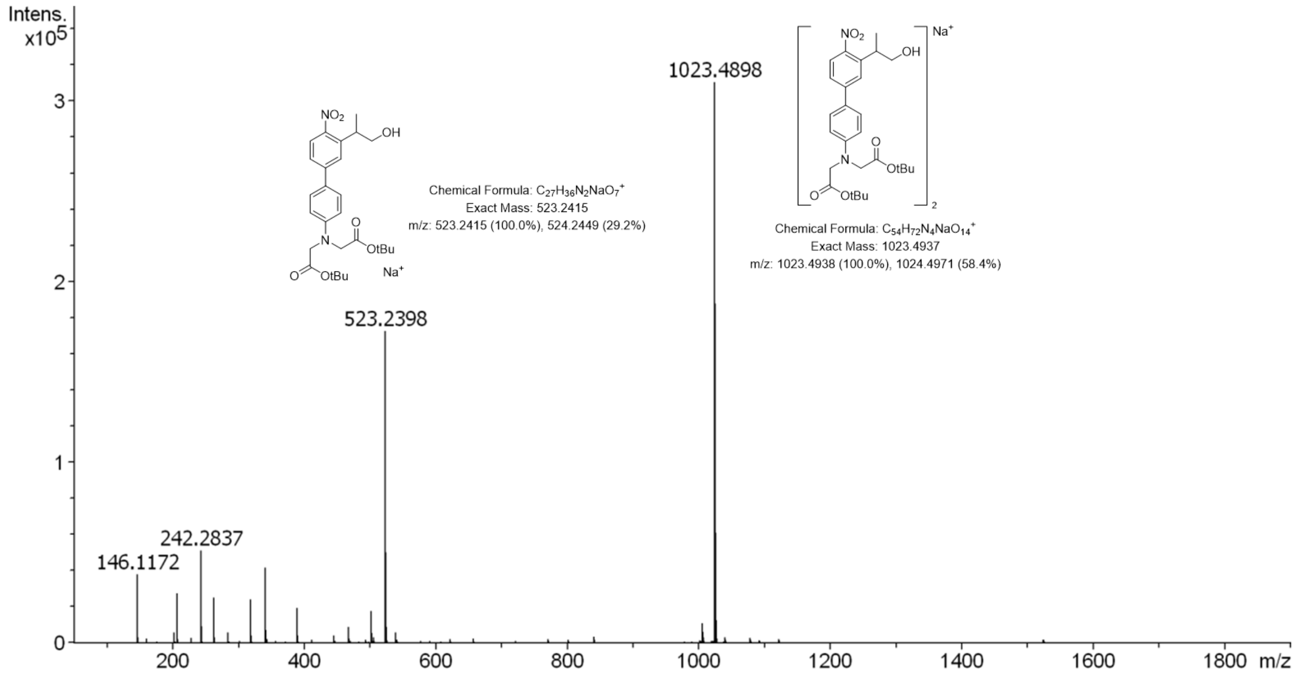

tBu_2-DCANBP-O-Carbonate 14a

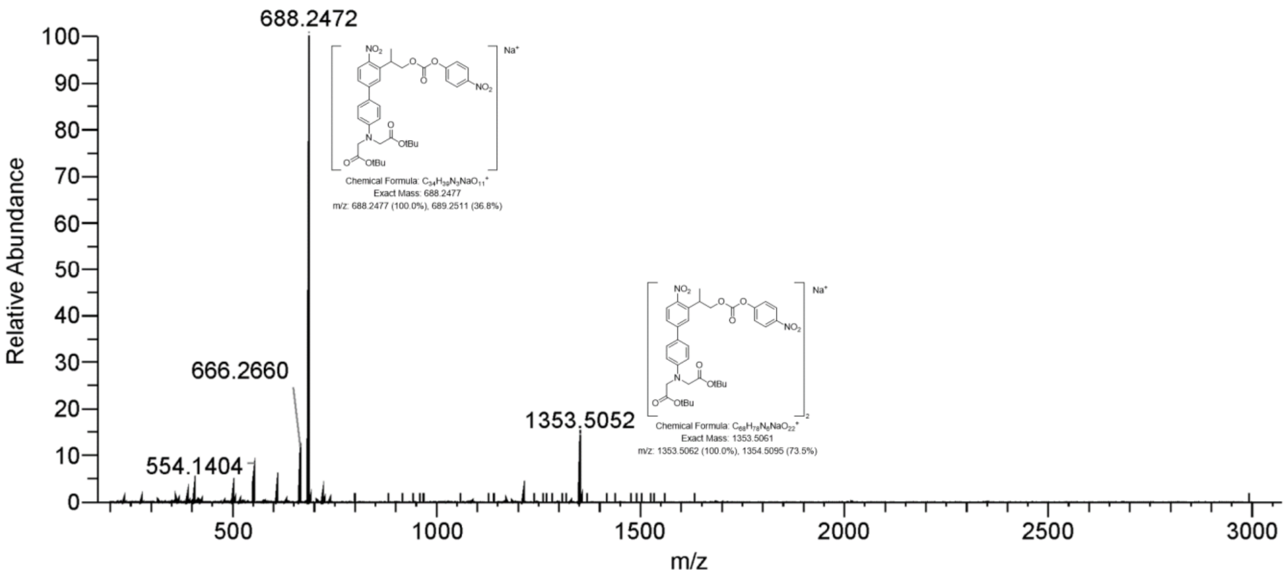

Tetramethyl-BODIPY-OAc 15

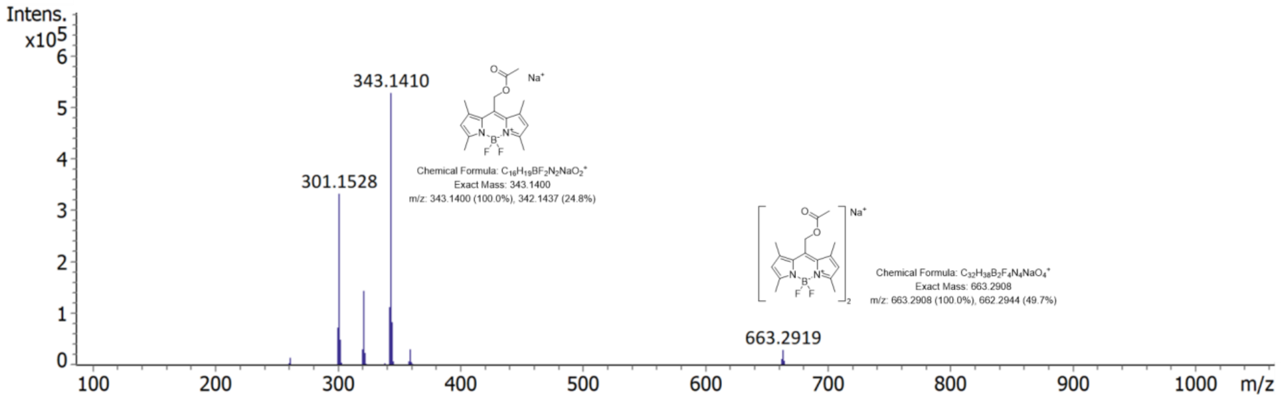

Hexamethyl-BODIPY-OH 16

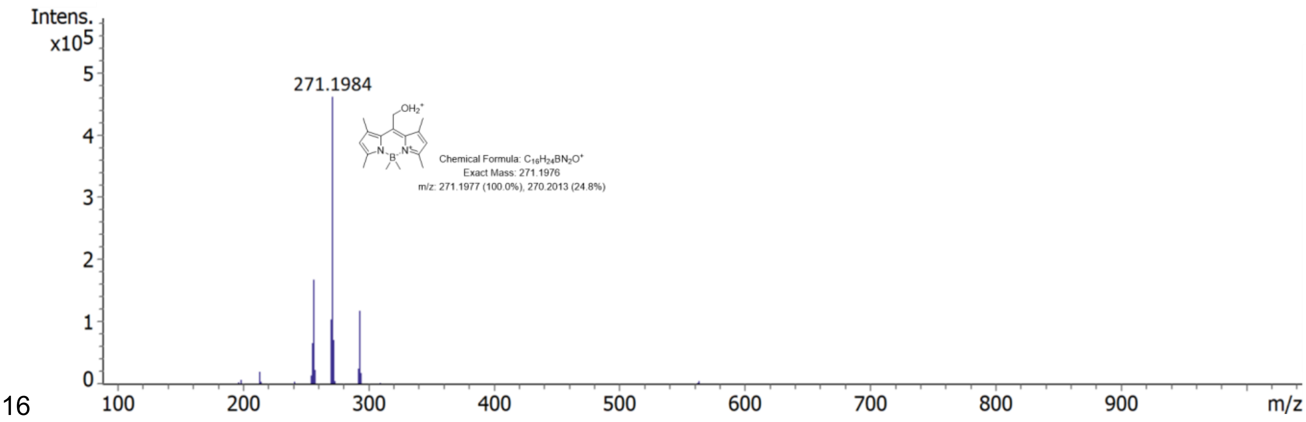

16

Me₆I₂-BODIPY-OH 17

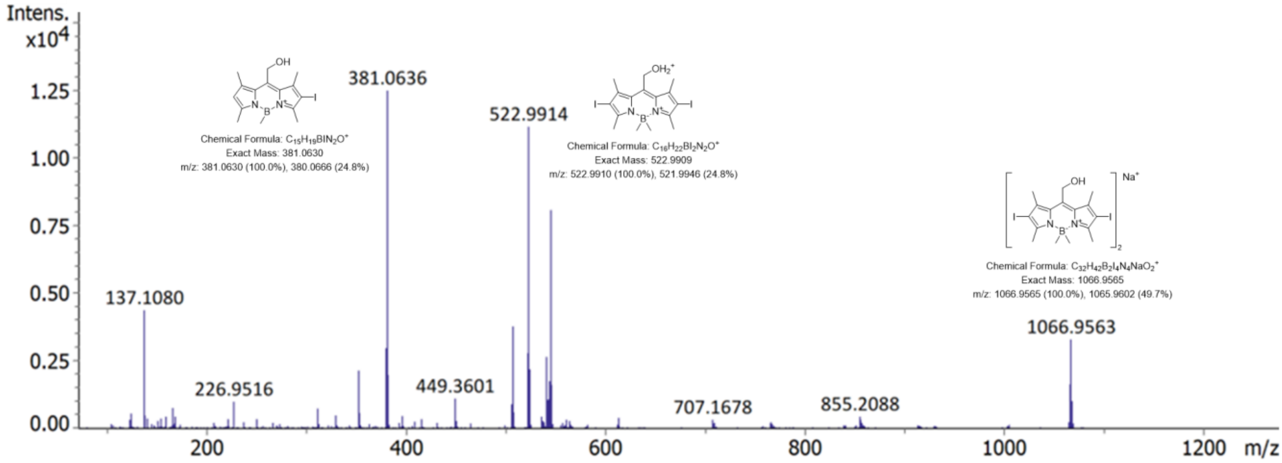

Me_6I_2-BODIPY-O-Carbonate 17a

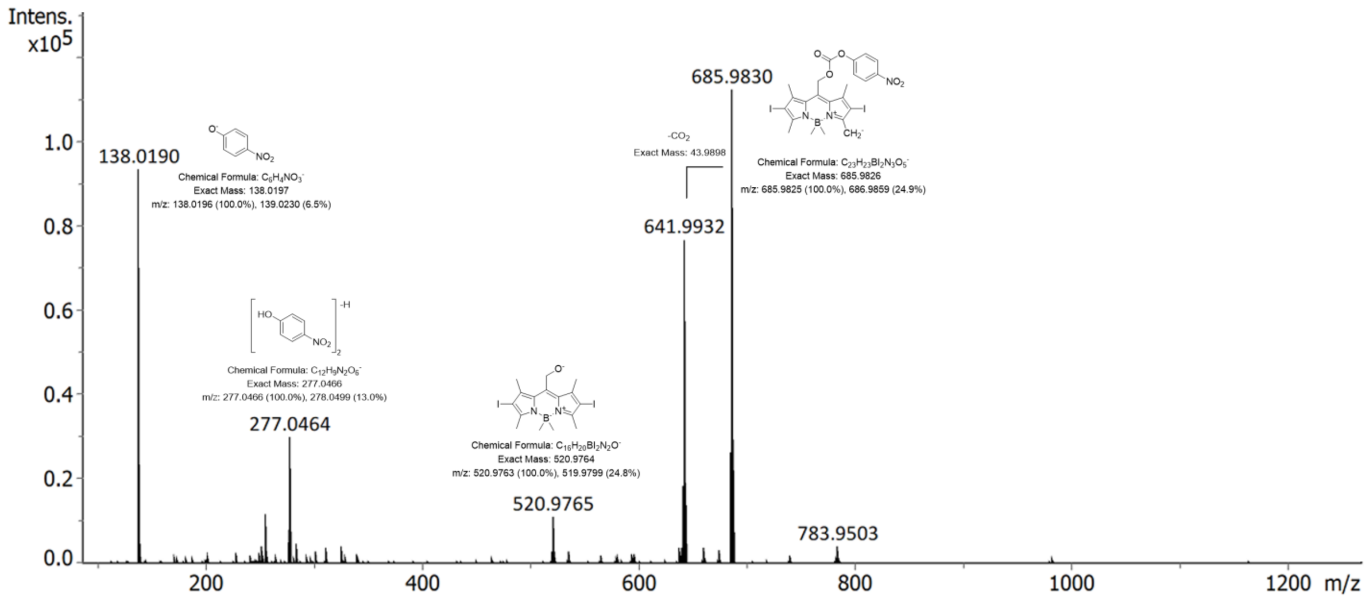

SI References

[1] N. Broguiere, I. Lüchtefeld, L. Trachsel, D. Mazunin, R. Rizzo, J. W. Bode, M. P. Lutolf and M. Zenobi-Wong, "Morphogenesis Guided by 3d Patterning of Growth Factors in Biological Matrices," *Adv. Mater.* **2020**, *32*, 1908299, https://doi.org/10.1002/adma.201908299.

[2] T. Imoto, A. Kawase, M. Minoshima, T. Yokoyama, H. Bito and K. Kikuchi, "Photolytic Release of a Caged Inhibitor of an Endogenous Transcription Factor Enables Optochemical Control of Creb-Mediated Gene Expression," *Org. Lett.* **2020**, *22*, 22-25, https://doi.org/10.1021/acs.orglett.9b03568.

[3] L. Donato, A. Mourot, C. M. Davenport, C. Herbivo, D. Warther, J. Léonard, F. Bolze, J.-F. Nicoud, R. H. Kramer, M. Goeldner and A. Specht, "Water-Soluble, Donor–Acceptor Biphenyl Derivatives in the 2-(O-Nitrophenyl)Propyl Series: Highly Efficient Two-Photon Uncaging of the Neurotransmitter Γ-Aminobutyric Acid at Λ=800 Nm," *Angew. Chem. Int. Ed.* **2012**, *51*, 1840-1843, https://doi.org/10.1002/anie.201106559.

[4] T. Slanina, P. Shrestha, E. Palao, D. Kand, J. A. Peterson, A. S. Dutton, N. Rubinstein, R. Weinstain, A. H. Winter and P. Klán, "In Search of the Perfect Photocage: Structure–Reactivity Relationships in Meso-Methyl Bodipy Photoremovable Protecting Groups," *J. Am. Chem. Soc.* **2017**, *139*, 15168-15175, https://doi.org/10.1021/jacs.7b08532.

[5] K.-Y. Chung and Z. A. Page, "Boron-Methylated Dipyrromethene as a Green Light Activated Type I Photoinitiator for Rapid Radical Polymerizations," *J. Am. Chem. Soc.* **2023**, *145*, 17912-17918, https://doi.org/10.1021/jacs.3c05373.

[6] K. Krumova and G. Cosa, "Bodipy Dyes with Tunable Redox Potentials and Functional Groups for Further Tethering: Preparation, Electrochemical, and Spectroscopic Characterization," *J. Am. Chem. Soc.* **2010**, *132*, 17560-17569, https://doi.org/10.1021/ja1075663.

[7] J. A. Peterson, L. J. Fischer, E. J. Gehrmann, P. Shrestha, D. Yuan, C. S. Wijesooriya, E. A. Smith and A. H. Winter, "Direct Photorelease of Alcohols from Boron-Alkylated Bodipy Photocages," *J. Org. Chem.* **2020**, *85*, 5712-5717, https://doi.org/10.1021/acs.joc.0c00044.

[8] T. Kremsmayr and M. Muttenthaler, "Fmoc Solid Phase Peptide Synthesis of Oxytocin and Analogues," *Methods Mol. Biol.* **2022**, *2384*, 175-199, https://doi.org/10.1007/978-1-0716-1759-5_11.

[9] K. Arunachalam and P. S. Sreeja, "Mtt Assay Protocol," in *Advanced Cell and Molecular Techniques: Protocols for in Vitro and in Vivo Studies*, ed. K. Arunachalam and P. S. Sreejas, (Springer US), New York, NY, **2025**, 271-276.

[10] L. Nørskov-Lauritsen, A. R. B. Thomsen and H. Bräuner-Osborne, "G Protein-Coupled Receptor Signaling Analysis Using Homogenous Time-Resolved Förster Resonance Energy Transfer (Htrf®) Technology," *Int. J. Mol. Sci.* **2014**, *15*, 2554-2572, https://doi.org/10.3390/ijms15022554

[11] A. D. Edelstein, M. A. Tsuchida, N. Amodaj, H. Pinkard, R. D. Vale and N. Stuurman, "Advanced Methods of Microscope Control Using Mmanager Software," *J. Biol. Methods* **2014**, *1*, https://doi.org/10.14440/jbm.2014.36.

[12] S. Melzer, E. R. Newmark, G. O. Mizuno, M. Hyun, A. C. Philson, E. Quiroli, B. Righetti, M. R. Gregory, K. W. Huang, J. Levasseur, L. Tian and B. L. Sabatini, "Bombesin-Like Peptide Recruits Disinhibitory Cortical Circuits and Enhances Fear Memories," *Cell* **2021**, *184*, 5622-5634.e5625, https://doi.org/10.1016/j.cell.2021.09.013.
